# Field model for multistate lateral diffusion of various transmembrane proteins observed in living *Dictyostelium* cells

**DOI:** 10.1242/jcs.260280

**Published:** 2023-02-20

**Authors:** Kazutoshi Takebayashi, Yoichiro Kamimura, Masahiro Ueda

**Affiliations:** ^1^ Graduate School of Science, Osaka University, Toyonaka, Osaka, 560-0043, Japan; ^2^ Center for Biosystems Dynamics Research (BDR), RIKEN, Suita, Osaka, 565-0874, Japan; ^3^ Graduate School of Frontier Biosciences, Osaka University, Suita, Osaka, 565-0871, Japan

**Keywords:** Lateral diffusion, Transmembrane protein, Single-molecule imaging, Hidden Markov model, Raft

## Abstract

The lateral diffusion of transmembrane proteins on plasma membranes is a fundamental process for various cellular functions. Diffusion properties specific for individual protein species have been extensively studied, but the common features among protein species are poorly understood. Here, we systematically studied the lateral diffusion of various transmembrane proteins in the lower eukaryote *Dictyostelium discoideum* cells using a hidden Markov model for single-molecule trajectories obtained experimentally. As common features, all membrane proteins that had from one to ten transmembrane regions adopted three free diffusion states with similar diffusion coefficients regardless of their structural variability. All protein species reduced their mobility similarly upon the inhibition of microtubule or actin cytoskeleton dynamics, or myosin II. The relationship between protein size and the diffusion coefficient was consistent with the Saffman–Delbrück model, meaning that membrane viscosity is a major determinant of lateral diffusion, but protein size is not. These protein species-independent properties of multistate free diffusion were explained simply and quantitatively by free diffusion on the three membrane regions with different viscosities, which is in sharp contrast to the complex diffusion behavior of transmembrane proteins in higher eukaryotes.

## Teaser

**Summary:** The complex diffusion behaviors of various transmembrane proteins in *Dictyostelium* cells can be explained quantitatively by free diffusion on three membrane regions of different viscosities.

## INTRODUCTION

Understanding the mechanisms that determine diffusional mobility is an important issue in biology in general. Since reporting of the fluid mosaic model (Singer and Nicolson, 1972), various models of the cell membrane have been proposed to explain the determinants of lateral diffusion based on the membrane structure. The cell membrane contains and interacts with many elements that influence diffusional properties, such as lipid rafts, cytoskeletal fences and the extracellular matrix (Saxton and Jacobson, 1997; Simons and Ikonen, 1997; Simons and Toomre, 2000; Fujiwara et al., 2002; Murase et al., 2004). The accumulated knowledge of diffusional properties of various membrane proteins in different cell types has revealed various diffusional modes, such as simple, anomalous and confined diffusion, and their heterogeneity (Saxton and Jacobson, 1997; Kusumi et al., 2005, 2014). The different diffusion modes have been explained by the complexity of the membrane structure and protein species-specific interactions with the elements (Kusumi et al., 2005, 2014). Although there has been abundant research on diffusion properties specific for individual protein species, there has been little research on common features between species. Consequently, it is difficult to determine whether a given diffusional movement is caused by a protein species-specific feature or by the surrounding membrane environment. In order to develop a model widely applicable to various membrane proteins, it is important to dissect two aspects of the diffusion: one derived from the intrinsic properties specific to the protein species and the other derived from the membrane environment surrounding the proteins.

To understand the common features in diffusion regardless of the proteins, we systematically analyzed the lateral diffusion of many transmembrane proteins with structural variability in the same membrane condition. For this, we used the lower eukaryote *Dictyostelium discoideum* as a model, given that these cells have been established for the single-molecule imaging of membrane proteins (Ueda et al., 2001; Matsuoka et al., 2006, 2016; Miyanaga et al., 2009). We found that all proteins underwent free diffusion with similar diffusion coefficients despite the 10-fold difference in the number of transmembrane regions. We propose a simple field model of the membrane structure that can quantitatively explain the experimental observations, in which heterogeneity in membrane viscosity determines the multistate free diffusion of transmembrane proteins. The proposed membrane field model suggests a simple membrane structure for multistate lateral mobility.

## RESULTS AND DISCUSSION

### All transmembrane proteins undergo free diffusion

For a systematic analysis of the lateral diffusion of transmembrane proteins, we selected 143 membrane proteins of *D. discoideum* that are annotated as having α-helix transmembrane regions embedded in the plasma membrane (Table S1). We prepared the tagged proteins with a HaloTag at the C-terminus for single-molecule imaging (Los et al., 2008; Matsuoka et al., 2016). When introduced into wild-type AX2 cells, only 27 proteins exhibited stable expression on the plasma membrane, and the corresponding stable transformants were obtained for the subsequent diffusion analysis. The proteins had between one and ten transmembrane domains, as estimated by UniProtKB (Table S1). After the tag was stained by a fluorescent Halo-ligand conjugated to tetramethylrhodamine, non-polarized vegetative cells were observed under total internal reflection fluorescence microscopy (TIRFM), and the images were acquired at 30 frames/s.

**Fig. 1.**
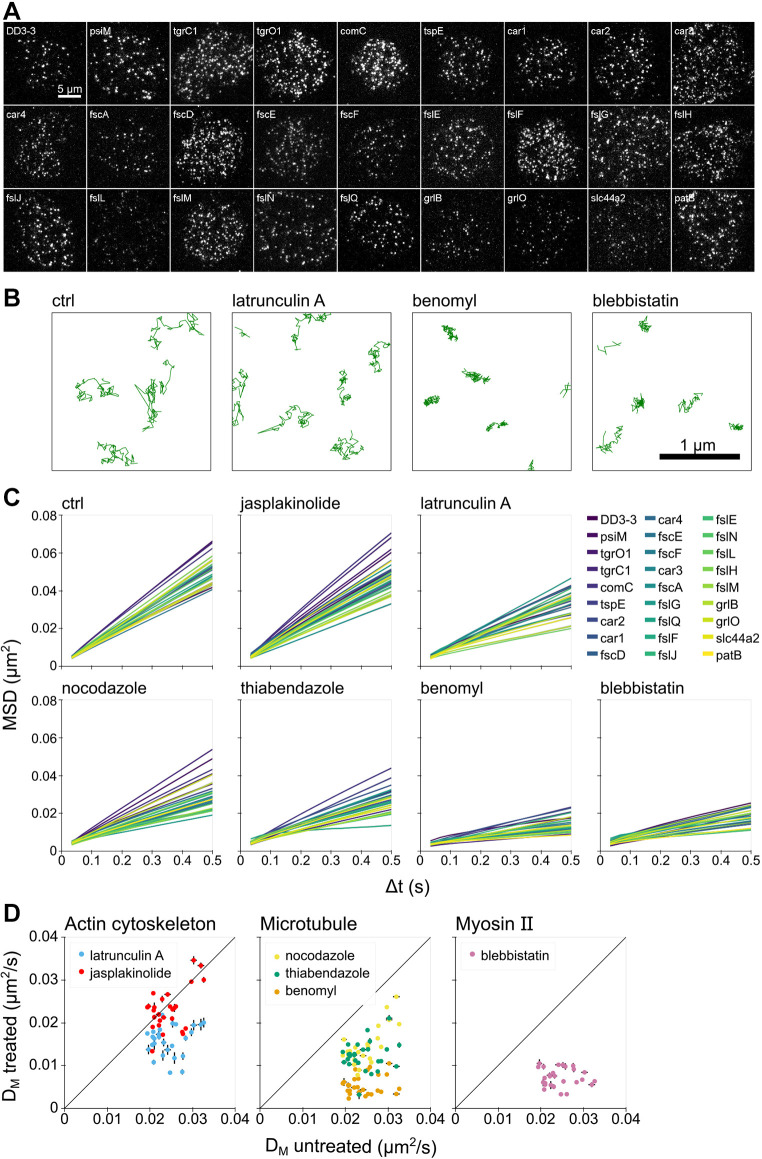
**Transmembrane proteins freely diffuse in the plasma membrane.** (A) Single-molecule images of 27 kinds of transmembrane proteins in *D. discoideum* cells. Images are representative of ten repeats. (B) Representative trajectories from ten repeats of the lateral diffusion of one membrane protein (fslN) in each drug condition. (C) MSDs of the 27 membrane proteins in each drug condition are shown in different colors. (D) Scatter plots of diffusion coefficients (*D*_M_, mean±95% c.i.) estimated from the MSDs. Diagonal lines indicate no effect on *D*_M_. (*n*=10 bootstrap datasets created from trajectory data of ten cells).

Individual membrane proteins exhibited lateral diffusion on the basal membrane of the cells (Fig. 1A; Movie 1). To characterize the diffusion modes, single-molecule trajectories were obtained and analyzed by calculating the mean square displacement (MSD) (Fig. 1B,C). The MSDs were linear with time for all species, indicating that all proteins underwent free diffusion. The diffusion coefficients ranged from 0.019 to 0.033 µm^2^/s (Table S2). Despite the structural variability within the 1–10 transmembrane regions and 27 to 163 kDa molecular mass, the obtained diffusion coefficients were not very different from each other. The average diffusion coefficient for all 27 proteins was 0.024±0.004 µm^2^/s (mean±s.d.), which is about one-tenth that measured for the free diffusion of various membrane proteins in mammalian cells (Mashanov et al., 2021), showing that there is an apparently higher viscosity of the membrane of *Dictyostelium* cells than of mammalian cells. In addition, the observation that all protein species underwent simple diffusion is a characteristic feature in this lower eukaryote that distinguishes it from the diverse diffusion modes in mammalian cells.

The possible involvement of the cytoskeleton in the mobility of membrane proteins was examined using inhibitors for actin and microtubules, because the cytoskeleton influences lateral diffusion in mammalian cells (Kusumi et al., 2005, 2014). We used latrunculin A and jasplakinolide to inhibit actin polymerization and depolymerization, respectively, and used nocodazole, thiabendazole and benomyl to disrupt microtubules (de Keijzer et al., 2011; Miyanaga et al., 2018). The inhibition of the cytoskeleton dynamics was confirmed by phalloidin staining for actin and immunofluorescence staining for microtubules (Fig. S1). All molecular species underwent free diffusion when any drug was applied (Fig. 1B,C). The MSDs of all proteins were close to each other in each drug condition, showing a consistent trend for the drug-induced changes in diffusion regardless of the molecular species. All proteins showed reduced diffusion for all four cytoskeleton inhibitors except jasplakinolide (Fig. 1D; Table S2). In addition, we investigated whether myosin II affects the diffusion by using the inhibitor blebbistatin. All proteins underwent slower free diffusion with the drug than without (Fig. 1B–D). Thus, the cytoskeleton contributes to lateral diffusion in *Dictyostelium* cells, probably by factors that act in common with various protein species rather than intrinsic factors specific to the protein species. Moreover, the reduced diffusion suggests that cytoskeletal ‘fences’ do not play a major role in determining diffusion properties in *Dictyostelium* cells.

### All transmembrane proteins undergo multistate free diffusion with similar diffusion coefficients

**Fig. 2.**
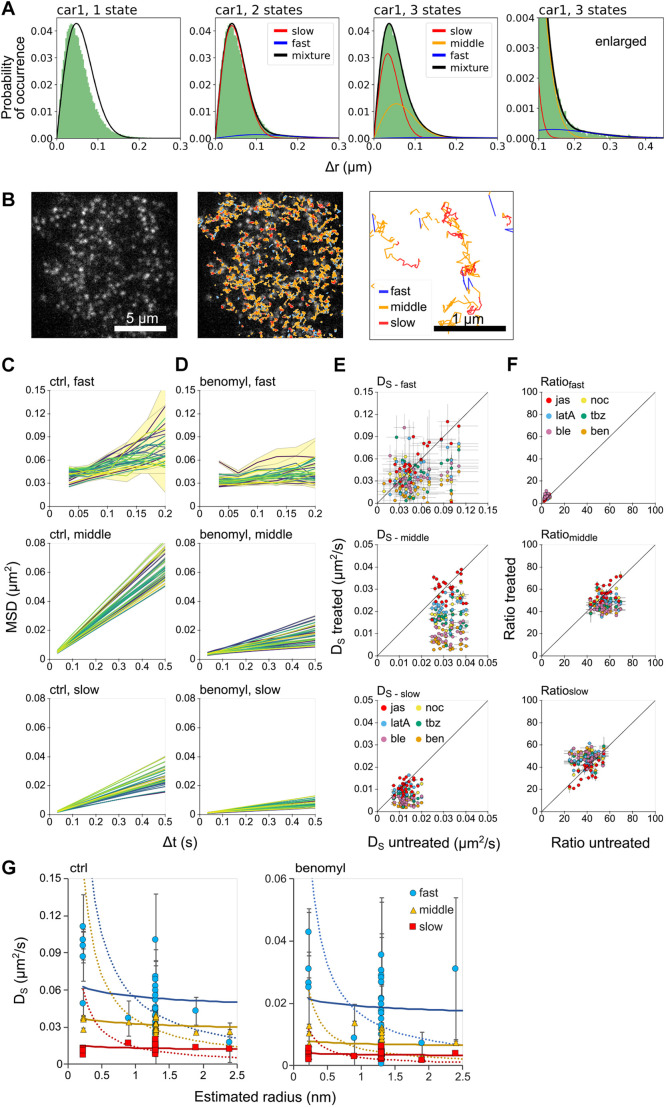
**Lateral diffusion of membrane proteins is defined by three different states.** (A) Histograms of displacements (Δ*r*) at 33-ms intervals (green) overlaid with a PDF of freely diffusing molecules with one to three diffusion states (black). Parameters of the PDFs were determined from datasets of Δ*r* by MLE. The PDFs corresponding to each diffusion state are indicated by the fast (blue), middle (yellow), slow (red) lines. The bin size was 3 nm. (B) Representative single-molecule images from ten repeats (car4) (left). Overlaid trajectories categorized into three different states: fast (blue), middle (yellow) and slow (red) by the HMM (middle). An enlarged view of the trajectories (right). (C,D) The MSD of each diffusion state of the 27 membrane proteins are shown for each different state (top, middle and bottom) under control (C) and benomyl-treated conditions (D). Colors are the same as for Fig. 1C. (E,F) Scatter plots of the diffusion coefficients (*D*_S_, mean±95% c.i.) (E) and ratios (mean±95% c.i.) (F) for each state (top, middle, bottom) under drug-treated conditions. Diagonal lines indicate no effect on the diffusion coefficients. *n*=10 bootstrap datasets created from trajectory data of ten cells. (G) Diffusion coefficients (*D*_S,_ mean±95% c.i., *n*=10 bootstrap datasets created from trajectory data of ten cells) for the fast (blue), middle (yellow), and slow states (red) of the 27 membrane proteins are plotted over the estimated radii of the proteins. The solid and dotted lines show theoretical states derived from the Saffman–Delbrück model and Stokes–Einstein-like model, respectively.

Individual proteins of all species observed under TIRFM exhibited a transition in lateral mobility, as observed previously in mammalian cells (Low-Nam et al., 2011; Hiroshima et al., 2018; Yanagawa et al., 2018; Clarke and Martin-Fernandez, 2019). To further characterize the diffusion properties, we analyzed the displacement distribution using a mixed Gaussian function (Matsuoka et al., 2009, 2016). A one-state model of free diffusion was not consistent with the experimental data (Fig. 2A; Fig. S2A). The model selection using the Akaike information criterion (AIC) (Akaike, 1974), which can estimate the number of diffusion states, found that three states were optimal for any protein species (Fig. 2A; Fig. S2). Then we analyzed the single-molecule trajectories by classifying them into three states (fast, middle, and slow) using a hidden Markov model (HMM), as reported previously (Hiroshima et al., 2018; Yanagawa et al., 2018). This analysis can assign three states along the trajectories (Fig. 2B). By calculating the MSDs from the displacement for each state, we examined the diffusion modes for each state of all molecular species. We found that all proteins observed exhibited simple free diffusion in each state under control and inhibitor-treated conditions (Fig. 2C,D; Fig. S3A), which shows a marked difference from the multi-modal mobility seen in mammalian cells (Hiroshima et al., 2018; Yanagawa et al., 2018). Reduced mobility was observed in the middle- and slow-mobile states for all inhibitors except jasplakinolide and tended that way for the fast state (Fig. 2E; Table S3). The middle- and slow-mobile states ranged from 42.8–69.4% and 24.6–54.7% for various proteins under control conditions, respectively, whereas the fast-mobile state (2.1–7.2%) was a minor subpopulation. The ratio of each state was relatively maintained with any inhibitor (Fig. 2F; Table S3). Thus, three diffusion states were a common feature for all protein species. This observation can be explained by simple diffusion on a heterogeneous membrane structure shared among membrane proteins and the drug-dependent modulation.

Two general physical models have been proposed to explain the lateral diffusion of membrane proteins (Saffman and Delbrück, 1975; Gambin et al., 2006). In the Saffman–Delbrück model, the diffusion coefficient is inversely proportional to the membrane viscosity, *µ_m_*, and the log of the radius, *R*, of the protein embedded in the membrane (Eqn 8 in Materials and Methods), meaning that protein size is not a major determinant for lateral diffusion. In the Stokes–Einstein-like model, the diffusion coefficient is inversely proportional to *µ_m_* and *R* (Eqn 9 in Materials and Methods). To evaluate the relationship between the structural variability and the measured diffusion coefficients based on these models, the radius of the protein species was estimated from the predicted structures of the transmembrane regions (Jumper et al., 2021; Varadi et al., 2021). This estimation did not take into account the oligomerization of the molecules and treats them as monomers. Fig. 2G shows that the middle- and slow-mobile states were more consistent with the Saffman–Delbrück model than the Stokes–Einstein-like model, meaning that the radius diversity ranging from 1 to 10 transmembrane regions had no significant contribution to the diffusion of these two states. Given that all proteins adopted the middle- or slow-mobile states for more than 92% of trajectories under all conditions measured, the lateral mobility of membrane proteins was determined primarily by differences in membrane viscosity and not the intrinsic structural variability of the proteins. We estimated *µ_m_* by fitting to the Saffman–Delbrück model as 29.7±0.3 and 80.4±1.7 (mean±95% c.i.) Pa·s for the middle- and slow-mobile states, respectively. The estimated *µ_m_* of the fast state (16.7±0.7 Pa·s) was also larger than estimated previously in mammalian cells (∼1.0 Pa·s) (Kashirina et al., 2020), meaning that viscosity is also a major determinant for the fast-mobile state. These results show three mobility states reflecting free diffusion in three membrane fields with different viscosities.

**Fig. 3.**
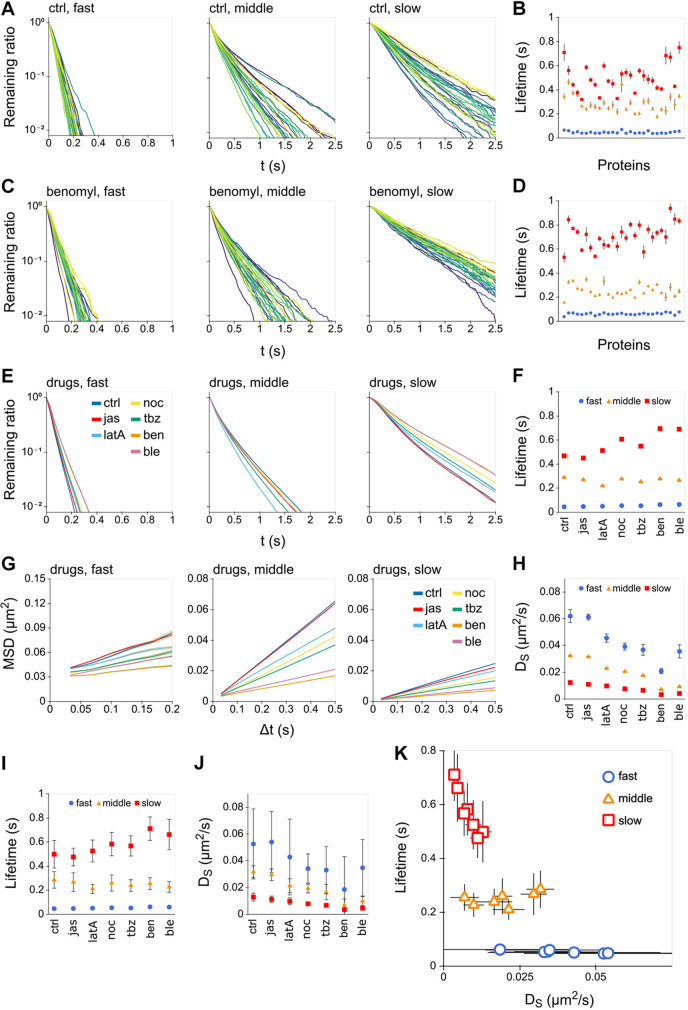
**Each of the three diffusion states has a unique lifetime.** (A) Statistical distributions of the lifetimes are shown for each diffusion state of the 27 membrane proteins represented in different colors. (B) The average lifetimes obtained by fitting the decay curves to exponentials (mean±95% c.i.) (*n*=10 bootstrap datasets created from trajectory data of ten cells). (C,D) The lifetimes under the benomyl-treated condition (C) and average lifetimes (mean±95% c.i.) (*n*=10 bootstrap datasets created from trajectory data of ten cells) (D). (E–H) The lifetimes (E), average lifetimes (mean±95% c.i.) (F), MSD (G) and *D*_S_ (mean±95% c.i.) (H) were obtained from summing the data of the 27 proteins under control and drug-treated conditions, shown in different colors (*n*=10 bootstrap datasets created from trajectory data of 27 proteins). (I,J) The average lifetimes (I) and *D*_S_ (J) for the 27 proteins (mean±s.d.). (K) The relationship between *D*_S_ and lifetimes as a scatter plot (mean±s.d.) (*n*=27 proteins).

An HMM analysis of single-molecule trajectories can provide time duration data for each state, from which the lifetimes of each state can be obtained (Fig. 3A). Because the lifetimes reflect the escape from the corresponding diffusion area, the longer lifetimes mean a larger area. For each of the 27 proteins, the middle- and slow-mobile states exhibited longer lifetimes than those of the fast-mobile state (Fig. 3B). Benomyl-treated cells exhibited shorter, intermediate and longer lifetimes for the fast-, middle- and slow-mobile states, respectively, which is similar to that for the untreated control (Fig. 3C,D). The same tendency was also observed under all other drug-treated conditions (Fig. S3B; Table S4). The average lifetimes and diffusion coefficients of the three states independent of the protein species were determined from summing the trajectory data of all proteins to surmise the overall tendency (Fig. 3E–H; Table S5). The average lifetimes for each of the 27 proteins were also determined to examine protein-specific variations (Fig. 3I,J). Scatter plots of the averaged lifetimes and diffusion coefficients indicate three discrete states with relatively constant lifetimes and drug-dependent mobility (Fig. 3K). These results imply that three diffusion fields are maintained at their average sizes but with changes in their viscosities by the drug treatment. The HMM also revealed almost no transition between the fast- and slow-mobile states, suggesting physical separation of the membrane fields corresponding to these two states (Fig. S4; Table S6).

### Field model for multistate lateral diffusion of various transmembrane proteins

**Fig. 4.**
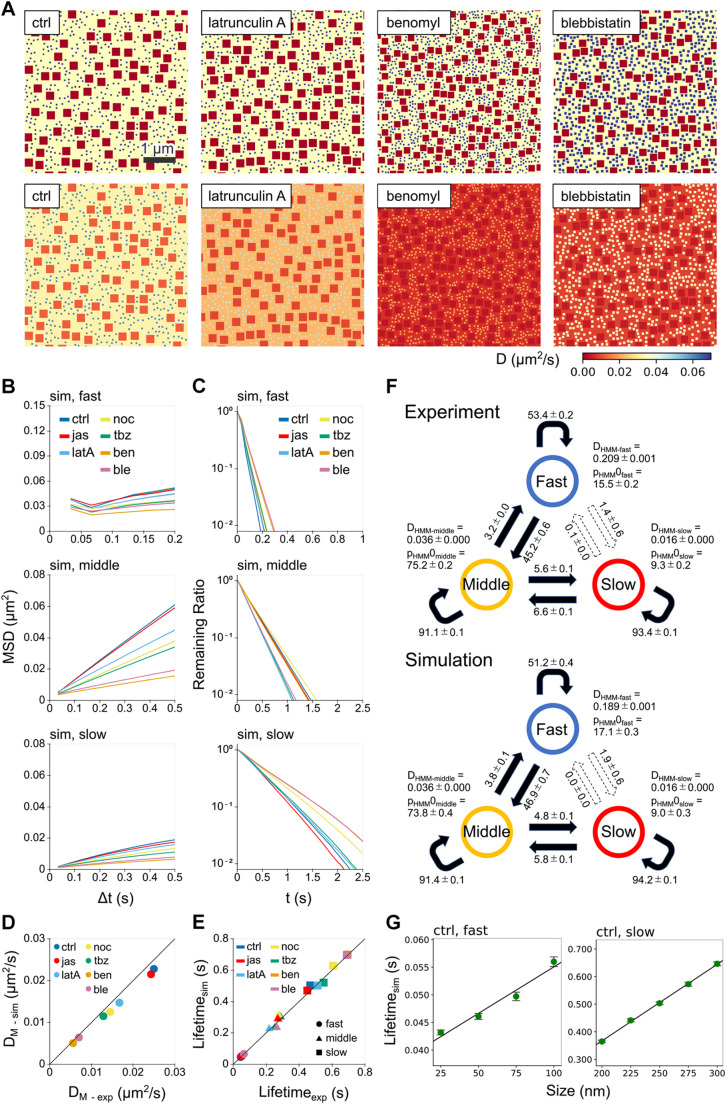
**Field models of the membrane recapitulate the heterogeneous mobility of many membrane proteins observed experimentally.** (A) Field models with three distinct regions corresponding to three diffusion states under the indicated drug treatments (upper panels). The fast, middle and slow states are represented by the blue, yellow and red areas, respectively. The fields are redisplayed as a heat map of the diffusion coefficients for each region (lower panels). The size of the fields is 5 µm×5 µm. (B,C) Reproduced mobility of particles in the field models represented as the MSD (B) and lifetimes (C) in different colors for the indicated conditions. (D,E) Comparison of the reproduced mobility with the experimental data for diffusion coefficients (D) and lifetimes (E) (mean±95% c.i.; 95% c.i. values are smaller than the sizes of the markers) (*n*=10 datasets). (F) State transitions from the experimental and simulated data using the HMM under control conditions. Numbers represent rate constants. *D*_HMM-*state*_ and p_HMM_0*_state_* represent diffusion coefficients and initial probabilities occupying the corresponding states, respectively. (G) Relationship between field sizes and lifetimes in field model under control conditions. Mean±95% c.i. *n*=10 datasets.

To account for the multistate free diffusion commonly observed in many transmembrane proteins, we proposed a simple membrane field model. We assumed that the membrane field consists of three regions corresponding to fast, middle and slow simple diffusion, in which fast- and slow-mobile squares are placed randomly on the basement of the middle-mobile region (Fig. 4A; Fig. S5). The fast and slow regions are not adjacent to each other. Each particle undergoes simple diffusion in each region with the corresponding diffusion coefficients that were experimentally determined (Fig. 3G,H; Table S7). By setting only four parameters that include the sizes of the fast and slow regions and their occupied percentage area relative to the whole field, this model can successfully reproduce the multi-state diffusion under the control and drug-treated conditions (Fig. 4B–F; Tables S5, S6). The sizes of the fast and slow regions were determined by the simulation of the particle diffusion on the field. Because larger sizes result in longer lifetimes, the region sizes were set based on the lifetimes of the fast- and slow-mobile states obtained experimentally (Fig. 4G). For example, the region sizes were set to 50 and 250 nm for the fast- and slow-mobile states, respectively, under control conditions (Table S7). Then we searched for the occupied percentage area of the fast and slow regions for which the MSD and HMM results from the particle trajectories matched the measured values (Tables S5, S7). The field model we propose here was consistent with the data of the MSD (Fig. 4D), lifetimes (Fig. 4E) and HMM (Fig. 4F; Fig. S4, Table S6). The numerical simulation robustly reproduced the diffusion characteristics when the shape of the fast and slow regions was not changed extremely (Fig. S6).

### Conclusions

From the systematic analyses of the lateral diffusion of various transmembrane proteins in *Dictyostelium* cells, two straightforward conclusions can be drawn: (1) all transmembrane proteins undergo free diffusion, demonstrating no various modes of diffusion, and (2) they all adopt three states of free diffusion with similar diffusion coefficients, demonstrating that the intrinsic properties of proteins, such as their molecular size, are not a major determinant for lateral mobility, whereas the membrane environments surrounding the proteins are. These observations are consistent with the Saffman–Delbrück model, in which heterogeneity in membrane viscosity is a major determinant of lateral mobility. The proposed membrane field model suggests that *Dictyostelium* cells have a relatively simple membrane structure capable of producing multi-state free diffusion. Because the HMM itself is spatially independent, the three diffusion states might be explained by binding and unbinding with various partners as an alternative model. Among those partners, lipids are most likely to affect the diffusion of membrane proteins because lipids can form microdomains with different viscosities. What particular structure of the membrane corresponds to each mobility region in *Dictysotelium* remains unknown. However, the size of the slow region is close to that of lipid rafts in mammalian cells (Pike, 2006), suggesting relevance. Multiple microdomains with different viscosities can generate coarseness and density in the spatial distribution of membrane proteins by diffusion (Movie 2), which is the original concept of the raft hypothesis (Simons and Ikonen, 1997). It will be important to clarify the functional significance of the relatively simple diffusion dynamics observed here in terms of the physiology of *Dictyostelium* cells.

## MATERIALS AND METHODS

### Selection of candidates for single-molecule measurements

We focused on the molecular species of membrane proteins with α-helix transmembrane regions in the plasma membrane of *Dictyostelium discoideum*. In order to include as many protein species as possible in the candidate list, UniProtKB was used for the selection (The UniProt Consortium, 2021). We selected molecular species registered in SwissProt, which are manually annotated and reviewed among the UniProtKB entries. There were 430 registered molecular species of membrane proteins with annotated sequences of α-helix transmembrane regions. Among them, 143 species with annotations for the extramembrane region (cytoplasmic, extracellular), which are likely to exist on the cell membrane, were selected as candidates for the measurements. Of the 27 membrane proteins that exhibited stable expression on the plasma membrane of wild-type AX2 cells, no molecules had been reported to interact specifically with cytoskeleton molecules, such as microtubules or F-actin.

### Cell culture and DNA constructs

*Dictyostelium discoideum* cells were used for all experiments. Ax2 was used as the wild-type parental strain (in-house strain). Cells were statically cultured at 21°C in HL5 medium including 15.4 g/l glucose, 7.15 g/l yeast extract (Oxoid), 14.3 g/l proteose peptone No. 2 (BD Biosciences), 1.28 g/l Na_2_HPO_4_·12H_2_O, 0.486 g/l KH_2_PO_4_, 200 mg/l folic acid, 0.06 mg/l cyanocobalamin, 6 ng/ml vitamin B12 supplemented with 100 mg/l streptomycin sulfate, and 70 mg/l benzylpenicillin potassium, as reported previously (Watts and Ashworth, 1970; Kamimura et al., 2016). Plasmids were generated by In-Fusion (Takara Bio Inc). The plasmid vector, pHK12-neo-C-terminal Halo, was digested using BglII. The genes encoding the membrane proteins were amplified by PCR with Phusion High Fidelity DNA Polymerase (NEB) from *Dictyostelium* genomic DNA. Primers were the first and last 20–30 nucleotides carrying an additional 15 nucleotides of the flanking vector sequences at the BglII digested site for the In-Fusion cloning. All primer sequences are listed in Table S1. The resultant plasmids allowed the expression of membrane proteins bound to HaloTag^®^ (Promega) at the C-terminus. The expression plasmids were electroporated into *Dictyostelium* cells using the ECM 830 Square Wave Electroporation System (BTX) at the following setting: an effective voltage of 500 V, a pulse width of 100 μs, a pulse interval of 1.0 s, and pulse number of 15. A total of 10^7^ cells were exponentially grown in a Petri dish, then HL5 medium was removed, and the cells were washed with development buffer (DB; 5 mM NaH_2_PO_4_, 5 mM Na_2_HPO_4_, 2 mM MgSO_4_, 0.2 mM CaCl_2_) three times. Then the cells were collected from the dish in 1 ml electroporation buffer (10 mM KH_2_PO_4_, 50 mM Sucrose), and 400 μl was taken and mixed with 5 μg plasmid DNA. The mixture was placed into a pre-chilled cuvette and allowed to stand for 5 min on ice. After electroporation, cell–DNA mix was transferred to a new Petri dish and mixed with 4 μl healing buffer (100 mM CaCl_2_ and 100 mM MgCl_2_). After 15 min, 10 ml HL5 buffer (1.28 g/l Na_2_HPO_4_·12H_2_O, 0.486 g/l KH_2_PO_4_) and 10 ml HL5 medium were added to the electroporated cells. After 24 h of incubation, G418 (Nacalai tesque) was added to the medium at a final concentration of 10 μg/ml to select transformed cells.

### Phalloidin staining to observe actin cytoskeleton

After 1 h starvation in DB, the cells were incubated with one of 2.5 μM jasplakinolide (Abcam), 5 μM latrunculin A (Sigma-Aldrich), 50 μM nocodazole (Abcam), 100 μM thiabendazole (Santa Cruz Biotechnology), 20 μM benomyl (Riedel-de Haen) or 100 μM blebbistatin (Sigma-Aldrich) in DB for 30 min. The drugs were removed, and the cells were fixed with 3.7% formaldehyde in DB for 30 min at 21°C. After washing with DB, the cells were treated with 0.1% Triton X-100 in DB for 5 min and washed with PBS twice. The cells were stained with BODIPY-conjugated phalloidin (Invitrogen) diluted 200 times in PBS for 30 min at 21°C. After three washes with PBS, the cells were incubated in PBS. Images were taken using an Olympus FV1000 confocal laser microscope system with an oil immersion objective lens (Fluor 60×/1.49 NA).

### Microtubule staining

Cells treated with drugs were prepared for the phalloidin staining above. The drugs were removed, and the cells were fixed with methanol pre-chilled at −30°C for 5 min. After two quick rinses with PBS, the cells were incubated in PBS for 15 min and subsequently in PBS containing 5 mg/ml BSA for 15 min at 21°C. The cells were incubated with 1:400 anti-α-tubulin antibody labeled with FITC (Sigma-Aldrich, F2168) in PBS containing 5 mg/ml BSA at 4°C overnight. After washing with PBS three times, the cells were filled in a 1:1 dilution of an antifade reagent using PBS (Nacalai tesque, 12745-74). Images were taken using the Olympus FV1000 confocal laser microscope system with an oil immersion objective lens.

### Preparation for live single-molecule imaging

For single-molecule imaging of the transmembrane proteins in living cells, we used HaloTag^®^, which can be fluorescently labeled by Halo-ligands. A Halo-ligand, tetramethylrhodamine (TMR; Promega), was used for all measurements, as previously described (Miyanaga et al., 2009; Matsuoka et al., 2016). The cells were prepared as follows. About 5×10^6^ cells carrying the membrane protein expression plasmids cultured in a 90-mm Petri dish were transferred to a 35-mm Petri dish. HL5 medium was removed, and the cells were washed with DB three times followed by a 1-h incubation in 1 ml DB. To the dish, 5 µl of 10 μM HaloTag TMR ligand was added, and the mixture was incubated for 30 min. After the staining solution was removed, and the cells were washed with DB three times. The cells were transferred to a 96-well glass bottom plate (Greiner) and allowed to stand for 5 min. Drugs were added just before transferring to the 96-well plates. For the drug treatment, one of 2.5 µM jasplakinolide, 5 µM latrunculin A, 50 µM nocodazole, 100 µM thiabendazole, 20 µM benomyl or 100 µM blebbistatin was added to the cells at final concentrations under the respective conditions (Clarke et al., 2002; Shu et al., 2005; Tang et al., 2008; Yumura et al., 2014; Sugiyama et al., 2015). The plate was then centrifuged at 500 ***g*** for 2 min for the cells to adhere to the glass surface of the plate.

### Single-molecule imaging by TIRFM

Single-molecule imaging was performed as described previously using an inverted fluorescence microscope (ECLIPSE Ti2-E, Nikon) (Miyanaga et al., 2009; Matsuoka et al., 2016). The objective lens was a 60× lens (CFI Apochromat TIRF 60XC Oil, Nikon), and, together with a 1.5× intermediate magnification unit, the magnification was 90×. A laser of wavelength 561 nm with an output power of 150 mW (OBIS 561-150 LS, Coherent) was used to excite the fluorescent ligands. Images were acquired using a CMOS camera (C13440-20CU, HAMAMATSU). Movies were captured at a size of 768×768 pixels for 100 frames at a rate of 30 frames/s. The pixel size was 72 nm. Images were taken of five cells for each drug condition of each membrane protein species twice on different days to obtain a total of 10 cell movies. From the single-molecule observation of 10 cells for each experimental condition, we obtained ∼3000 single-molecule tracks, providing ∼80,000 displacements at 33-ms intervals for the diffusion analysis.

### Tracking of fluorescence signals

Trajectories of the fluorescence signals from single molecules were automatically acquired from single-molecule imaging movies using the Fiji plugin TrackMate (Tinevez et al., 2017). After manually selecting a region of interest (ROI) for each movie, the Laplacian of Gaussian (LoG) detector was used for light spots detection, and the Linear Assignment Problem (LAP) tracker was used for frame-to-frame particle linking. Other parameters were set as follows: DO_SUBPIXEL_LOCALIZATION=true; RADIUS=3.0 pixels; THRESHOLD=2.0; DO_MEDIAN_FILTERING=false; ALLOW_TRACK_SPLITTING=false; ALLOW_TRACK_MERGING=false; LINKING_MAX_DISTANCE=6.0 pixels; GAP_CLOSING_MAX_DISTANCE=30 pixels for ESCs, MES/60 pixels for NPCs; MAX_FRAME_GAP=0 frames; TRACK_FILTER=TRACK_DURATION: 3.

A histogram of trajectory lengths is shown in Fig. S7. The mean and s.d. of the track durations were 23.9±28.9 frames (Fig. S7A). Durations more than 1 s constituted more than 70% of the total data (Fig. S7B). The diffusion parameters could be estimated by the HMM even in the presence of the trajectory interruption (Fig. S7C,D). The probability to estimate the three diffusion states correctly by the HMM was ∼80% (Fig. S7F).

### Diffusion analysis – MSD and PDF

The mean squared displacement (MSD) was used to obtain the diffusion coefficient, *D*_M_, of the molecules (Miyanaga et al., 2009; Matsuoka et al., 2016). The MSD of molecular motion is calculated as follows:
(1)

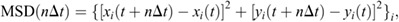
where *i* is the number of the trajectory, *x_i_*(*t*) and *y_i_*(*t*) are the *x*- and *y*-coordinates of the *i*-th trajectory at time point *t*, respectively, Δ*t* is the time interval between frames (33 ms), and *n* represents the frame number. The MSD was calculated with *n* ranging from 1 to 15. { }*_i_* means the average of *i* trajectories.

For two-dimensional free diffusion, the MSD is expressed using *D*_M_ and *ε*_M_, which represent the diffusion coefficient and the localization error of the position of the molecule, respectively, as follows:
(2)

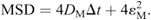
The MSD calculated from Eqn 1 was linearly approximated by the least-squares method, and *D*_M_ and *ε*_M_ were calculated from Eqn 2. The values for *ε*_M_ were 12–28 nm for the MSD analysis when the trajectories were not divided into multiple diffusion states (Table S2), which is in good agreement with previous reports (Matsuoka et al., 2009, 2016).

The probability density function (PDF) of the displacement of the molecules and the Akaike information criterion (AIC) were used to estimate the number of diffusion states of the molecules (Akaike, 1974; Matsuoka et al., 2009, 2016). The PDF of the displacement of freely diffusing molecules with multiple diffusion states is expressed by the following equation:
(3)

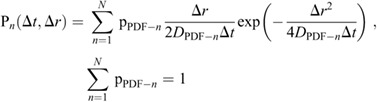
where Δ*r* is the displacement between frames, Δ*t* is the time interval between frames (33 ms), *N* is the number of diffusion states, p_PDF-*n*_ is the ratio of the diffusion state *n*, and *D*_PDF-*n*_ is the diffusion coefficient of the diffusion state *n*. The PDF does not include localization errors, because the errors vary depending on the diffusion state, and the errors for each diffusion state can be determined only after the trajectory has been separated into multiple states by the HMM analysis and then analyzed using the MSD, as described below (also see Table S3). Thus, *D*_PDF-*n*_ is the apparent diffusion coefficient and used only to estimate the number of diffusion states.

To calculate AIC values, a maximum likelihood estimation (MLE) of the parameter *θ* is required for each model, and the log likelihood L(*θ*) can be described as follows:
(4)

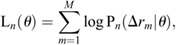
where *M* is the total number of all Δ*r* obtained from the single-molecule trajectory data. *θ* is estimated from the dataset of all Δ*r*, assuming that the dataset is derived from the PDF with *θ* unknown. The values of *θ* are searched so as to return the maximum log-likelihood. From these MLE, we can obtain the apparent diffusion coefficient *D*_PDF-*n*_ and the ratio p_PDF-*n*_ as well as L(*θ*) for the four models (*N*=1 to 4). The appropriate number of diffusion states was determined by calculating the AIC*_n_* from the calculated log likelihood (Akaike, 1974; Matsuoka et al., 2009, 2016):
(5)

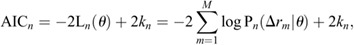
where *k_n_* denotes the number of parameters used for the model. The model with the number of states *N* that minimizes the value of the AIC is the optimal model.

Because three states were optimal for any protein based on this analysis, the estimated apparent diffusion coefficients and the ratios were expressed as *D*_PDF-fast_, p_PDF-fast_, *D*_PDF-middle_, p_PDF-middle_ and *D*_PDF-slow_, p_PDF-slow_ for fast, middle and slow states, respectively, as summarized in Table S8.

### Hidden Markov model

Single-molecule trajectories were analyzed using the HMM, as reported previously (Hiroshima et al., 2018; Yanagawa et al., 2018). The number of states of the HMM used in the analysis was 3. The model parameters had initial probabilities p_HMM_0_fast_, p_HMM_0_middle_, p_HMM_0_slow_, a transition probability matrix A, and observed symbol probability distributions of *B*_fast_, *B*_middle_ and *B*_slow_. Given that the molecules are freely diffusing in each state, *B_state_* can be expressed as follows:
(6)

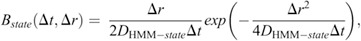
where the state is fast, middle or slow. Given that the only parameter of the distribution *B* is the diffusion coefficient, *D*_HMM-*state*_ was used as the model parameter. The initial values of the initial probabilities (p_HMM_0_fast_, p_HMM_0_middle_, p_HMM_0_slow_) and the transition probability matrix A were determined randomly. The initial value of *D*_HMM-*state*_ was determined by dividing Δ*r*^2^/4Δ*t* of each dataset into three clusters using the k-means method, and the central value of the cluster was used. The Baum-Welch algorithm was used for the model parameter estimation (Baum and Petrie, 1966; Baum and Eagon, 1967; Baum and Sell, 1968; Baum et al., 1970; Baum, 1972). The Viterbi algorithm was used to estimate which state the molecule is in at each time (Viterbi, 1967). From this estimation, three mobility states were assigned along the trajectories. The obtained trajectories for each state were used to calculate the MSD,
(7)

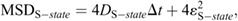
where *D*_S-*state*_ and *ε*_S-*state*_ are the diffusion coefficient and localization error for each diffusion state, respectively; the diffusion coefficients are represented as *D*_S-fast_, *D*_S-middle_ and *D*_S-slow_ for each state (Tables S3, S8). To obtain the steady state ratios of each state, the probabilities of each state at each time determined by the HMM were averaged for all trajectories and represented as p_S-fast_, p_S-middle_, and p_S-slow_ (Tables S3, S8).

To calculate the lifetimes of each state, the trajectories of each state were extracted by the Viterbi algorithm, as described above. The time duration of individual trajectories was collected and used to obtain the histogram in a cumulative form with *t*=0 as the time when the state started. Typically, the statistical distribution of the time duration exhibited an exponential-like decay curve. The state lifetime, *τ*, was calculated from the equation 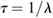 by fitting the histogram with the exponential function 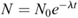 using the least-squares method and the obtained *λ*.

For the HMM, the localization error was not included in Eqn 6. Thus, *D*_HMM-*state*_ is the apparent diffusion coefficient for each state. After being assigned into the three diffusion states along the trajectories by the HMM, the obtained trajectories for each state were used to calculate the MSD using Eqn 7, from which we obtained *D*_S-*state*_ with the corresponding *ε*_S-*state*_ for each state. Considering that the fluorescent spots of single molecules are obtained by an accumulation of fluorescence over a period of 33 ms, it is reasonable that the error depends on the diffusion state. In fact, the localization errors differed depending on the different diffusion states, in which the fast diffusion state showed relatively large errors (∼90 nm), whereas slow diffusion showed conversely small errors (∼10 nm), as shown in Tables S3 and S5. When the localization error for each state was included as an unknown parameter in Eqn 6, the parameter estimation by the HMM did not work well.

In order to see whether the HMM can estimate the diffusion state along a trajectory for which the localization error of the fluorescent spots depends on the diffusion state, the particle trajectories generated by the numerical simulation were analyzed using the HMM (Fig. S8A–C). The particle trajectories were generated using the HMM model with *D*_S-*state*_ for each state with initial probabilities and a transition probability matrix A obtained from data for all protein species under control condition (Fig. S8A). A localization error was added to the coordinates of each particle in each frame based on *ε*_S-*state*_. The values of *ε*_S-*state*_ were 10, 12 and 88 nm for the slow, middle and fast states, respectively (Table S5). The obtained trajectories with the localization errors were analyzed by the HMM and MSD using Eqn 6 and Eqn 7. We confirmed that the HMM can estimate *D*_S-*state*_ with the corresponding *ε*_S-*state*_ (Fig. S8B).

To see whether the HMM can detect the state transition of diffusion, particle trajectories were generated by a numerical simulation with or without state transitions. Parameters obtained from the experimental data for all protein species under control condition were used for the simulation (Fig. S8D). The obtained particle trajectories were analyzed by the HMM, which revealed the presence and absence of state transitions (Fig. S8E).

### Bootstrap analysis for parameter estimation and AIC-based model selection

To calculate confidence intervals (c.i.) for the parameters estimated by the diffusion analysis, the bootstrap method was used (Efron, 1979). For the MSD and HMM analyses, 10 datasets were created by random sampling with overlap allowed from the original datasets of the single-molecule trajectories. The estimated parameters were obtained from the 10 datasets.

To calculate the AIC value, 10 datasets of displacement Δ*r* were created by random sampling with overlap from the original Δ*r* datasets. The diffusion parameters were estimated by MLE using Eqn 4, and log-likelihoods were obtained using Eqn 5 for the 10 datasets. From the calculated log likelihoods, AIC values were obtained (Fig. S2D). The relative likelihoods for the model with three versus two components were determined by calculating exp((AIC_3_−AIC_2_)/2).

For the diffusion analysis of the particle trajectory data obtained from the numerical simulation, 10 sets of 5000 trajectories from 100 frames were generated for each condition. The datasets were analyzed in the same manner as for the experimental data.

### Comparison with theoretical models

The Saffman–Delbrück model is expressed using the following equation (Saffman and Delbrück, 1975):
(8)

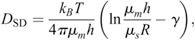
and the Stokes-Einstein-like model (Gambin et al., 2006) is expressed as:
(9)

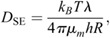
where *k_B_* is Boltzmann's constant (*k_B_*=1.38×10^−23^ m^2^ kg/s^2^ K), *T* is the absolute temperature, *μ_m_* is the viscosity of the cell membrane, *μ_s_* is the viscosity of the solvent surrounding the cell membrane, *h* is the thickness of the cell membrane, *R* is the radius of the membrane protein embedded in the cell membrane, *γ* is Euler's constant (*γ*≈0.5772), and *λ* is the characteristic length. *T* was set to *T*=294 K based on the room temperature at the time of the measurement, *μ_s_* was set to 9.6×10^−4^ Pa·s, and *h* was set to 3.8×10^−9^ m. The radius, *R*, of all membrane proteins with the same transmembrane number was assumed to be the same. The radii of DD3-3 for proteins with a single transmembrane and of car1 for proteins with seven transmembranes were used as representatives. *R* was estimated using PyMol with available 3D models, which were predicted using AlphaFold (Jumper et al., 2021; Varadi et al., 2021). If the region embedded in the cell membrane was oval, the average of the long and short radii was used. The fitting of each model to the measured data was performed using the least-squares method with *μ_m_* as a variable.

### Field preparation and particle simulation of diffusional motion

The trajectory data of all molecular species were analyzed together for each drug condition, and a field model was developed. The whole field was set as a square of size 10 μm×10 μm, which is equivalent to the size of one *Dictyostelium discoideum* cell. The field is divided into three regions. The analysis of the experimental data using the HMM concluded that the transition between fast and slow states was rare, and thus fast and slow regions did not adjoin each other. We randomly placed clumps of fast and slow squares in the middle field so that they were spaced apart from each other. By changing four parameters that include the size of the fast and slow clumps and their number (occupied percentage area), we prepared various fields. The following procedure was used to generate the trajectories of particles in the field: step 1, randomly select the position of the particle in the field; step 2, check the state of the position where the particle is located; step 3, generate movement of the particle with distance Δ*r* from the distribution of the diffusion coefficient that matches the state (Eqn 6). The values of *D*_S-*state*_, which were experimentally obtained, were used; step 4, select the direction of movement randomly; step 5, return to step 2 after the movement. After generating the trajectory of the particle, the error of the position corresponding to the state (*ε*_S-*state*_) was added to the position of the particle in each frame.

For particles leaving the field, we made the particles move to the position where they were reflected at the boundary of the field. However, the trajectory was broken at the reflected frame, and a new trajectory started as if another molecule had flowed in from outside.

The positions of individual particles were initially chosen randomly. During the simulation of the diffusion, the particles gradually accumulated in the slow squares due to the difference in diffusion coefficients in each region and then reached a steady state after a sufficient amount of time had passed. In this simulation, the trajectory of 100 frames from the beginning of the motion was deleted for the analysis of the particle diffusion. 5000 trajectories with 100 frames were generated for the diffusion analysis under each condition. The particle trajectories obtained by the simulation were analyzed in the same way as the single-molecule trajectories obtained by the experiments. Ten datasets were analyzed by the bootstrap method.

### Size and shapes of clump in the field model

Fields were prepared with various sizes of fast or slow clumps, and the obtained particle trajectories were analyzed by the HMM. The lifetimes of each state were calculated from the datasets of each state as described above. Because the lifetimes of fast and slow states under control condition were about 0.046±0.000 and 0.468±0.008 s (mean±95% c.i.), respectively (Table S5), the clump sizes selected for the field model covered a range of lifetimes (Fig. 4G).

A square clump was used for simplicity and ease of repeatability of the model calculation, but squares are thermodynamically unlike membrane microdomains. We therefore examined the effects of changing the shape of the clumps from squares to circles. Fields with one square or a circular clump in the middle region were prepared, and single-particle trajectories were generated in the fields under control condition. The square size was set as 50×50 and 250×250 nm^2^ for fast and slow clumps, respectively. The change in clump shape had no obvious effect on the particle mobility if the area was kept the same (Fig. S6). The squares in the field model shown in Fig. 4A and Fig. S5 can be replaced by circles, which more closely reflects the thermodynamics of lipid microdomains.

### Reproduction of tracking interruptions

From the HMM analysis, we estimated the ratio of each state in each frame. The results show that the proportion of faster states is high in the first frame, but as the frame progresses, the proportion of these states decreases rapidly to reach a steady state. This is due to an interruption in the single-molecule tracking – the tracking of molecules with fast diffusion motion is more likely to lose their tracking. When analyzing the trajectories, the fast state tends to be exposed at the start point by the interruption. The moment when molecules appeared was set to zero, and the start points of the trajectories were aligned. As a result, the ratio of faster states becomes higher in the first frame of the trajectories. In order to reproduce this phenomenon in the simulation, we included the interruption. When particle tracking is not interrupted, we set F, M and S to denote the number of trajectories that take the fast, middle and slow states at the start of the trajectories, respectively. When an interruption does occur, a trajectory is divided into two parts – the front part with the start point counted by the numbers F, M and S, and the back part with the new start point. f, m, and s, respectively, denoted the number of trajectories that take the fast, middle and slow states at the start of the trajectories with the newly created start point. The initial probability of each state (p_HMM_0_fast_, p_HMM_0_middle_, p_HMM_0_slow_) and the steady state probability of each state (p_S-fast_, p_S-middle_, p_S-slow_) are described as follows:
(10)

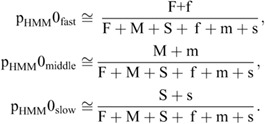
and
(11)

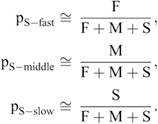
From the HHM of the experimental data, we can obtain the initial probability p_HMM_0_*state*_ and the steady state probability p_S-*state*_ of each state experimentally. From the HHM of the trajectory data simulated on a field without the interruption, we can obtain F, M and S for the various fields. By evaluating F, M and S using Eqn 11, we can select the field that reproduces the steady state ratio (p_S-fast_, p_S-middle_, p_S-slow_) of each mobility state. Then, the cutting points of the simulated trajectories were selected randomly along the trajectories, and we obtained f, m, and s for the interrupted trajectories. Because the initial probability of the slow state, p_HMM_0_slow_, was relatively small in the experimental data, we assumed that the interruption at the slow state is rare and set s=0. Using Eqn 10, we determined the number of cutting points that can reproduce the initial probabilities p_HMM_0_fast_ and p_HMM_0_middle_.

## Supplementary Material

10.1242/joces.260280_sup1 Supplementary information

## References

[JCS260280C1] Akaike, H. (1974). A new look at the statistical model identification. *IEEE Trans. Autom. Contr* 19, 716-723. 10.1109/TAC.1974.1100705

[JCS260280C2] Baum, L. E. and Eagon, J. A. (1967). An inequality with applications to statistical estimation for probabilistic functions of Markov processes and to a model for ecology. *Bull. Am. Math. Soc.* 73, 360-363. 10.1090/S0002-9904-1967-11751-8

[JCS260280C3] Baum, L. E. and Petrie, T. (1966). Statistical inference for probabilistic functions of finite state markov chains. *Ann. Math. Stat.* 37, 1554-1563. 10.1214/aoms/1177699147 PMC335547 16591502

[JCS260280C4] Baum, L. E. and Sell, G. R. (1968). Growth transformations for functions on manifolds. *Pac. J. Math.* 27, 211-227. 10.2140/pjm.1968.27.211

[JCS260280C5] Baum, L. E., Petrie, T., Soules, G. and Weiss, N. (1970). A maximization technique occurring in the statistical analysis of probabilistic functions of Markov Chains. *Ann. Math. Stat.* 41, 164-171. 10.1214/aoms/1177697196

[JCS260280C6] Baum, L. E. (1972). An inequality and associated maximization technique in statistical estimation of probabilistic functions of a markov process. *Inequalities* 3, 1-8.

[JCS260280C7] Clarke, D. T. and Martin-Fernandez, M. L. (2019). A brief history of single-particle tracking of the epidermal growth factor receptor. *Methods Protoc* 2, 12. 10.3390/mps2010012 31164594 PMC6481046

[JCS260280C8] Clarke, M., Köhler, J., Heuser, J. and Gerisch, G. (2002). Endosome fusion and microtubule–based dynamics in the early endocytic pathway of *Dictyostelium*. *Traffic* 3, 791-800. 10.1034/j.1600-0854.2002.31104.x 12383345

[JCS260280C9] de Keijzer, S., Galloway, J., Harms, G. S., Devreotes, P. N. and Iglesias, P. A. (2011). Disrupting microtubule network immobilizes amoeboid chemotactic receptor in the plasma membrane. *Biochim. Biophys. Acta* 1808, 1701-1708. 10.1016/j.bbamem.2011.02.009 21334306 PMC3079046

[JCS260280C10] Efron, B. (1979). Bootstrap methods: another look at the jackknife. *Ann. Stat* 7, 1-26. 10.1214/aos/1176344552

[JCS260280C11] Fujiwara, T., Ritchie, K., Murakoshi, H., Jacobson, K. and Kusumi, A. (2002). Phospholipids undergo hop diffusion in compartmentalized cell membrane. *J. Cell Biol.* 157, 1071-1082. 10.1083/jcb.200202050 12058021 PMC2174039

[JCS260280C12] Gambin, Y., Lopez-Esparza, R., Reffay, M., Sierecki, E., Gov, N. S., Genest, M., Hodges, R. S. and Urbach, W. (2006). Lateral mobility of proteins in liquid membranes revisited. *Proc. Natl. Acad. Sci. U.S.A* 103, 2098-2102. 10.1073/pnas.0511026103 16461891 PMC1413751

[JCS260280C13] Hiroshima, M., Pack, C. G., Kaizu, K., Takahashi, K., Ueda, M. and Sako, Y. (2018). Transient acceleration of epidermal growth factor receptor dynamics produces higher order signaling clusters. *J. Mol. Biol.* 430, 1381-1396. 10.1016/j.jmb.2018.02.018 29505756

[JCS260280C14] Jumper, J., Evans, R., Pritzel, A., Green, T., Figurnov, M., Ronneberger, O., Tunyasuvunakool, K., Bates, R., Žídek, A., Potapenko, A. et al. (2021). Highly accurate protein structure prediction with AlphaFold. *Nature* 596, 583-589. 10.1038/s41586-021-03819-2 34265844 PMC8371605

[JCS260280C15] Kashirina, A. S., López-Duarte, I., Kubánková, M., Gulin, A. A., Dudenkova, V. V., Rodimova, S. A., Torgomyan, H. G., Zagaynova, E. V., Meleshina, A. V. and Kuimova, M. K. (2020). Monitoring membrane viscosity in differentiating stem cells using BODIPY-based molecular rotors and FLIM. *Sci. Rep.* 10, 1-12. 10.1038/s41598-020-70972-5 32820221 PMC7441180

[JCS260280C16] Kamimura, Y., Miyanaga, Y. and Ueda, M. (2016). Heterotrimeric G-protein shuttling via Gip1 extends the dynamic range of eukaryotic chemotaxis. *Proc. Natl. Acad. Sci. U.S.A* 113, 4356-4361. 10.1073/pnas.1516767113 27044073 PMC4843477

[JCS260280C17] Kusumi, A., Nakada, C. and Fujiwara, T. (2005). Paradigm shift of the plasma membrane concept from the two-dimensional continuum fluid to the partitioned fluid: high-speed single-molecule tracking of membrane molecules. *Annu. Rev. Biophys. Biomol. Struct* 34, 351-378. 10.1146/annurev.biophys.34.040204.144637 15869394

[JCS260280C18] Kusumi, A., Tsunoyama, T. A., Hirosawa, K. M., Kasai, R. S. and Fujiwara, T. K. (2014). Tracking single molecules at work in living cells. *Nature Chem. Biol.* 10, 524-532. 10.1038/nchembio.1558 24937070

[JCS260280C19] Los, G. V., Encell, L. P., McDougall, M. G., Hartzell, D. D., Karassina, N., Zimprich, C., Wood, M. G., Learish, R., Ohana, R. F., Urh, M. et al. (2008). HaloTag: A novel protein labelling technology for cell imaging and protein analysis. *ACS Chem. Biol.* 3, 373-382. 10.1021/cb800025k 18533659

[JCS260280C20] Low-Nam, S. T., Lidke, K. A., Cutler, P. J., Roovers, R. C., van Bergen en Henegouwen, P. M. P., Wilson, B. S. and Lidke, D. S. (2011). ErbB1 dimerization is promoted by domain co-confinement and stabilized by ligand binding. *Nature Struct. Mol. Boil.* 18, 1244-1249. 10.1038/nsmb.2135 PMC3210321 22020299

[JCS260280C21] Mashanov, G. I., Nenasheva, T. A., Mashanova, A., Lape, R., Birdsall, N. J., Sivilotti, L. and Molloy, J. E. (2021). Heterogeneity of cell membrane structure studied by single molecule tracking. *Faraday Discuss.* 232, 358-374. 10.1039/D1FD00035G 34647559 PMC8704140

[JCS260280C22] Matsuoka, S., Iijima, M., Watanabe, T. M., Kuwayama, H., Yanagida, T., Devreotes, P. and Ueda, M. (2006). Single molecule analysis of chemoattractant-stimulated membrane recruitment of a PH domain-containing protein. *J. Cell Sci.* 119, 1071-1079. 10.1242/jcs.02824 16507590

[JCS260280C23] Matsuoka, S., Shibata, T. and Ueda, M. (2009). Statistical analysis of lateral diffusion and multistate kinetics in single-molecule imaging. *Biophys. J.* 97, 1115-1124. 10.1016/j.bpj.2009.06.007 19686659 PMC2726328

[JCS260280C24] Matsuoka, S., Miyanaga, Y. and Ueda, M. (2016). Multi-state transition kinetics of intracellular signaling molecules by single-molecule imaging analysis. *Methods Mol. Biol.* 1407, 361-379. 10.1007/978-1-4939-3480-5_25 27271914

[JCS260280C25] Miyanaga, Y., Matsuoka, S. and Ueda, M. (2009). Single-molecule imaging techniques to visualize chemotactic signaling events on the membrane of living *Dictyostelium* cells. *Methods Mol. Biol.* 571, 417-435. 10.1007/978-1-60761-198-1_28 19763983

[JCS260280C26] Miyanaga, Y., Kamimura, Y., Kuwayama, H., Devreotes, P. N. and Ueda, M. (2018). Chemoattractant receptors activate, recruit and capture G proteins for wide range chemotaxis. *Biochem. Biophys. Res. Commun.* 507, 304-310. 10.1016/j.bbrc.2018.11.029 30454895

[JCS260280C27] Murase, K., Fujiwara, T., Umemura, Y., Suzuki, K., Iino, R., Yamashita, H., Saito, M., Murakoshi, H., Ritchie, K. and Kusumi, A. (2004). Ultrafine membrane compartments for molecular diffusion as revealed by single molecule techniques. *Biophys. J* 86, 4075-4093. 10.1529/biophysj.103.035717 15189902 PMC1304307

[JCS260280C28] Pike, L. J. (2006). Rafts defined: a report on the Keystone symposium on lipid rafts and cell function. *J. Lipid Res.* 47, 1597-1598. 10.1194/jlr.E600002-JLR200 16645198

[JCS260280C50] Saffman, P. G. and Delbrück, M. (1975). Brownian motion in biological membranes. *Proc. Natl Acad. Sci. USA.* 72, 3111-3113. 10.1073/pnas.72.8.3111 1059096 PMC432930

[JCS260280C29] Saxton, M. J. and Jacobson, K. (1997). Single-particle tracking: applications to membrane dynamics. *Annu. Rev. Biophys. Biomol. Struct.* 26, 373-399. 10.1146/annurev.biophys.26.1.373 9241424

[JCS260280C30] Shu, S., Liu, X. and Korn, E. D. (2005). Blebbistatin and blebbistatin-inactivated myosin II inhibit myosin II-independent processes in *Dictyostelium*. *Proc. Natl. Acad. Sci. U.S.A* 102, 1472-1477. 10.1073/pnas.0409528102 15671182 PMC547870

[JCS260280C31] Simons, K. and Ikonen, E. (1997). Functional rafts in cell membranes. *Nature* 387, 569-572. 10.1038/42408 9177342

[JCS260280C32] Simons, K. and Toomre, D. (2000). Lipid rafts and signal transduction. *Nature Rev. Mol. Cell Biol.* 1, 31-39. 10.1038/35036052 11413487

[JCS260280C33] Singer, S. J. and Nicolson, G. L. (1972). The fluid mosaic model of the structure of cell membranes: Cell membranes are viewed as two-dimensional solutions of oriented globular proteins and lipids. *Science* 175, 720-731. 10.1126/science.175.4023.720 4333397

[JCS260280C34] Sugiyama, T., Pramanik, M. K. and Yumura, S. (2015). Microtubule-mediated inositol lipid signaling plays critical roles in regulation of blebbing. *PLoS ONE* 10, e0137032. 26317626 10.1371/journal.pone.0137032 PMC4552846

[JCS260280C35] Tang, L., Franca-Koh, J., Xiong, Y., Chen, M.-Y., Long, Y., Bickford, R. M., Knecht, D. A., Iglesias, P. A. and Devreotes, P. N. (2008). tsunami, the *Dictyostelium* homolog of the fused kinase, is required for polarization and chemotaxis. *Genes Dev.* 22, 2278-2290. 10.1101/gad.1694508 18708585 PMC2518819

[JCS260280C36] Tinevez, J. Y., Perry, N., Schindelin, J., Hoopes, G. M., Reynolds, G. D., Laplantine, E., Bednarek, S. Y., Shorte, S. L. and Eliceiri, K. W. (2017). TrackMate: An open and extensible platform for single-particle tracking. *Methods* 115, 80-90. 10.1016/j.ymeth.2016.09.016 27713081

[JCS260280C37] Ueda, M., Sako, Y., Tanaka, T., Devreotes, P. and Yanagida, T. (2001). Single-molecule analysis of chemotactic signaling in *Dictyostelium* cells. *Science* 294, 864-867. 10.1126/science.1063951 11679673

[JCS260280C38] UniProt Consortium (2021). UniProt: the universal protein knowledgebase in 2021. *Nucleic Acids Res.* 49, D480-D489. 10.1093/nar/gkaa1100 33237286 PMC7778908

[JCS260280C39] Varadi, M., Anyango, S., Deshpande, M., Nair, S., Natassia, C., Yordanova, G., Yuan, D., Stroe, O., Wood, G., Laydon, A. et al. (2021). AlphaFold protein structure database: massively expanding the structural coverage of protein-sequence space with high-accuracy models. *Nucleic Acids Res.* 50, D439-D444. 10.1093/nar/gkab1061 PMC8728224 34791371

[JCS260280C40] Viterbi, A. J. (1967). Error bounds for convolutional codes and an asymptotically optimum decoding algorithm. *IEEE Trans. Inf. Theory* 13, 260-269. 10.1109/TIT.1967.1054010

[JCS260280C41] Watts, D. J. and Ashworth, J. M. (1970). Growth of myxamoebae of the cellular slime mould *Dictyostelium discoideum* in axenic culture. *Biochem. J.* 119, 171-174. 10.1042/bj1190171 5530748 PMC1179339

[JCS260280C42] Yanagawa, M., Hiroshima, M., Togashi, Y., Abe, M., Yamashita, T., Shichida, Y., Murata, Y. M., Ueda, M. and Sako, Y. (2018). Single-molecule diffusion-based estimation of ligand effects on G protein-coupled receptors. *Sci. Signal* 11, eaao1917. 10.1126/scisignal.aao1917 30228224

[JCS260280C43] Yumura, S., Hashima, S. and Muranaka, S. (2014). Myosin II does not contribute to wound repair in *Dictyostelium* cells. *Biol. Open* 3, 966-973. 10.1242/bio.20149712 25238760 PMC4197445

