# Supplementary material for: Field model for multistate lateral diffusion of various transmembrane proteins observed in living Dictyostelium cells
Source: J Cell Sci. 2023 Feb 20;136(4):jcs260280. doi: 10.1242/jcs.260280 (PMC10022678; doi:10.1242/jcs.260280)
Supplement: Supplementary information [file joces-136-260280-s1.pdf]

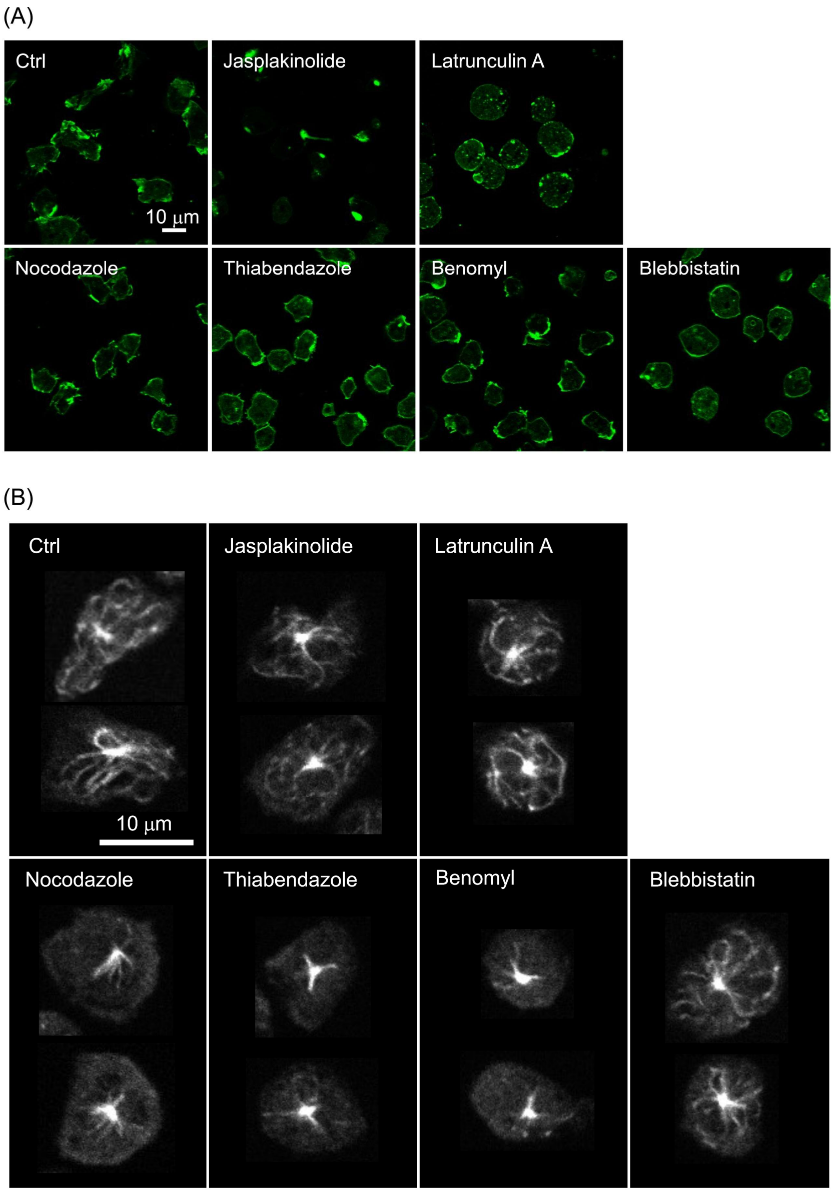

Fig. S1. Drug effects on F-actin and microtubules in *Dictyostelium* cells. (A) Effects of six drugs (jasplakinolide, latrunculin A, nocodazole, thiabendazole, benomyl and blebbistatin) on F-actin. The cells were stained with BODIPY-conjugated Phalloidin. Latrunculin A-treated cells exhibited punctuated staining of F-actin on the cell contour. Jasplakinolide-treated cells exhibited aggregates of F-actin, showing severe defects of the actin organization. There were no severe defects of F-actin observed in the nocodazole-, thiabendazole-, benomyl- and blebbistatin-treated cells. Scale bar, 10 µm. (B) Effects of the six drugs on microtubules. The microtubules were immuno-stained with 1:400 anti-α-tubulin antibody labeled with FITC (Sigma-Aldrich, F2168). The nocodazole-, thiabendazole- and benomyl-treated cells exhibited severe defects in microtubules, in which the microtubules remained only around the centrosome. There were no severe defects of microtubules observed in the jasplakinolide-, latrunculin A- and blebbistatin-treated cells. Scale bar, 10 µm.

Journal of Cell Science • Supplementary information

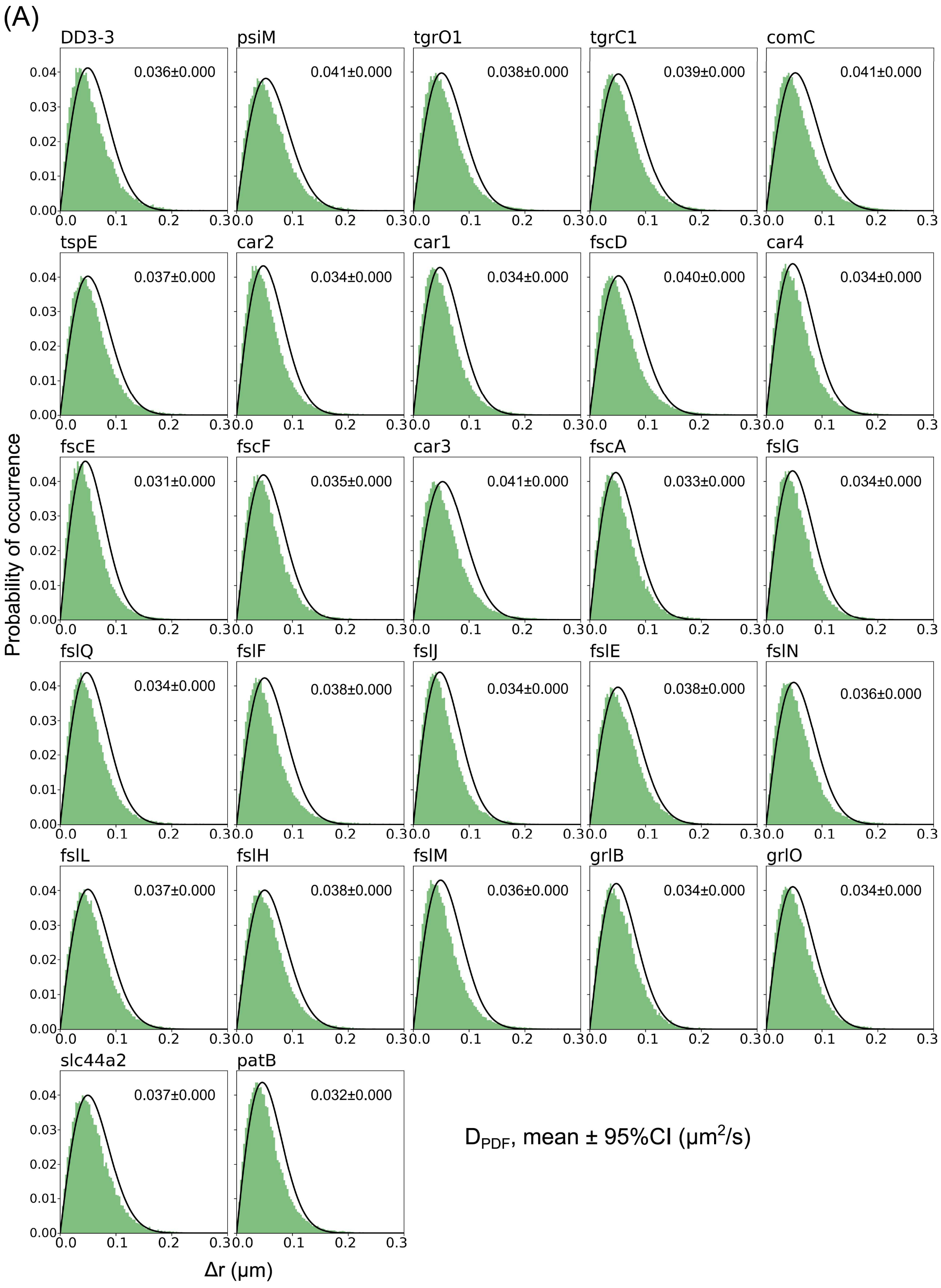
(A)
DD3-3
0.036±0.000
psiM
0.041±0.000
tgrO1
0.038±0.000
tgrC1
0.039±0.000
comC
0.041±0.000
tspE
0.037±0.000
car2
0.034±0.000
car1
0.034±0.000
fscD
0.040±0.000
car4
0.034±0.000
fscE
0.031±0.000
fscF
0.035±0.000
car3
0.041±0.000
fscA
0.033±0.000
fslG
0.034±0.000
fslQ
0.034±0.000
fslF
0.038±0.000
fslJ
0.034±0.000
fslE
0.038±0.000
fslN
0.036±0.000
fslL
0.037±0.000
fslH
0.038±0.000
fslM
0.036±0.000
grlB
0.034±0.000
grlO
0.034±0.000
slc44a2
0.037±0.000
patB
0.032±0.000
Probability of occurrence
Δr (μm)
D_{PDF}, mean ± 95%CI (μm²/s)

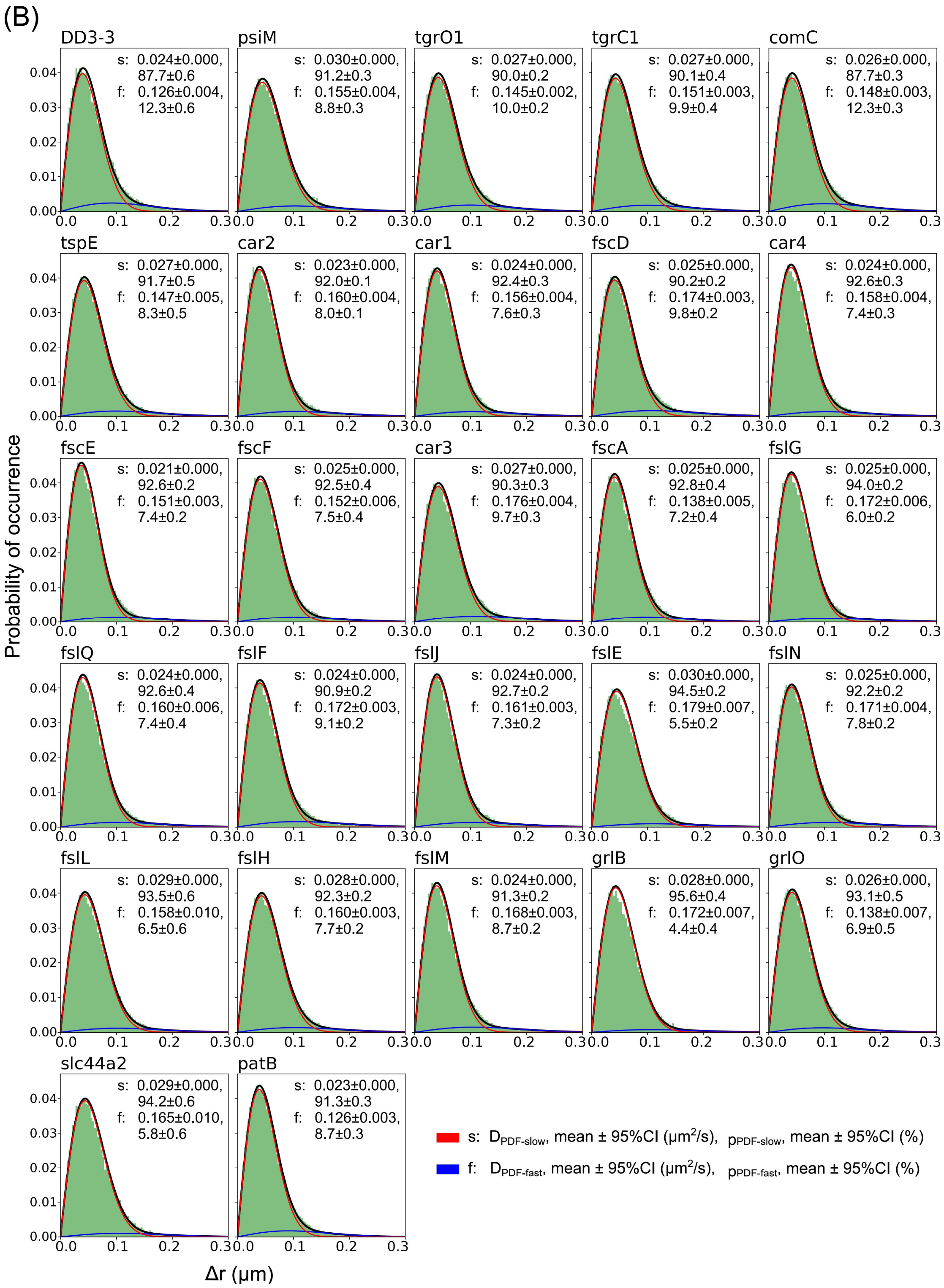
(B)
DD3-3
s: 0.024±0.000, 87.7±0.6
f: 0.126±0.004, 12.3±0.6
psiM
s: 0.030±0.000, 91.2±0.3
f: 0.155±0.004, 8.8±0.3
tgrO1
s: 0.027±0.000, 90.0±0.2
f: 0.145±0.002, 10.0±0.2
tgrC1
s: 0.027±0.000, 90.1±0.4
f: 0.151±0.003, 9.9±0.4
comC
s: 0.026±0.000, 87.7±0.3
f: 0.148±0.003, 12.3±0.3
tspE
s: 0.027±0.000, 91.7±0.5
f: 0.147±0.005, 8.3±0.5
car2
s: 0.023±0.000, 92.0±0.1
f: 0.160±0.004, 8.0±0.1
car1
s: 0.024±0.000, 92.4±0.3
f: 0.156±0.004, 7.6±0.3
fscD
s: 0.025±0.000, 90.2±0.2
f: 0.174±0.003, 9.8±0.2
car4
s: 0.024±0.000, 92.6±0.3
f: 0.158±0.004, 7.4±0.3
fscE
s: 0.021±0.000, 92.6±0.2
f: 0.151±0.003, 7.4±0.2
fscF
s: 0.025±0.000, 92.5±0.4
f: 0.152±0.006, 7.5±0.4
car3
s: 0.027±0.000, 90.3±0.3
f: 0.176±0.004, 9.7±0.3
fscA
s: 0.025±0.000, 92.8±0.4
f: 0.138±0.005, 7.2±0.4
fslG
s: 0.025±0.000, 94.0±0.2
f: 0.172±0.006, 6.0±0.2
fslQ
s: 0.024±0.000, 92.6±0.4
f: 0.160±0.006, 7.4±0.4
fslF
s: 0.024±0.000, 90.9±0.2
f: 0.172±0.003, 9.1±0.2
fslJ
s: 0.024±0.000, 92.7±0.2
f: 0.161±0.003, 7.3±0.2
fslE
s: 0.030±0.000, 94.5±0.2
f: 0.179±0.007, 5.5±0.2
fslN
s: 0.025±0.000, 92.2±0.2
f: 0.171±0.004, 7.8±0.2
fslL
s: 0.029±0.000, 93.5±0.6
f: 0.158±0.010, 6.5±0.6
fslH
s: 0.028±0.000, 92.3±0.2
f: 0.160±0.003, 7.7±0.2
fslM
s: 0.024±0.000, 91.3±0.2
f: 0.168±0.003, 8.7±0.2
grlB
s: 0.028±0.000, 95.6±0.4
f: 0.172±0.007, 4.4±0.4
grlO
s: 0.026±0.000, 93.1±0.5
f: 0.138±0.007, 6.9±0.5
slc44a2
s: 0.029±0.000, 94.2±0.6
f: 0.165±0.010, 5.8±0.6
patB
s: 0.023±0.000, 91.3±0.3
f: 0.126±0.003, 8.7±0.3
Probability of occurrence
Δr (μm)
s: D_PDF-slow, mean ± 95%CI (μm²/s), p_PDF-slow, mean ± 95%CI (%)
f: D_PDF-fast, mean ± 95%CI (μm²/s), p_PDF-fast, mean ± 95%CI (%)

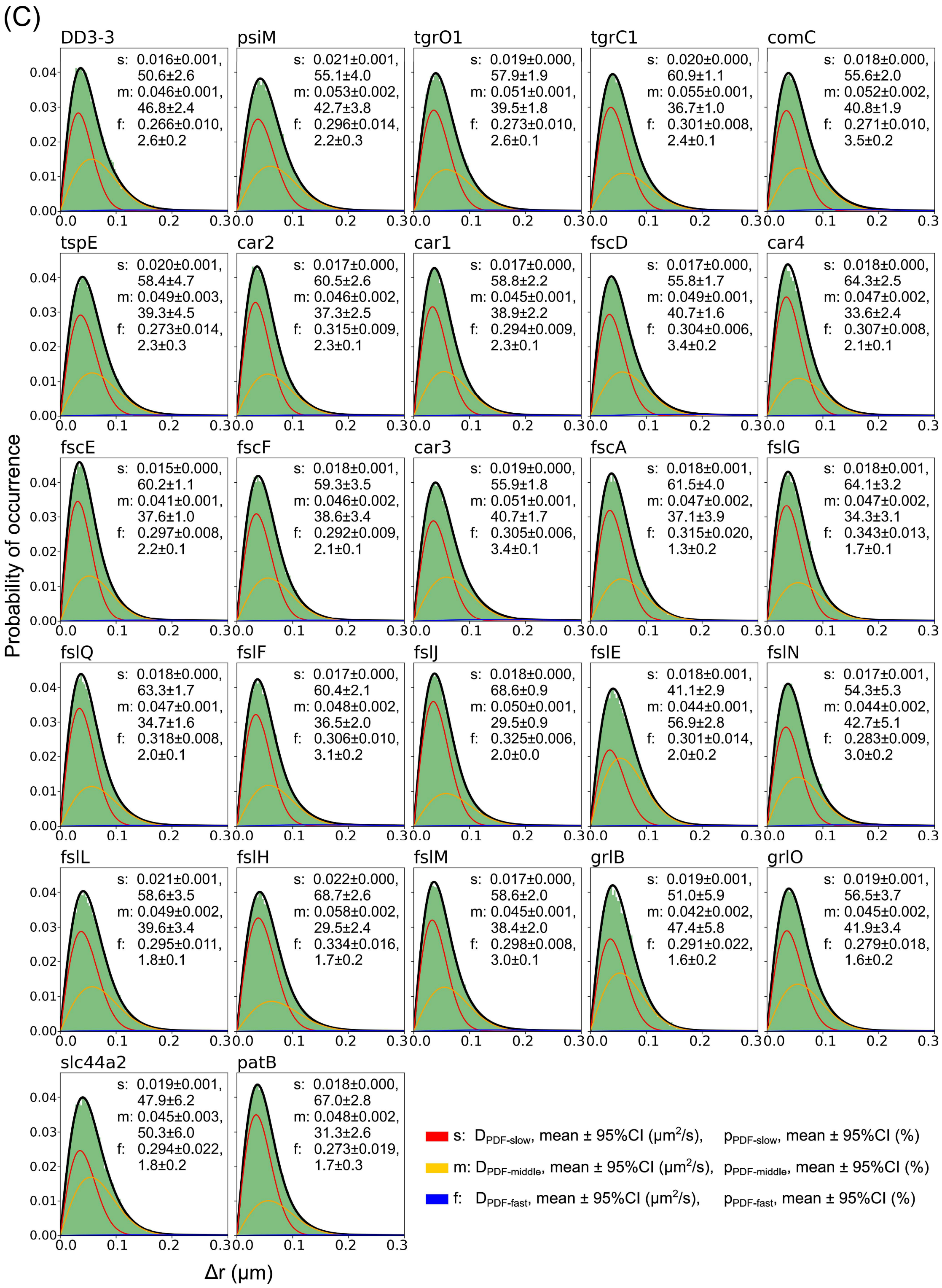

Journal of Cell Science • Supplementary information

(D)

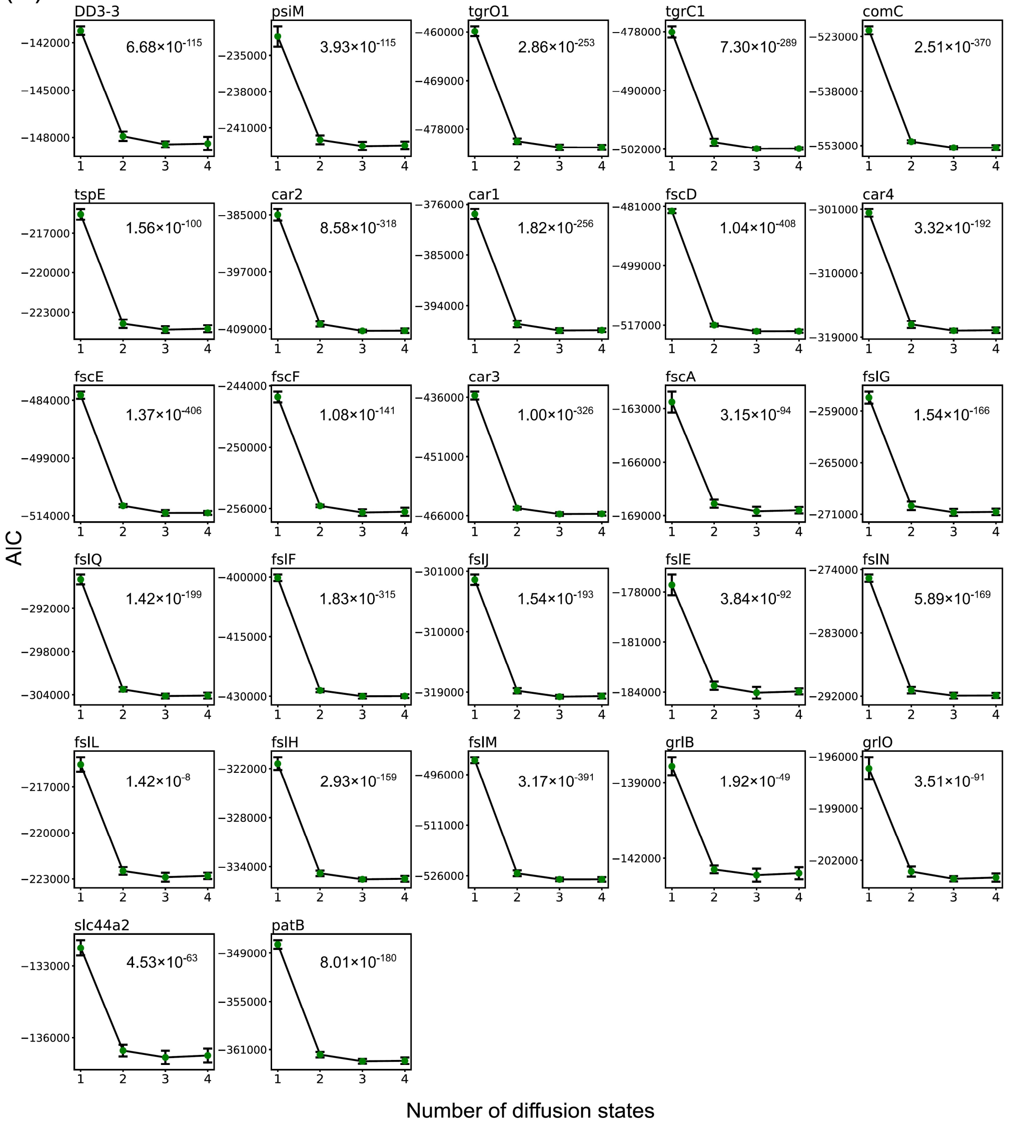

Fig. S2. Histogram of displacements (Δr) at 33-msec intervals of 27 different transmembrane proteins and AIC values for the 27 transmembrane proteins. Histograms of the displacements (green) are overlaid with the PDF of the single diffusion state (A), the two diffusion states (B) and the three diffusion states (C) (Eq. (3)) (black). The colored lines indicate the PDFs corresponding to each diffuse state: fast (blue), middle (yellow), slow (red). $D_{PDF\text{-}fast}$, $p_{PDF\text{-}fast}$, $D_{PDF\text{-}middle}$, $p_{PDF\text{-}muddle}$, $D_{PDF\text{-}slow}$ and $p_{PDF\text{-}slow}$ were obtained using the MLE from the Δr datasets. The numbers in each figure are the diffusion coefficient $D_{PDF\text{-}\mathit{state}}$ (top) and ratio $p_{PDF\text{-}\mathit{state}}$ (bottom) of the fast (f), middle (m) and slow (s) states (mean±95% CI). The bin size for the histograms is 3 nm. (D) The AIC values (mean±95% CI) were calculated using Eq. (5) for N = 1 to 4, where N is the number of diffusion states. The relative likelihood of the three-component model relative to the two-component model, exp((AIC3 – AIC2)/2), was calculated and is shown in each figure.

Journal of Cell Science • Supplementary information

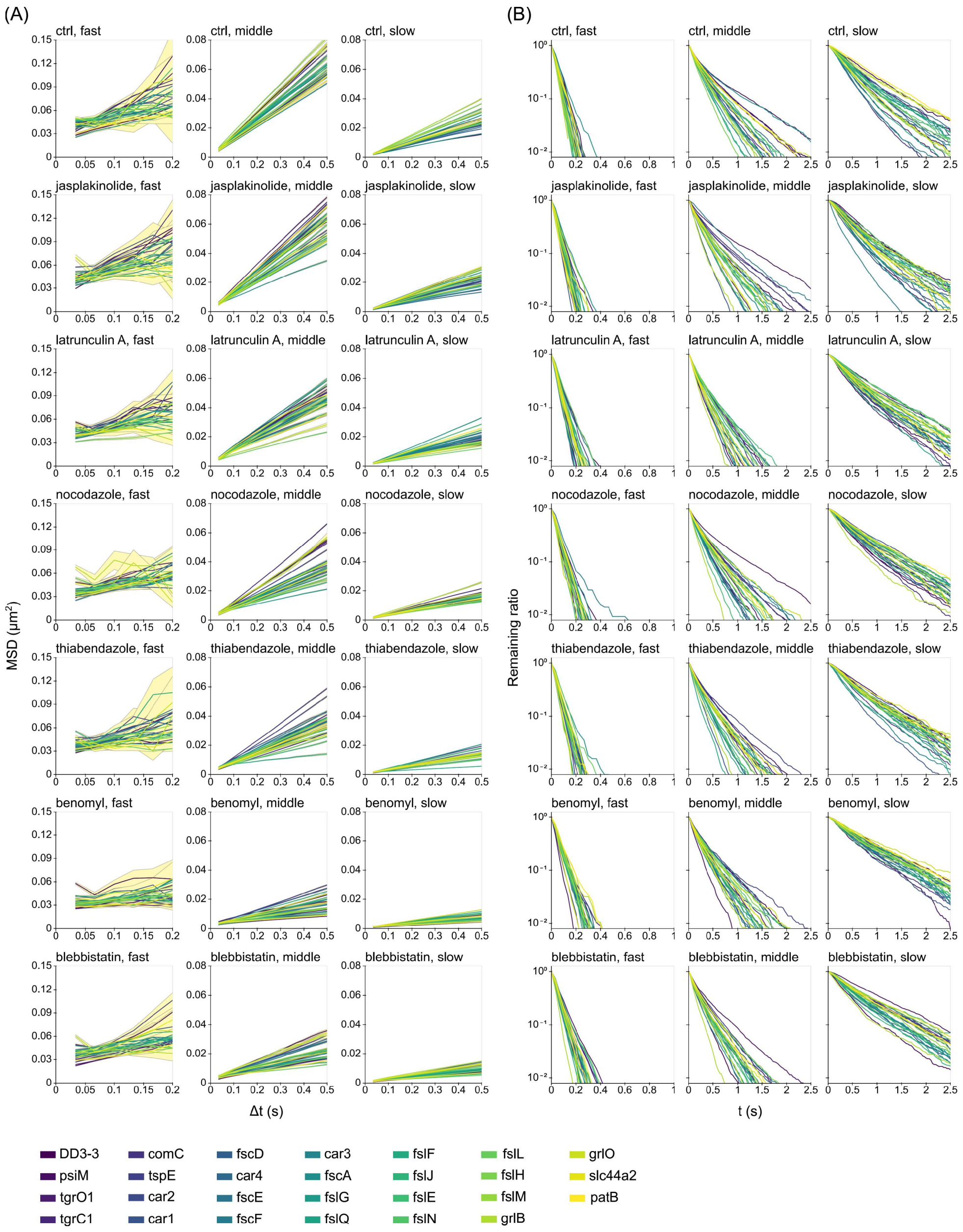

Fig. S3. MSDs and lifetimes of three diffusion states under drug-treated conditions. The mobilities of the 27 transmembrane proteins were observed under the control and six drug conditions. Using the HMM, the mobilities were separated into three states, from which the MSDs and lifetimes were calculated. The data of the 27 transmembrane proteins are represented in different colors. (A) MSDs of fast, middle and slow states. Error bars represent SE. The diffusion coefficients ($D_{S\text{-}state}$) and the corresponding localization errors ($\varepsilon_{S\text{-}state}$) of each diffusion state were estimated using Eq. (7). The estimated values are shown in Table S3. (B) Lifetimes of fast, middle and slow states. The lifetime values of each diffusion state are shown in Table S4. The total average lifetimes of the 27 proteins under drug-treated conditions are shown in Table S5.

Journal of Cell Science • Supplementary information

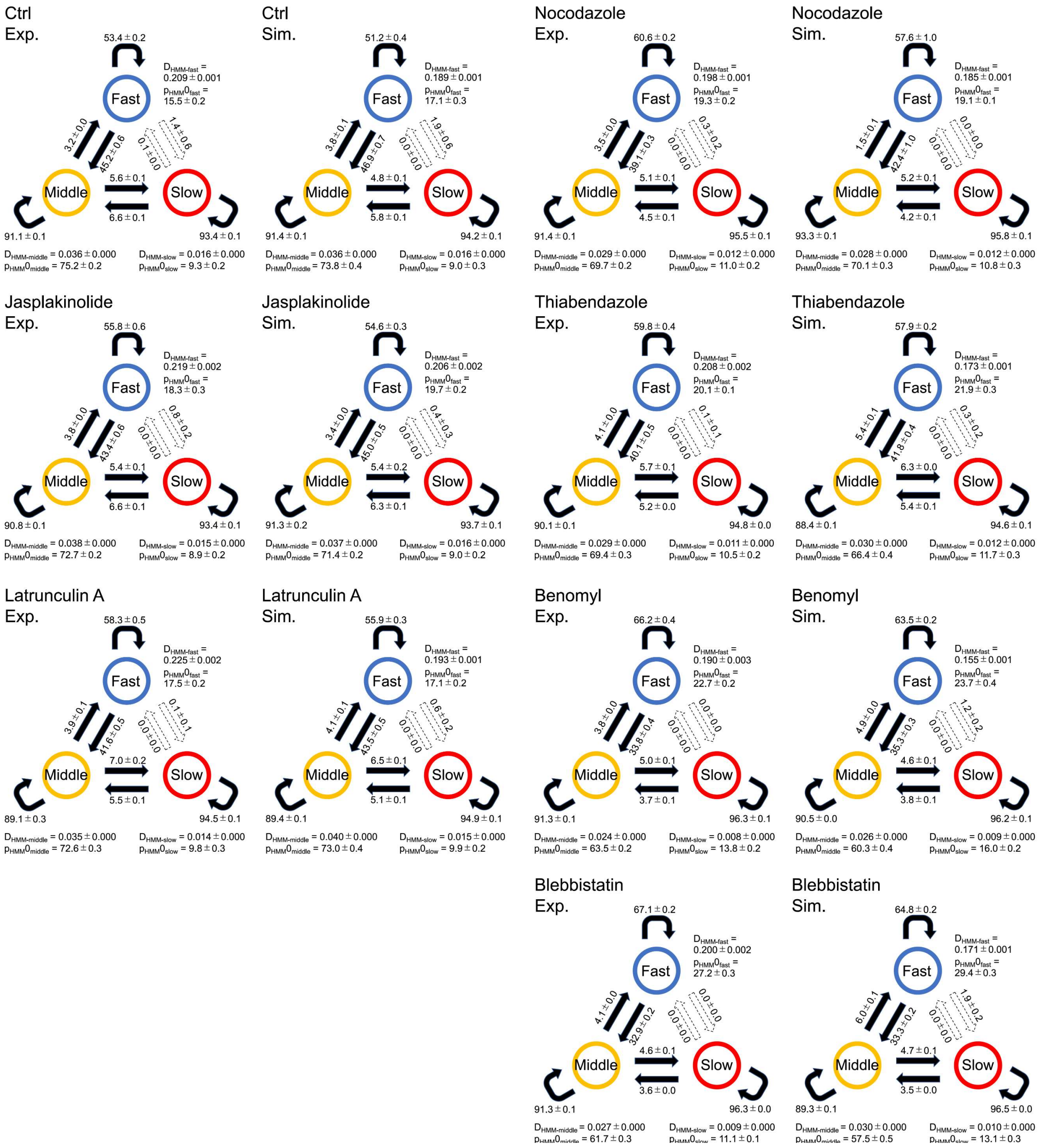

Fig. S4. Hidden Markov models from experimental and simulated data. Schematic diagrams of the HMMs and parameters of the models obtained from simulations and experiments in the control and six drug conditions. The values shown next to the arrows represent the state transition probabilities of the models. $D_{HMM\text{-}state}$ and $p_{HMM}0_{state}$ represent the diffusion coefficients and the initial probabilities of the hidden Markov models corresponding to the three states, respectively. The particle trajectories obtained by the simulation were analyzed in the same manner as the experimentally-obtained single-molecule trajectories by the HMM analysis. Values in this figure are also shown in Table S6.

Journal of Cell Science • Supplementary information

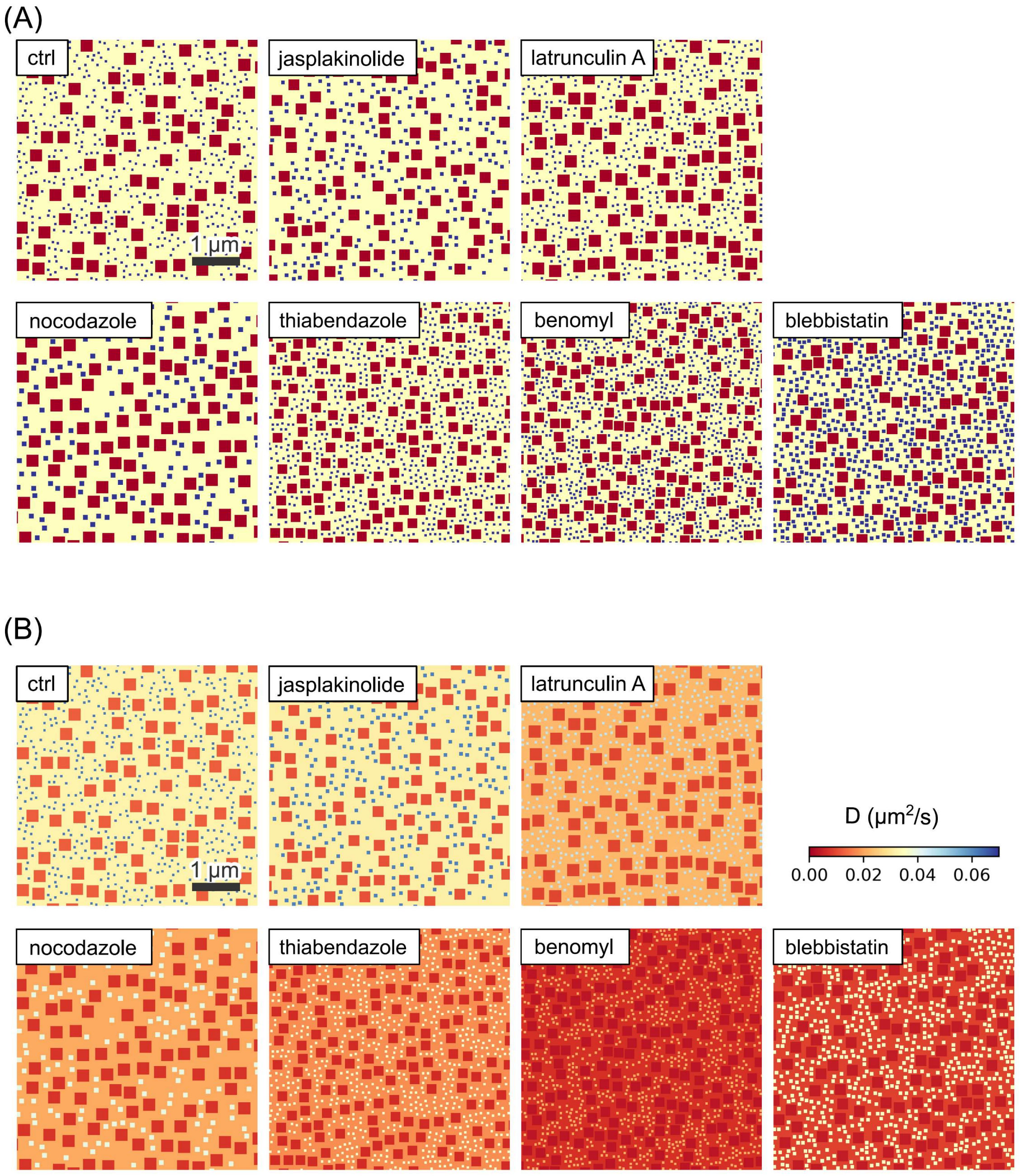

Fig. S5. Field models with three distinct regions corresponding to three diffusion states. (A) Field models for the lateral diffusion of the transmembrane proteins under control and drug-treated conditions. The fast, middle, and slow states are represented by the blue, yellow, and red areas, respectively. (B) The fields redisplayed as heat maps of the magnitudes of the diffusion coefficients for each region. The diffusion properties and field sizes in each drug condition for the simulation are shown in Table S5, S6. The size of the fields is 5 µm × 5 µm. Scale bar, 1 µm.

Journal of Cell Science • Supplementary information

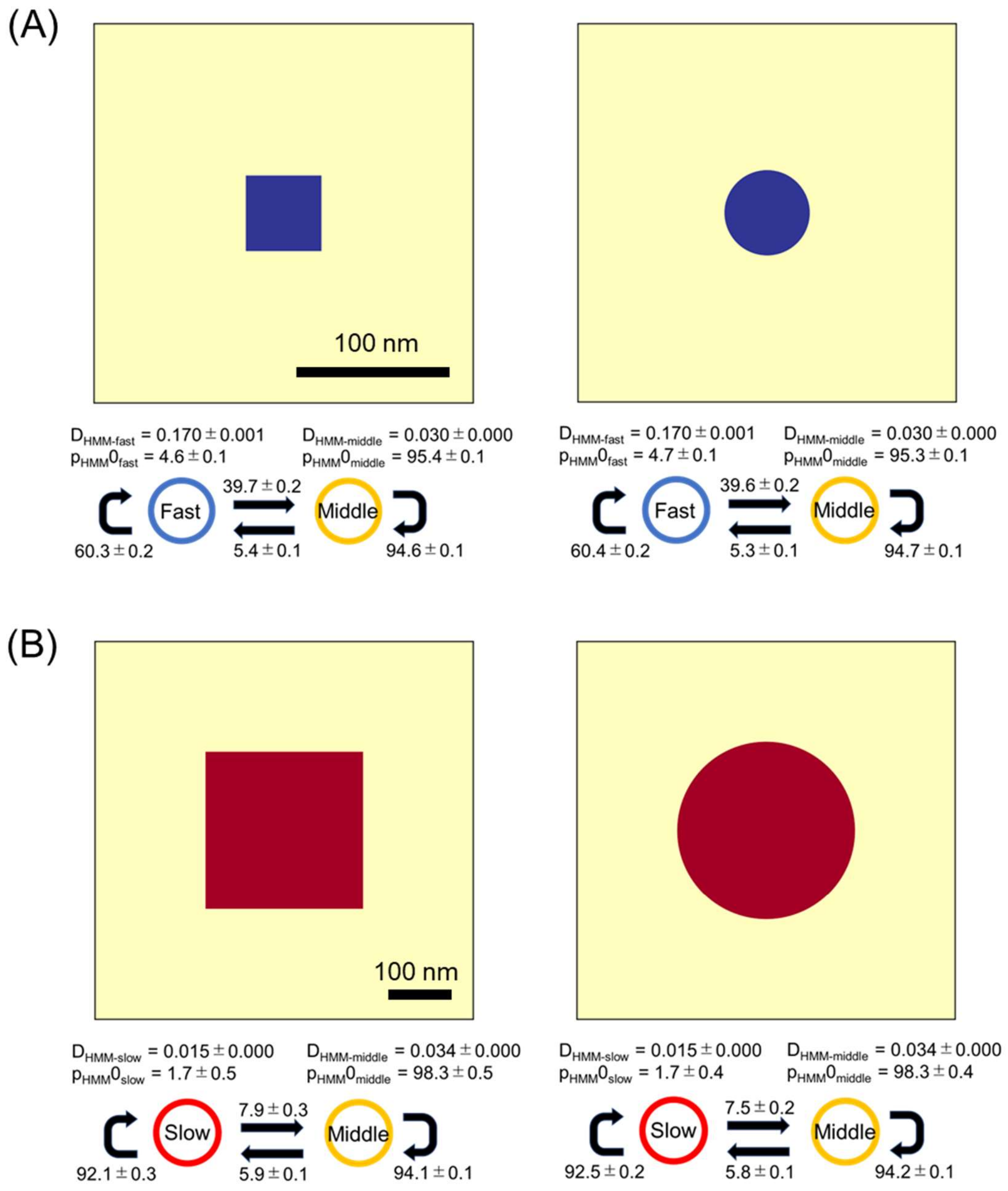

Fig. S6. Effects of clump shape on mobility properties. Fields with one fast clump (A) and one slow clump (B) in the middle region were prepared, and single-particle trajectories were generated on the fields under control condition. The obtained trajectories were analyzed in the same manner as the single-molecule trajectories by the HMM. To examine the effects of the clump shape on the particle mobility, square and circular clumps of the same size were prepared (50 and 250 nm for fast and slow clumps, respectively). The size had no obvious effect on the mobility. Thus, the squares in the field models shown in Fig. 4A and Fig. S5 can be replaced with circles, which is more representative of the thermodynamics of lipid microdomains.

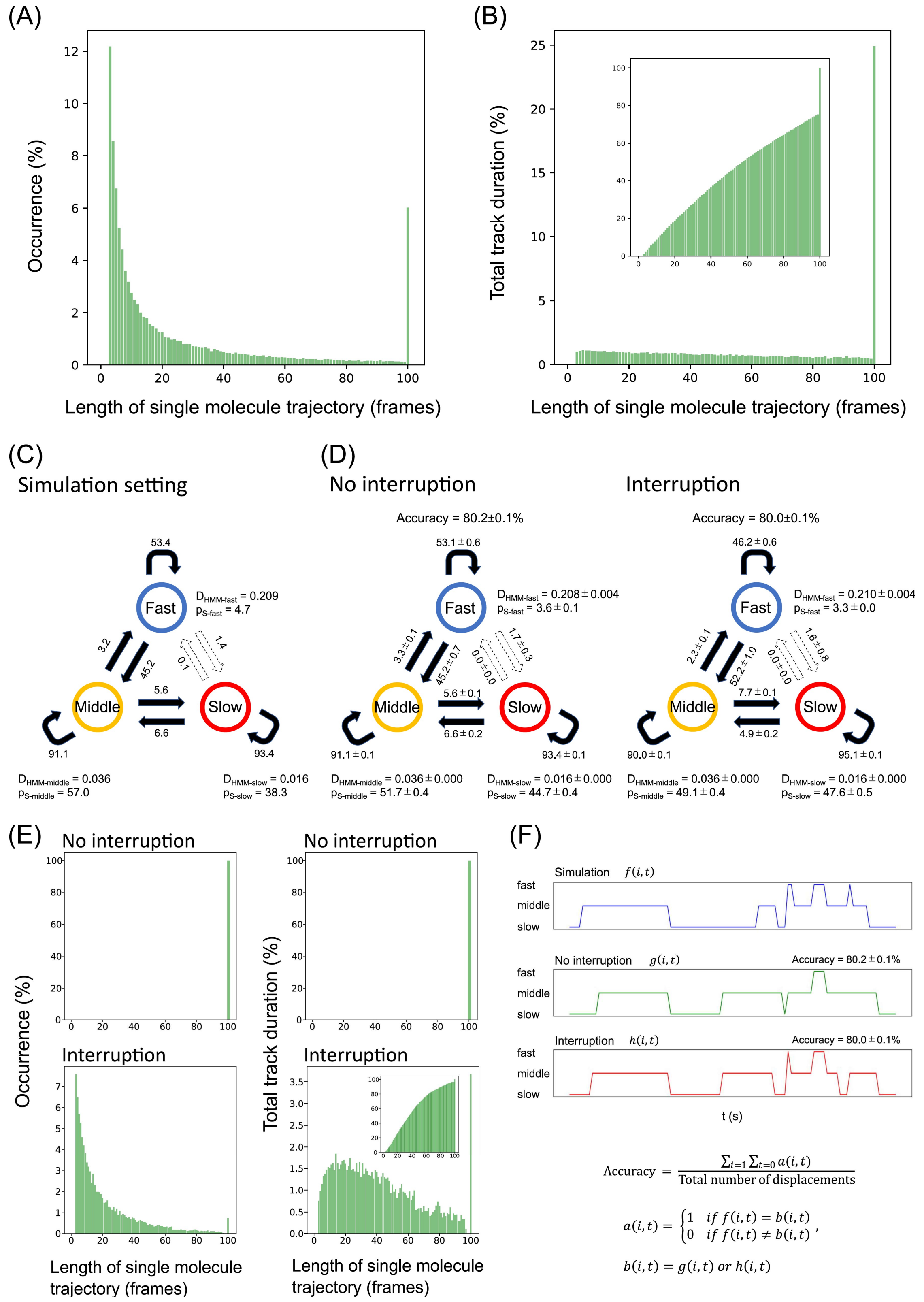

Journal of Cell Science • Supplementary information

Fig. S7. Trajectory length obtained by single-molecule tracking and effects of trajectory interruption on the estimation of diffusion properties using the HMM. (A) The occurrence of each length in the single-molecule trajectories obtained experimentally for the 27 membrane proteins. Interruption in the single-molecule tracking resulted in trajectories shorter than 100 frames. The mean and deviation of the tracking were 23.9±28.9 frames (mean±SD). (B) Percentage of each trajectory length. **Inset**, Culminated percentage of each trajectory length. Durations longer than 1 second (30 frames) constitute more than 70%. (C) Particle diffusion with three states for the numerical simulation based on the HMM. (D) The estimated parameters with and without trajectory interruption. (E) The occurrence of each particle trajectory length generated by the numerical simulation. With trajectory interruption, the trajectories were shortened by interrupting 100-frame long trajectories. The track duration was 21.0±20.1 frames (mean±SD). **Inset**, Culminated percentage of trajectory lengths. The simulations were designed to have more truncations than in the experimental data. (F) Definition of the coincidence between the simulated diffusion state and the estimated diffusion state. To evaluate the degree of coincidence between the numerically generated trajectory and HMM, we defined accuracy as follows. The state of the i-th trajectory at frame t of the generated trajectory is denoted as $f(i, t)$, the state estimated by the HMM of the full-length trajectory is denoted as $g(i, t)$, and the state estimated by the HMM of the interrupted trajectory is denoted as $h(i, t)$. Using $a(i, t)$, the accuracy rate can be expressed as the “accuracy”.

Journal of Cell Science • Supplementary information

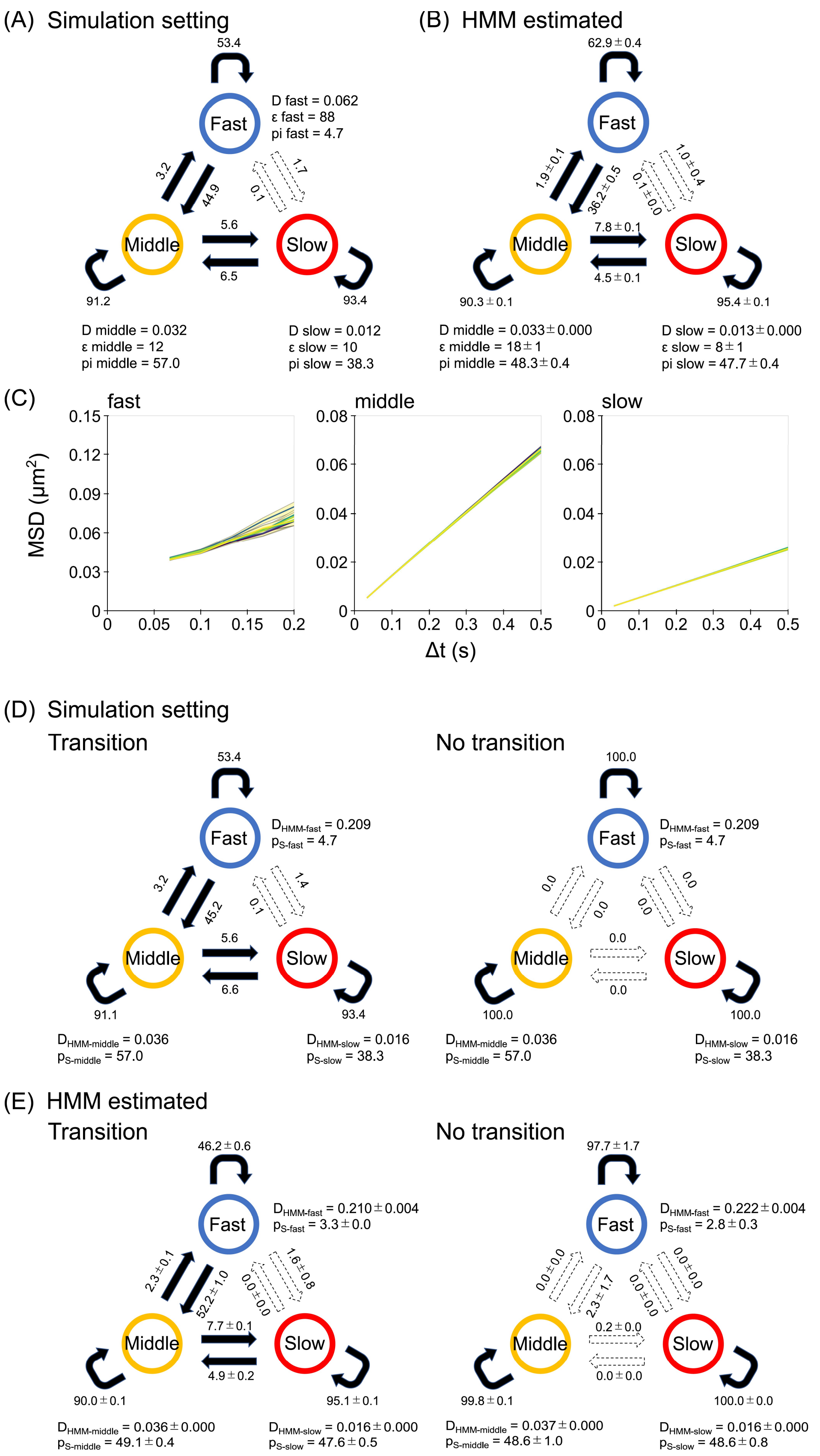

(A) Simulation setting
53.4
Fast
D fast = 0.062
ε fast = 88
pi fast = 4.7
3.2
44.9
0.1
1.7
Middle
5.6
6.5
Slow
91.2
93.4
D middle = 0.032
ε middle = 12
pi middle = 57.0
D slow = 0.012
ε slow = 10
pi slow = 38.3
(B) HMM estimated
62.9±0.4
Fast
1.9±0.1
36.2±0.5
0.1±0.0
1.0±0.4
Middle
7.8±0.1
4.5±0.1
Slow
90.3±0.1
95.4±0.1
D middle = 0.033±0.000
ε middle = 18±1
pi middle = 48.3±0.4
D slow = 0.013±0.000
ε slow = 8±1
pi slow = 47.7±0.4
(C)
fast
middle
slow
MSD (μm²)
Δt (s)
(D) Simulation setting
Transition
53.4
Fast
DHMM-fast = 0.209
pS-fast = 4.7
3.2
45.2
0.1
1.4
Middle
5.6
6.6
Slow
91.1
93.4
DHMM-middle = 0.036
pS-middle = 57.0
DHMM-slow = 0.016
pS-slow = 38.3
No transition
100.0
Fast
DHMM-fast = 0.209
pS-fast = 4.7
0.0
0.0
0.0
0.0
Middle
0.0
0.0
Slow
100.0
100.0
DHMM-middle = 0.036
pS-middle = 57.0
DHMM-slow = 0.016
pS-slow = 38.3
(E) HMM estimated
Transition
46.2±0.6
Fast
DHMM-fast = 0.210±0.004
pS-fast = 3.3±0.0
2.3±0.1
52.2±1.0
0.0±0.0
1.6±0.8
Middle
7.7±0.1
4.9±0.2
Slow
90.0±0.1
95.1±0.1
DHMM-middle = 0.036±0.000
pS-middle = 49.1±0.4
DHMM-slow = 0.016±0.000
pS-slow = 47.6±0.5
No transition
97.7±1.7
Fast
DHMM-fast = 0.222±0.004
pS-fast = 2.8±0.3
0.0±0.0
2.3±1.7
0.0±0.0
0.0±0.0
Middle
0.2±0.0
0.0±0.0
Slow
99.8±0.1
100.0±0.0
DHMM-middle = 0.037±0.000
pS-middle = 48.6±1.0
DHMM-slow = 0.016±0.000
pS-slow = 48.6±0.8

Fig. S8. Estimation of localization errors and state transitions of single-molecule trajectories by the HMM. Simulation with localization errors (A)-(C) and with/without state transitions (D)(E). (A) Parameters to generate particle trajectories by numerical simulations using the HMM. (B) Parameters estimated by analyzing the simulation trajectories using the HMM. (C) MSDs of fast, middle, and slow states estimated using the HMM. For the bootstrap analysis, 10 datasets of 5000 trajectories from 100 frames were generated. The estimated parameters are shown as means with 95% confidence intervals (CI) obtained from the 10 sets. (D) Parameters to generate particle trajectories by numerical simulations using the HMM. The parameter values are the same as those obtained from the experimental data of the single-molecule trajectories for all protein species combined under the control condition (see Fig. S4, Ctrl Exp.). The trajectories were generated by numerical simulation with (left) and without (right) state transitions. (E) Estimated mobility parameters by the HMM from the trajectories obtained by the simulation with (left) and without (right) state transitions. The HMM detected the presence or absence of state transitions and estimated the parameters successfully.

Table S1. List of all transmembrane proteins and primers used in this study.

Gene	UniplotKB accession number	Number of transmembrane α-helix	Length	Mass (Da)	Vector	Protein tag	Forward primer's sequence	Reverse primer's sequence
DD3-3	Q58A42	1	1851	69,498	pHK12 (neo)	HaloTag2	ATGAGATTTTTATCAAGTTTAATTATTT	TTAAACTAATCTCTTGAGACCTGGTACTTTTGAAAC
psiM	Q54P69	1	2718	79,232	pHK12 (neo)	HaloTag2	ATGAAAAAAATAAACAATAATAAAATTT	TTATGTTGGTGAGTTATAGAGTGGATTTTCACCTCC
tgrO1	Q55GI8	1	887	99,445	pHK12 (neo)	HaloTag7	ATGGAGAAAAAATTACTAATAATAGTT	TTATTGTTTCATTAATTCTTTCTCTTTTGATGA
tgrC1	P42523	1	889	97,443	pHK12 (neo)	HaloTag7	ATGGAAAAGAAAATAATATTACTGATT	TTAAAATTTTTTACCTATAAATTTTTTCACACG
comC	Q55AP8	1	1501	162,794	pHK12 (neo)	HaloTag7	ATGATTAAAAAATATTTATTTTTATTT	TTATGATTGTGTATTACCACCATCACTACCACT
tspE	Q54F08	4	778	26,527	pHK12 (neo)	HaloTag2	ATGACATTTGTGGATAATTTTGAATTC	TTAATCTTGTAAAATAAATGATTTTGATTTTAC
car2	P34907	7	1228	43,301	pHK12 (neo)	HaloTag2	ATGACTATTATGTCAGATATTATCGCAC	TTAAATCATATCATTTTTATTATTTATAT
car1	P13773	7	1322	44,312	pHK12 (neo)	HaloTag2	ATGGGTCTTTTAGATGGAAATCCAGCC	TCAATTATTTCCTTGACCATTTGTTGAAG
fscD	Q75JP9	7	1506	50,663	pHK12 (neo)	HaloTag2	ATGTTTATTATCCTAAAATTTTTAATATC	TTAATTTTTTGAATCTACTCCTGATAAC
car4	Q9TX43	7	1427	51,467	pHK12 (neo)	HaloTag2	ATGAAAGTATTACAAGAGATTAATCTTAC	TCAAAACTTATTATCTTTTTTTTCTC
fscE	Q559M5	7	1535	50,531	pHK12 (neo)	HaloTag2	ATGATATCTCATATTAAAAAATTTATAAA	TTATGGATCATGTTTAGAATCATCAATAC
fscF	Q1ZXB0	7	1694	52,173	pHK12 (neo)	HaloTag2	ATGATTTTTAATAATTTAAAAAATC	TTAAATTTCATCTACACTATTTAATGAAG
car3	P35352	7	1638	56,161	pHK12 (neo)	HaloTag2	ATGGAAAATTTAAATACAACAAGTACGGC	TTAAACATTTGAATCTTTTTTTTTGTTAC
fscA	Q54H37	7	1940	63,275	pHK12 (neo)	HaloTag2	ATGAAATTTAATTTTAAATTAATTTTAATA	TTAATTATTTTCATCATTATTGCTATTATT
fslG	Q54J71	7	1806	64,284	pHK12 (neo)	HaloTag2	ATGAAATCAATAATATTTATAACTTTTTTT	TTAAATTTTTTCCAGTTTTATTTCATTTTG
fslQ	Q1ZXE4	7	2019	65,439	pHK12 (neo)	HaloTag2	ATGAAAAAATTCATTTTTAATAAATATTTTA	TTATGGTTGTTGATTTATTTCAGTAGAC
fslF	Q54J77	7	1857	66,711	pHK12 (neo)	HaloTag2	ATGAAAATTTTAATAATTTTTATTATTTTT	CTAATTAATTTTAATTGATTCTAATTCAAC
fslJ	Q556J4	7	2538	68,945	pHK12 (neo)	HaloTag2	ATGGTCTCAAATAAAAATCTTTTACC	TTATAGAGCATCAGAATTTGAATCTAATTC
fslE	Q54J78	7	2225	69,006	pHK12 (neo)	HaloTag2	ATGGAAATGATTAGAATTTTTTTAATTTAT	TTATTTTTTTTCAAGTTCAATAGATTTAC
fslN	Q55CY2	7	2094	69,796	pHK12 (neo)	HaloTag2	ATGAAAATGAAAATGAAAATTCTTTTAATT	TTAAGCTTGAGATTGTTTATCATCATTATT
fslL	Q54PF8	7	2177	70,185	pHK12 (neo)	HaloTag2	ATGATTACAAATAAATCAAAGTACTATTTT	TTAAGATTTTGAAACTTCGATATCTTTTAA
fslH	Q556C6	7	2183	69,274	pHK12 (neo)	HaloTag2	ATGAATCTGAAATTTTATAACTTAATTTTT	TTAAAGATCATCATCATCAGTATTATAATT
fslM	Q556N7	7	2183	70,199	pHK12 (neo)	HaloTag2	ATGAAATCAATATTTATTATTATTTTTATT	TTATTTTATATTAACAGAACCAATTTC
grlB	Q86HH3	7	2344	84,118	pHK12 (neo)	HaloTag2	ATGAAAAATTTAATTTCAATTATTCTTTTA	TTAAAGGTTATTAGAATCAATTTCAACTTC
grlO	Q55AP3	7	2699	92,649	pHK12 (neo)	HaloTag2	ATGAAAAAAGTGTTTTTTTTGATTTTAATT	TCAAGTATCTGAAATAATTTCATTTTC
slc44a2	Q54I48	9	2096	71,809	pHK12 (neo)	HaloTag2	ATGAGTTCAGAGGATTTACAGGATCACC	TTAACAACATTTTGAACGACCTTTTCC
patB	P54679	10	1058	117,268	pHK12 (neo)	HaloTag7	ATGGATAATAATCAAATTCCAAAAAAT	TTAAACTTTATTATCGGTAACAACAGATTTATG

Table S2. Diffusion coefficient D_M estimated from the MSD of the 27 membrane proteins in each drug condition.

Gene	Ctrl		Jasplakinolide		Latrunculin A		Nocodazole		Thiabendazole		Benomyl		Blebbistatin	
	D_M (μm²/s)	ε_M (nm)	D_M (μm²/s)	ε_M (nm)	D_M (μm²/s)	ε_M (nm)	D_M (μm²/s)	ε_M (nm)	D_M (μm²/s)	ε_M (nm)	D_M (μm²/s)	ε_M (nm)	D_M (μm²/s)	ε_M (nm)
DD3-3	0.020±0.001	27±1	0.024±0.000	29±1	0.014±0.001	38±1	0.016±0.000	31±0	0.011±0.000	32±1	0.006±0.000	39±1	0.010±0.000	36±1
psiM	0.030±0.000	17±1	0.030±0.000	19±1	0.018±0.000	31±1	0.024±0.000	20±1	0.014±0.000	23±1	0.008±0.000	22±0	0.007±0.000	23±0
tgrO1	0.033±0.000	19±1	0.030±0.000	24±1	0.020±0.000	30±1	0.020±0.000	22±1	0.015±0.000	25±0	0.005±0.000	26±0	0.006±0.000	26±0
tgrC1	0.032±0.000	22±0	0.033±0.001	22±1	0.020±0.000	32±1	0.026±0.000	21±1	0.010±0.000	26±0	0.003±0.000	25±0	0.006±0.000	30±0
comC	0.030±0.000	24±1	0.035±0.001	23±1	0.019±0.000	31±0	0.021±0.000	24±1	0.021±0.000	25±1	0.010±0.000	26±0	0.008±0.000	26±0
tspE	0.025±0.000	21±1	0.021±0.001	25±1	0.015±0.000	30±0	0.015±0.000	30±1	0.018±0.001	24±1	0.009±0.000	28±1	0.010±0.000	31±1
car2	0.021±0.000	25±1	0.024±0.000	33±0	0.015±0.000	36±1	0.013±0.000	25±0	0.012±0.000	28±0	0.003±0.000	33±0	0.006±0.000	33±0
car1	0.026±0.000	18±1	0.023±0.000	35±1	0.012±0.000	35±1	0.012±0.000	25±1	0.016±0.000	32±0	0.010±0.000	31±1	0.010±0.000	36±1
fscD	0.024±0.000	28±1	0.027±0.000	28±0	0.014±0.000	34±1	0.012±0.000	28±0	0.014±0.000	26±0	0.004±0.000	31±1	0.010±0.000	28±1
car4	0.021±0.000	23±1	0.021±0.000	26±1	0.016±0.000	29±1	0.013±0.000	27±1	0.013±0.000	29±1	0.004±0.000	29±0	0.006±0.000	29±0
fscE	0.019±0.000	22±1	0.024±0.000	27±0	0.018±0.000	26±1	0.012±0.000	23±0	0.013±0.000	28±0	0.006±0.000	31±1	0.010±0.000	31±1
fscF	0.021±0.001	24±1	0.019±0.000	32±1	0.017±0.000	28±1	0.012±0.000	27±1	0.015±0.000	26±1	0.005±0.000	32±1	0.010±0.000	34±0
car3	0.025±0.000	26±0	0.023±0.000	27±0	0.020±0.000	30±0	0.014±0.000	30±0	0.014±0.000	31±0	0.003±0.000	33±1	0.006±0.000	31±0
fscA	0.021±0.001	23±1	0.013±0.000	30±1	0.015±0.000	29±1	0.013±0.000	24±0	0.011±0.000	31±1	0.004±0.000	32±1	0.006±0.000	41±1
fslG	0.023±0.000	19±1	0.026±0.000	29±1	0.022±0.000	27±1	0.008±0.000	30±0	0.015±0.000	27±0	0.005±0.000	28±1	0.008±0.000	31±0
fslQ	0.023±0.000	21±1	0.017±0.001	27±1	0.012±0.000	33±1	0.013±0.000	26±0	0.011±0.000	33±0	0.007±0.000	27±0	0.005±0.000	42±0
fslF	0.023±0.000	26±0	0.021±0.000	28±1	0.015±0.001	37±1	0.009±0.000	33±0	0.003±0.000	44±1	0.004±0.000	36±1	0.008±0.000	35±0
fslJ	0.022±0.000	23±1	0.021±0.000	25±1	0.018±0.000	25±0	0.014±0.000	25±0	0.011±0.000	32±0	0.009±0.000	24±1	0.005±0.000	41±0
fslE	0.026±0.000	22±1	0.024±0.001	25±1	0.020±0.000	22±0	0.011±0.000	31±1	0.008±0.000	31±1	0.003±0.000	30±1	0.003±0.000	34±0
fslN	0.022±0.000	24±1	0.019±0.000	29±1	0.017±0.000	29±1	0.011±0.000	26±0	0.013±0.000	30±1	0.007±0.000	26±0	0.008±0.000	35±0
fslL	0.025±0.000	24±1	0.024±0.000	24±1	0.008±0.000	30±0	0.009±0.000	31±0	0.009±0.000	29±0	0.003±0.000	33±0	0.003±0.000	36±0
fslH	0.028±0.000	21±1	0.019±0.000	25±0	0.016±0.000	28±1	0.014±0.000	26±1	0.011±0.000	26±0	0.004±0.000	29±0	0.006±0.000	28±0
fslM	0.022±0.000	26±1	0.022±0.000	29±1	0.017±0.000	28±1	0.010±0.000	25±0	0.012±0.000	33±0	0.005±0.000	30±0	0.008±0.000	35±0
grlB	0.028±0.001	12±2	0.018±0.001	25±2	0.009±0.000	33±1	0.016±0.001	28±2	0.009±0.000	28±1	0.004±0.000	27±1	0.010±0.001	34±1
grlO	0.028±0.001	13±2	0.017±0.000	25±1	0.012±0.001	32±1	0.019±0.000	23±1	0.011±0.000	25±0	0.008±0.000	24±0	0.009±0.000	30±0
slc44a2	0.021±0.001	25±1	0.023±0.001	23±1	0.011±0.000	34±1	0.012±0.001	27±1	0.010±0.000	27±1	0.005±0.000	29±0	0.007±0.000	31±1
patB	0.021±0.000	21±1	0.027±0.000	23±1	0.018±0.000	23±0	0.012±0.000	28±0	0.013±0.000	29±0	0.002±0.000	31±0	0.004±0.000	29±0

For each experimental condition, 10 cells were observed under TIRFM and recorded for 100 frames at a rate of 30 frames/sec, from which ~3000 single-molecule tracks with ~80000 displacements at 33-msec intervals were obtained for the diffusion analysis. The estimated values are shown as means±95% confidence intervals (CI). To calculate CI, the bootstrap method was used (see Methods). ε_M is the localization error of diffusing fluorescent spots and determined using Eq. (2).

Table S3. Diffusion coefficients D_S estimated from HMM and MSD analysis of the 27 membrane proteins under drug-treated conditions.

		Ctrl			Jasplakinolide			Latrunculin A			Nocodazole			Thiabendazole			Benomyl			Blebbistatin		
Gene	State	D_S ($\mu m^2/s$)	p_S (%)	ε_S (nm)	D_S ($\mu m^2/s$)	p_S (%)	ε_S (nm)	D_S ($\mu m^2/s$)	p_S (%)	ε_S (nm)	D_S ($\mu m^2/s$)	p_S (%)	ε_S (nm)	D_S ($\mu m^2/s$)	p_S (%)	ε_S (nm)	D_S ($\mu m^2/s$)	p_S (%)	ε_S (nm)	D_S ($\mu m^2/s$)	p_S (%)	ε_S (nm)
	fast	0.049±0.012	7.2±0.6	74±4	0.090±0.020	5.7±0.4	83±7	0.046±0.023	6.2±0.4	103±6	0.009±0.007	4.9±0.3	108±3	0.007±0.008	6.3±0.4	99±3	0.027±0.023	6.2±0.3	108±6	0.046±0.022	5.7±0.3	97±7
DD3-3	middle	0.028±0.001	60.6±2.7	12±3	0.031±0.001	57.7±2.3	18±3	0.020±0.001	49.5±3.2	29±3	0.027±0.001	50.6±1.6	11±5	0.017±0.001	48.6±1.9	24±2	0.007±0.001	40.1±1.8	36±1	0.017±0.001	46.1±1.8	26±2
	slow	0.007±0.001	32.2±3.2	18±1	0.012±0.001	36.6±2.7	14±1	0.009±0.001	44.4±3.5	17±2	0.009±0.000	44.6±1.8	14±1	0.006±0.000	45.2±2.0	17±1	0.004±0.000	53.7±1.9	17±1	0.006±0.000	48.2±2.1	16±0
	fast	0.111±0.026	5.9±0.4	64±12	0.104±0.011	6.0±0.4	63±3	0.080±0.025	6.5±0.4	81±9	0.077±0.013	6.5±0.6	70±5	0.072±0.012	5.7±0.2	64±5	0.043±0.008	4.8±0.4	69±2	0.050±0.013	6.1±0.3	62±3
psiM	middle	0.038±0.001	69.4±1.3	8±2	0.034±0.001	71.6±1.0	6±2	0.025±0.001	46.9±2.2	23±3	0.027±0.001	72.3±3.9	3±3	0.019±0.000	48.8±1.5	15±2	0.011±0.000	42.3±1.4	21±1	0.010±0.000	42.0±1.9	17±1
	slow	0.013±0.001	24.6±1.5	13±2	0.010±0.000	22.4±1.4	10±2	0.011±0.001	46.6±2.5	9±1	0.006±0.001	21.2±4.4	13±1	0.007±0.000	45.5±1.7	10±1	0.005±0.000	52.9±1.7	11±0	0.004±0.000	51.9±2.1	12±0
	fast	0.087±0.020	5.9±0.5	80±6	0.087±0.023	5.6±0.2	92±7	0.085±0.012	5.9±0.4	81±4	0.032±0.009	5.0±0.3	90±3	0.054±0.006	6.0±0.2	78±2	0.025±0.003	5.9±0.3	73±1	0.038±0.005	6.8±0.3	68±2
tgrO1	middle	0.038±0.001	65.2±2.1	10±3	0.039±0.001	52.4±1.0	17±3	0.026±0.001	48.3±1.0	27±2	0.027±0.001	51.9±1.3	9±3	0.020±0.001	51.2±1.0	14±2	0.005±0.000	43.9±0.5	23±0	0.007±0.000	41.5±0.9	23±0
	slow	0.012±0.001	28.9±2.6	10±2	0.015±0.000	42.0±1.1	8±1	0.010±0.000	45.8±1.2	14±0	0.009±0.000	43.1±1.6	12±2	0.006±0.000	42.8±1.2	12±0	0.003±0.000	50.2±0.7	12±0	0.003±0.000	51.7±1.1	12±0
	fast	0.100±0.007	6.1±0.3	76±4	0.081±0.007	8.0±0.3	85±4	0.036±0.008	7.3±0.5	92±4	0.059±0.007	6.2±0.5	84±4	0.017±0.007	5.9±0.1	87±6	0.004±0.006	4.9±0.2	84±3	0.032±0.007	6.7±0.2	83±4
tgrC1	middle	0.038±0.001	65.7±1.2	9±2	0.037±0.001	71.1±0.7	11±2	0.024±0.000	55.7±1.2	26±1	0.033±0.001	60.7±1.6	13±3	0.013±0.001	44.3±1.1	20±2	0.003±0.000	38.3±1.1	26±1	0.007±0.001	36.4±0.8	28±1
	slow	0.012±0.001	28.2±1.3	11±3	0.007±0.001	20.9±0.9	15±1	0.010±0.001	37.0±1.6	12±1	0.011±0.001	33.1±2.0	11±2	0.005±0.000	49.9±1.2	12±1	0.002±0.000	56.8±1.2	12±0	0.003±0.000	56.9±0.9	13±0
	fast	0.095±0.014	6.8±0.3	81±6	0.110±0.024	8.3±0.4	76±11	0.076±0.010	7.1±0.5	82±2	0.037±0.006	5.8±0.5	88±3	0.058±0.014	5.5±0.2	86±5	0.031±0.005	5.7±0.4	80±3	0.042±0.006	6.3±0.3	73±2
comC	middle	0.036±0.001	60.7±1.4	12±3	0.038±0.001	67.9±1.2	7±2	0.025±0.001	56.7±1.8	23±3	0.026±0.001	56.8±2.2	15±2	0.029±0.001	51.8±1.6	5±3	0.013±0.000	45.8±1.1	23±1	0.011±0.000	42.9±1.0	23±1
	slow	0.011±0.001	32.5±1.5	13±1	0.010±0.001	23.8±1.4	14±1	0.007±0.001	36.2±2.3	17±0	0.010±0.001	37.4±2.6	10±2	0.010±0.000	42.7±1.8	11±1	0.006±0.000	48.5±1.3	12±1	0.005±0.000	50.8±1.2	12±0
	fast	0.037±0.014	3.8±0.3	97±4	0.050±0.011	4.8±0.5	87±6	0.044±0.015	5.2±0.5	92±6	0.038±0.003	7.0±0.3	86±3	0.057±0.024	3.8±0.4	93±7	0.009±0.011	4.2±0.7	88±7	0.102±0.019	7.4±0.6	62±7
tspE	middle	0.034±0.002	48.8±2.3	15±4	0.028±0.001	64.5±2.9	12±3	0.021±0.001	41.3±1.9	28±2	0.023±0.001	50.4±1.2	15±2	0.026±0.002	61.5±2.7	2±3	0.014±0.001	58.9±1.5	22±1	0.015±0.001	41.6±1.7	23±2
	slow	0.017±0.001	47.4±2.5	4±2	0.007±0.001	30.7±3.2	15±1	0.011±0.000	53.6±2.3	15±1	0.007±0.000	42.6±1.5	16±0	0.007±0.001	34.8±3.0	14±1	0.003±0.000	36.9±2.0	18±1	0.006±0.000	51.0±2.3	14±1
	fast	0.063±0.016	4.4±0.4	87±6	0.081±0.008	7.7±0.3	91±3	0.056±0.020	6.1±0.1	98±6	0.019±0.007	4.3±0.2	96±3	0.041±0.008	7.1±0.3	80±3	0.008±0.005	7.0±0.4	89±2	0.035±0.010	7.0±0.3	86±2
car2	middle	0.028±0.001	54.6±3.1	17±4	0.031±0.001	52.7±0.6	20±1	0.021±0.001	50.3±1.0	33±2	0.018±0.000	42.9±1.4	24±1	0.016±0.001	52.4±1.0	19±1	0.003±0.000	44.7±1.2	31±1	0.007±0.000	43.4±1.3	29±1
	slow	0.011±0.001	40.9±3.5	13±1	0.010±0.000	39.6±0.8	16±1	0.009±0.000	43.6±1.0	14±1	0.008±0.000	52.8±1.6	11±1	0.005±0.000	40.5±1.2	14±1	0.002±0.000	48.3±1.5	16±0	0.003±0.000	49.5±1.5	16±0
	fast	0.071±0.013	4.0±0.3	88±5	0.082±0.044	7.1±0.3	103±13	0.034±0.017	5.2±0.3	98±6	0.025±0.003	5.4±0.3	82±2	0.045±0.008	6.4±0.2	97±3	0.029±0.012	5.9±0.4	94±5	0.046±0.011	8.1±0.2	93±4
car1	middle	0.034±0.001	52.8±2.5	11±5	0.030±0.001	49.1±1.1	30±4	0.016±0.002	49.1±1.3	33±3	0.016±0.000	49.6±1.0	17±1	0.020±0.001	46.2±1.3	27±2	0.012±0.001	45.3±1.5	26±1	0.013±0.001	45.5±1.1	30±2
	slow	0.013±0.001	43.3±2.7	10±2	0.012±0.001	43.8±1.3	14±1	0.008±0.000	45.7±1.4	16±1	0.007±0.000	45.0±1.1	11±1	0.007±0.000	47.4±1.4	16±1	0.005±0.000	48.7±1.8	14±1	0.006±0.000	46.4±1.2	13±1
	fast	0.052±0.016	5.7±0.2	96±5	0.041±0.010	7.1±0.3	96±4	0.022±0.013	5.3±0.3	107±3	0.021±0.007	5.7±0.3	90±6	0.052±0.014	5.3±0.3	81±5	0.018±0.010	6.1±0.2	88±3	0.035±0.009	5.1±0.2	89±3
fscD	middle	0.030±0.001	59.7±1.8	20±1	0.033±0.001	64.0±1.1	6±3	0.020±0.001	47.9±1.6	30±1	0.017±0.000	46.0±2.0	22±1	0.017±0.001	48.6±1.6	23±2	0.004±0.000	40.1±1.1	29±1	0.014±0.001	42.1±1.3	24±2
	slow	0.010±0.001	34.6±2.0	15±1	0.011±0.001	29.0±1.2	12±3	0.009±0.000	46.8±1.8	14±1	0.007±0.000	48.3±2.3	14±1	0.008±0.000	46.2±1.9	10±1	0.002±0.000	53.8±1.1	15±0	0.007±0.000	52.8±1.4	12±1
	fast	0.043±0.012	4.2±0.2	90±3	0.052±0.009	6.3±0.3	86±3	0.088±0.025	5.7±0.3	72±10	0.031±0.013	4.4±0.3	91±4	0.031±0.007	5.8±0.2	93±7	0.008±0.005	6.2±0.6	84±4	0.029±0.006	5.8±0.3	87±3
car4	middle	0.028±0.001	54.6±2.9	15±2	0.027±0.001	63.3±1.7	13±2	0.022±0.001	52.7±1.9	17±2	0.019±0.001	41.2±2.0	24±4	0.017±0.001	45.3±1.1	25±2	0.006±0.000	51.0±3.4	25±2	0.008±0.000	41.9±1.3	25±1
	slow	0.011±0.001	41.2±3.1	12±1	0.006±0.001	30.4±2.0	15±1	0.010±0.000	41.6±2.2	13±1	0.008±0.000	54.4±2.2	14±1	0.007±0.000	48.9±1.2	13±1	0.002±0.000	42.8±3.9	16±0	0.004±0.000	52.3±1.5	14±0
	fast	0.034±0.010	3.9±0.2	92±4	0.024±0.008	3.9±0.2	111±2	0.055±0.016	4.3±0.2	89±4	0.024±0.008	3.9±0.2	91±3	0.058±0.008	4.8±0.3	87±3	0.012±0.006	5.3±0.4	92±4	0.033±0.010	5.7±0.2	91±3
fscE	middle	0.027±0.001	51.6±2.2	14±1	0.035±0.001	48.0±1.3	16±3	0.023±0.001	44.9±1.6	26±2	0.016±0.000	40.7±1.2	24±1	0.016±0.001	42.7±1.6	30±2	0.006±0.000	42.9±1.6	31±1	0.013±0.001	45.4±0.8	26±2
	slow	0.010±0.000	44.5±2.4	12±1	0.015±0.001	48.1±1.4	12±2	0.011±0.000	50.8±1.8	11±1	0.009±0.000	55.4±1.2	10±1	0.009±0.000	52.5±1.8	13±1	0.004±0.000	51.8±2.0	16±0	0.006±0.000	48.8±0.9	15±0
	fast	0.038±0.015	3.6±0.3	92±7	0.053±0.007	6.3±0.4	93±3	0.041±0.012	5.7±0.2	86±9	0.063±0.023	7.0±0.7	64±20	0.026±0.006	4.8±0.5	91±4	0.010±0.006	5.9±0.6	91±4	0.034±0.012	7.7±0.4	90±6
fscF	middle	0.027±0.001	56.3±2.5	20±2	0.024±0.001	52.5±1.8	29±2	0.022±0.001	49.1±1.7	24±1	0.015±0.000	52.6±4.3	23±2	0.021±0.001	50.4±2.5	19±3	0.008±0.000	39.9±1.2	27±1	0.013±0.000	53.9±1.1	27±1
	slow	0.011±0.001	40.0±2.6	13±2	0.011±0.001	41.2±2.1	16±1	0.011±0.001	45.2±1.9	12±1	0.007±0.001	40.4±4.9	12±1	0.009±0.001	44.8±2.9	11±2	0.004±0.000	54.1±1.4	16±0	0.005±0.000	38.4±1.4	17±0
	fast	0.059±0.013	5.4±0.5	92±5	0.059±0.008	5.8±0.3	92±4	0.055±0.013	5.9±0.2	88±5	0.058±0.008	6.0±0.3	84±2	0.043±0.004	7.9±0.4	87±2	0.031±0.022	7.2±0.4	84±7	0.037±0.011	6.5±0.1	83±4
car3	middle	0.034±0.002	55.7±3.5	13±3	0.031±0.001	56.3±1.2	13±4	0.029±0.001	51.5±1.1	17±1	0.019±0.000	47.3±1.3	25±1	0.019±0.001	50.3±1.9	22±3	0.003±0.000	43.6±1.7	29±1	0.009±0.000	39.2±0.8	28±1
	slow	0.011±0.001	38.9±4.0	14±2	0.010±0.001	37.9±1.3	15±1	0.010±0.000	42.6±1.3	13±1	0.008±0.000	46.7±1.6	14±1	0.006±0.001	41.9±2.2	15±1	0.002±0.000	49.1±2.0	15±0	0.004±0.000	54.3±0.9	15±0
	fast	0.044±0.009	5.9±1.0	72±7	0.043±0.063	5.4±0.5	84±26	0.021±0.023	4.3±0.2	103±8	0.035±0.010	4.5±0.5	83±7	0.009±0.006	7.0±0.5	93±4	0.036±0.007	6.1±0.8	81±6	0.021±0.007	8.7±0.3	104±2
fscA	middle	0.025±0.002	67.9±5.9	15±2	0.016±0.001	49.3±4.1	28±3	0.022±0.001	41.1±2.6	27±3	0.019±0.001	52.4±1.8	17±2	0.017±0.001	48.7±1.9	25±2	0.006±0.001	41.6±1.9	30±1	0.009±0.001	46.0±1.3	32±2
	slow	0.008±0.002	26.2±6.6	15±2	0.007±0.001	45.3±4.5	15±2	0.010±0.001	54.6±2.8	15±1	0.007±0.001	43.1±2.1	11±1	0.006±0.000	44.2±2.3	14±2	0.003±0.000	52.3±2.5	16±1	0.004±0.000	45.3±1.6	17±1

Journal of Cell Science • Supplementary information

		Ctrl			Jasplakinolide			Latrunculin A			Nocodazole			Thiabendazole			Benomyl			Blebbistatin		
Gene	State	D_S (μm^2/s)	p_S (%)	ε_S (nm)	D_S (μm^2/s)	p_S (%)	ε_S (nm)	D_S (μm^2/s)	p_S (%)	ε_S (nm)	D_S (μm^2/s)	p_S (%)	ε_S (nm)	D_S (μm^2/s)	p_S (%)	ε_S (nm)	D_S (μm^2/s)	p_S (%)	ε_S (nm)	D_S (μm^2/s)	p_S (%)	ε_S (nm)
	fast	0.055±0.028	2.8±0.2	95±9	0.067±0.014	5.1±0.2	98±5	0.035±0.009	5.2±0.2	99±3	0.012±0.016	5.1±0.3	96±6	0.049±0.021	5.0±0.3	86±7	0.022±0.007	5.6±0.4	83±5	0.032±0.006	6.3±0.3	88±3
fslG	middle	0.031±0.001	49.0±2.8	19±3	0.033±0.001	53.1±1.6	25±3	0.029±0.001	45.3±2.4	25±3	0.009±0.000	42.3±1.3	31±1	0.018±0.001	45.9±1.2	28±2	0.006±0.000	43.2±1.6	25±1	0.010±0.001	43.9±1.0	28±1
	slow	0.014±0.001	48.2±2.9	5±2	0.014±0.001	41.8±1.7	12±2	0.016±0.000	49.5±2.6	6±2	0.006±0.000	52.7±1.5	14±1	0.010±0.000	49.1±1.2	9±2	0.003±0.000	51.2±1.9	13±0	0.005±0.000	49.8±1.2	15±1
	fast	0.031±0.011	4.1±0.2	96±5	0.029±0.018	6.6±0.7	88±6	0.013±0.015	4.6±0.2	104±4	0.037±0.007	5.4±0.4	81±3	0.015±0.004	6.9±0.3	98±3	0.021±0.005	4.7±0.2	86±2	0.032±0.008	11.3±0.4	95±6
fslQ	middle	0.031±0.001	52.7±1.5	11±3	0.023±0.001	56.4±3.3	20±3	0.016±0.001	45.0±1.1	32±1	0.017±0.001	43.6±1.4	23±2	0.015±0.001	43.8±1.4	25±2	0.009±0.000	40.1±0.7	27±1	0.007±0.001	45.7±1.2	35±1
	slow	0.013±0.000	43.2±1.6	8±2	0.009±0.001	37.0±3.9	12±1	0.009±0.000	50.4±1.2	16±1	0.009±0.000	51.0±1.7	11±1	0.007±0.000	49.3±1.4	13±1	0.005±0.000	55.2±0.8	13±0	0.004±0.000	43.0±1.3	17±1
	fast	0.026±0.014	4.9±0.2	103±5	0.043±0.009	7.6±0.5	90±5	0.056±0.007	8.8±0.3	89±2	0.033±0.009	6.6±0.4	93±2	0.089±0.029	8.4±0.2	86±11	0.017±0.009	7.0±0.3	98±3	0.025±0.006	6.9±0.4	95±2
fslF	middle	0.031±0.001	50.6±1.9	21±3	0.025±0.001	60.2±2.7	20±2	0.020±0.001	55.9±1.1	27±1	0.011±0.001	43.9±1.6	30±1	0.005±0.000	41.5±1.3	36±1	0.005±0.000	41.0±1.2	33±1	0.009±0.001	45.9±1.7	34±2
	slow	0.012±0.000	44.6±2.0	12±1	0.008±0.001	32.2±3.2	15±1	0.007±0.000	35.3±1.3	18±1	0.006±0.000	49.5±1.8	16±0	0.002±0.000	50.0±1.4	18±0	0.003±0.000	52.0±1.4	17±0	0.004±0.000	47.2±2.0	18±0
	fast	0.031±0.023	3.3±0.2	105±6	0.047±0.015	4.4±0.3	94±5	0.045±0.008	5.2±0.3	88±3	0.040±0.008	4.9±0.5	85±4	0.027±0.006	6.5±0.2	93±3	0.020±0.006	4.6±0.4	84±2	0.043±0.006	8.6±0.3	92±7
fslJ	middle	0.030±0.001	42.8±2.1	26±3	0.029±0.001	51.5±1.5	15±2	0.023±0.001	44.4±1.9	25±2	0.019±0.001	47.5±2.5	17±2	0.014±0.000	51.1±0.9	26±1	0.012±0.001	46.4±2.1	22±1	0.006±0.001	50.1±1.4	35±1
	slow	0.015±0.000	53.9±2.2	9±3	0.011±0.001	44.2±1.6	13±2	0.012±0.000	50.4±2.2	6±2	0.009±0.001	47.6±2.9	10±1	0.007±0.000	42.5±1.0	15±1	0.006±0.000	49.0±2.4	12±1	0.003±0.000	41.3±1.6	20±0
	fast	0.068±0.024	2.9±0.3	92±7	0.078±0.039	4.1±0.3	87±16	0.039±0.010	3.7±0.3	90±4	-0.004±0.012	4.7±0.3	113±6	0.015±0.007	5.6±0.4	93±6	0.001±0.011	4.3±0.2	97±3	0.034±0.015	6.3±0.2	89±7
fslE	middle	0.033±0.001	58.4±2.9	16±4	0.031±0.001	56.6±1.7	15±4	0.027±0.001	47.0±2.9	16±3	0.014±0.001	46.0±4.2	31±4	0.010±0.001	43.4±1.2	29±1	0.004±0.000	43.9±2.1	30±1	0.005±0.001	39.8±1.8	32±1
	slow	0.017±0.002	38.7±3.1	4±3	0.014±0.000	39.3±1.7	8±2	0.014±0.001	49.3±3.2	7±2	0.008±0.001	49.3±4.3	15±1	0.005±0.000	51.0±1.4	16±0	0.003±0.000	51.7±2.1	16±1	0.003±0.000	53.9±1.9	18±0
	fast	0.046±0.006	5.1±0.3	87±3	0.028±0.015	6.0±0.3	98±5	0.039±0.007	6.4±0.6	89±3	0.045±0.014	5.0±0.3	85±3	0.014±0.011	5.1±0.3	100±5	0.008±0.006	4.8±0.2	88±2	0.015±0.010	6.3±0.4	100±4
fslN	middle	0.029±0.001	57.2±3.4	13±3	0.024±0.001	52.2±1.3	23±2	0.022±0.001	56.6±3.1	20±2	0.017±0.001	47.3±1.1	19±2	0.018±0.001	45.3±2.2	26±3	0.009±0.000	44.2±1.6	23±1	0.009±0.000	46.9±1.0	32±1
	slow	0.012±0.001	37.7±3.6	7±2	0.012±0.000	41.7±1.4	7±2	0.008±0.001	36.9±3.6	15±1	0.007±0.000	47.7±1.4	12±1	0.009±0.000	49.5±2.2	13±1	0.005±0.000	51.0±1.8	12±0	0.005±0.000	46.8±1.3	18±0
	fast	0.039±0.009	2.8±0.2	103±3	0.044±0.011	3.8±0.3	100±4	0.015±0.004	6.5±0.3	86±2	0.019±0.008	5.8±0.4	95±4	0.004±0.008	6.7±0.6	89±5	0.015±0.015	6.1±0.5	87±4	0.023±0.005	8.6±0.5	87±3
fslL	middle	0.033±0.002	53.0±2.4	21±3	0.031±0.002	47.5±2.1	26±4	0.010±0.000	44.7±0.7	28±1	0.013±0.001	46.4±1.4	27±1	0.012±0.000	50.9±3.0	24±2	0.003±0.000	45.6±1.3	32±1	0.004±0.000	43.7±1.5	32±1
	slow	0.017±0.000	44.1±2.6	5±3	0.015±0.000	48.7±2.4	11±2	0.006±0.000	48.9±0.9	15±0	0.006±0.000	47.8±1.7	15±1	0.005±0.001	42.4±3.5	14±1	0.003±0.000	48.3±1.7	17±0	0.002±0.000	47.7±2.0	18±1
	fast	0.024±0.017	3.5±0.5	109±6	0.053±0.008	4.9±0.3	89±4	0.030±0.017	4.0±0.3	99±4	0.060±0.012	5.2±0.3	77±7	0.026±0.006	5.3±0.3	86±3	0.004±0.004	6.0±0.2	94±2	0.024±0.007	6.3±0.2	81±4
fslH	middle	0.040±0.001	46.3±3.0	20±6	0.024±0.001	49.0±2.0	21±2	0.021±0.001	41.9±1.6	33±1	0.019±0.001	47.8±1.9	22±2	0.014±0.000	47.4±1.6	24±1	0.004±0.000	45.1±0.8	25±0	0.008±0.000	41.6±0.7	26±1
	slow	0.018±0.001	50.3±3.4	4±2	0.012±0.000	46.2±2.3	8±1	0.012±0.000	54.1±1.8	13±1	0.009±0.000	47.0±2.1	12±1	0.006±0.000	47.4±1.9	13±1	0.003±0.000	48.8±0.9	13±0	0.004±0.000	52.1±0.8	13±0
	fast	0.045±0.014	5.0±0.3	94±5	0.050±0.011	5.6±0.4	96±6	0.030±0.008	5.8±0.2	93±3	0.030±0.006	4.4±0.1	85±3	0.034±0.010	7.0±0.3	93±3	0.028±0.026	6.0±0.4	84±6	0.019±0.004	6.7±0.3	94±2
fslM	middle	0.028±0.001	53.9±2.5	21±2	0.032±0.001	48.9±2.1	15±3	0.023±0.000	53.3±1.6	22±2	0.013±0.000	41.8±0.8	24±1	0.018±0.001	52.0±1.4	23±2	0.006±0.000	39.1±1.3	29±1	0.010±0.001	47.5±1.2	31±1
	slow	0.012±0.001	41.1±2.8	11±2	0.013±0.001	45.5±2.4	14±1	0.008±0.000	40.9±1.6	15±1	0.007±0.000	53.8±0.8	11±1	0.006±0.000	41.0±1.6	17±1	0.004±0.000	54.9±1.6	15±0	0.005±0.000	45.8±1.5	17±1
	fast	0.009±0.017	2.1±0.2	104±5	-0.012±0.012	1.8±0.2	124±5	-0.008±0.015	4.3±0.3	110±3	0.016±0.011	2.5±0.2	123±5	-0.019±0.009	3.0±0.5	109±6	0.027±0.009	3.9±0.4	82±5	-0.031±0.016	4.1±0.3	120±5
grlB	middle	0.038±0.003	43.2±4.1	17±4	0.026±0.003	46.2±4.2	23±5	0.013±0.001	45.4±1.4	27±3	0.027±0.002	48.0±5.0	12±6	0.012±0.001	38.1±8.0	30±4	0.005±0.001	38.8±3.8	30±2	0.015±0.001	41.8±1.9	31±3
	slow	0.020±0.001	54.7±4.2	10±3	0.015±0.001	52.0±4.3	8±3	0.006±0.000	50.3±1.5	17±1	0.013±0.001	49.5±5.1	11±2	0.007±0.001	58.9±8.4	17±1	0.004±0.000	57.2±3.9	15±1	0.007±0.000	54.0±1.8	18±0
	fast	0.101±0.037	2.9±0.4	70±16	0.001±0.015	3.1±0.3	105±6	0.058±0.014	3.5±0.1	96±7	0.029±0.040	2.7±0.4	102±11	0.048±0.018	3.1±0.3	80±8	0.013±0.005	4.5±0.3	80±3	0.054±0.007	4.4±0.3	88±3
grlO	middle	0.039±0.003	53.2±3.9	15±5	0.024±0.001	56.5±3.2	18±3	0.020±0.001	49.6±2.3	25±2	0.028±0.002	58.2±5.2	7±3	0.015±0.001	49.2±2.2	23±1	0.011±0.001	41.9±3.0	21±2	0.016±0.001	37.0±0.7	26±2
	slow	0.016±0.001	43.9±4.2	8±2	0.009±0.001	40.4±3.5	15±1	0.008±0.000	46.8±2.4	16±1	0.009±0.001	39.1±5.5	18±1	0.007±0.000	47.7±2.3	12±1	0.006±0.000	53.5±3.2	13±1	0.006±0.000	58.5±0.9	16±0
	fast	0.018±0.016	3.1±0.4	98±8	0.030±0.011	2.8±0.6	98±7	0.012±0.008	6.1±0.5	100±3	0.040±0.011	4.5±0.3	82±5	-0.012±0.016	3.0±0.3	103±8	0.031±0.023	4.6±0.6	89±14	0.064±0.015	5.6±0.5	76±5
slc44a2	middle	0.026±0.002	57.0±5.7	23±4	0.030±0.001	56.1±3.7	23±2	0.016±0.001	47.8±2.7	30±2	0.019±0.001	49.1±1.8	23±2	0.015±0.001	46.8±2.3	25±2	0.007±0.000	38.0±1.8	29±2	0.009±0.000	40.5±2.8	31±2
	slow	0.012±0.001	39.9±6.1	14±1	0.012±0.002	41.1±4.2	17±1	0.007±0.001	46.0±2.9	19±1	0.007±0.001	46.4±1.8	17±1	0.007±0.000	50.1±2.5	15±1	0.004±0.000	57.4±2.2	16±1	0.006±0.000	53.9±3.3	16±1
	fast	0.043±0.011	5.0±0.4	81±5	0.046±0.011	5.0±0.4	101±4	0.054±0.013	3.4±0.2	87±4	0.040±0.007	6.8±0.3	78±4	0.031±0.004	6.8±0.3	85±2	0.007±0.003	7.1±0.2	83±2	0.025±0.006	6.2±0.3	80±2
patB	middle	0.026±0.001	52.9±1.7	16±2	0.035±0.001	49.4±2.2	19±3	0.024±0.001	35.1±1.5	32±4	0.016±0.001	47.7±0.8	20±2	0.017±0.000	48.4±1.2	22±2	0.003±0.000	36.8±1.4	30±1	0.006±0.000	41.2±1.6	27±1
	slow	0.013±0.000	42.1±1.9	9±1	0.016±0.001	45.6±2.5	4±2	0.013±0.000	61.4±1.6	11±1	0.007±0.000	45.5±1.1	12±1	0.006±0.000	44.8±1.4	15±1	0.002±0.000	56.1±1.5	17±0	0.003±0.000	52.6±1.9	15±0

Using the HMM, single-molecule trajectories were separated into three states, from which the MSD was calculated, and D_S was obtained for each state. The estimated values are shown as means±95% confidence intervals (CI). To calculate CI, the bootstrap method was used (see Methods). ε_S is the localization error and determined using Eq. (7).

Journal of Cell Science • Supplementary information

Table S4. Lifetimes of three diffusion states of the 27 membrane proteins under drug-treated conditions.

Gene	State	Ctrl (s)	Jasplakinolide (s)	Latrunculin A (s)	Nocodazole (s)	Thiabendazole (s)	Benomyl (s)	Blebbistatin (s)
	fast	0.066±0.003	0.046±0.003	0.043±0.002	0.052±0.002	0.058±0.004	0.039±0.003	0.049±0.002
DD3-3	middle	0.345±0.025	0.274±0.019	0.155±0.009	0.206±0.008	0.239±0.013	0.156±0.007	0.174±0.009
	slow	0.710±0.071	0.575±0.055	0.380±0.020	0.447±0.024	0.606±0.031	0.531±0.044	0.435±0.028
	fast	0.061±0.003	0.063±0.003	0.066±0.004	0.064±0.003	0.058±0.003	0.069±0.004	0.078±0.002
psiM	middle	0.466±0.024	0.539±0.015	0.257±0.020	0.484±0.034	0.312±0.012	0.326±0.014	0.365±0.013
	slow	0.560±0.039	0.594±0.034	0.692±0.023	0.508±0.013	0.710±0.029	0.844±0.034	0.938±0.039
	fast	0.043±0.002	0.037±0.002	0.043±0.002	0.044±0.003	0.050±0.002	0.069±0.002	0.071±0.003
tgrO1	middle	0.375±0.022	0.221±0.008	0.201±0.007	0.256±0.006	0.299±0.010	0.336±0.008	0.281±0.007
	slow	0.441±0.022	0.373±0.018	0.427±0.009	0.461±0.022	0.618±0.019	0.770±0.017	0.747±0.016
	fast	0.052±0.002	0.044±0.002	0.049±0.003	0.050±0.003	0.053±0.002	0.060±0.003	0.062±0.003
tgrC1	middle	0.355±0.014	0.386±0.006	0.255±0.012	0.313±0.016	0.248±0.008	0.272±0.012	0.226±0.009
	slow	0.376±0.025	0.546±0.025	0.491±0.021	0.431±0.015	0.610±0.021	0.741±0.023	0.779±0.028
	fast	0.040±0.002	0.042±0.002	0.045±0.003	0.049±0.003	0.045±0.003	0.056±0.001	0.070±0.002
comC	middle	0.264±0.014	0.350±0.010	0.244±0.014	0.303±0.018	0.251±0.015	0.262±0.009	0.297±0.013
	slow	0.320±0.011	0.495±0.022	0.389±0.015	0.510±0.016	0.464±0.024	0.591±0.020	0.754±0.027
	fast	0.039±0.003	0.056±0.003	0.063±0.005	0.062±0.003	0.048±0.004	0.063±0.004	0.064±0.004
tspE	middle	0.240±0.016	0.357±0.030	0.193±0.014	0.266±0.014	0.338±0.025	0.348±0.020	0.218±0.014
	slow	0.587±0.021	0.550±0.035	0.638±0.026	0.636±0.047	0.536±0.028	0.722±0.061	0.716±0.027
	fast	0.049±0.003	0.044±0.001	0.039±0.002	0.048±0.002	0.056±0.002	0.069±0.003	0.061±0.003
car2	middle	0.273±0.025	0.205±0.005	0.177±0.008	0.220±0.010	0.301±0.011	0.245±0.011	0.215±0.009
	slow	0.482±0.019	0.373±0.009	0.436±0.017	0.615±0.011	0.683±0.025	0.611±0.035	0.587±0.022
	fast	0.041±0.002	0.038±0.001	0.044±0.002	0.055±0.002	0.039±0.001	0.046±0.003	0.056±0.002
car1	middle	0.261±0.022	0.151±0.008	0.171±0.006	0.301±0.008	0.154±0.007	0.211±0.009	0.191±0.006
	slow	0.442±0.023	0.343±0.011	0.439±0.024	0.735±0.025	0.359±0.012	0.538±0.018	0.531±0.028
	fast	0.040±0.002	0.049±0.002	0.038±0.002	0.057±0.004	0.049±0.003	0.057±0.002	0.057±0.003
fscD	middle	0.250±0.016	0.302±0.010	0.177±0.010	0.214±0.014	0.255±0.009	0.220±0.011	0.236±0.013
	slow	0.334±0.011	0.468±0.018	0.457±0.022	0.547±0.014	0.576±0.026	0.687±0.021	0.699±0.022
	fast	0.049±0.003	0.051±0.002	0.051±0.003	0.056±0.002	0.050±0.002	0.071±0.004	0.058±0.002
car4	middle	0.294±0.025	0.307±0.017	0.248±0.013	0.196±0.014	0.210±0.007	0.334±0.039	0.256±0.010
	slow	0.599±0.031	0.376±0.017	0.541±0.017	0.554±0.023	0.519±0.012	0.635±0.043	0.777±0.032
	fast	0.044±0.002	0.045±0.002	0.049±0.002	0.049±0.003	0.053±0.003	0.059±0.005	0.049±0.001
fscE	middle	0.248±0.018	0.203±0.008	0.196±0.010	0.207±0.008	0.204±0.012	0.224±0.012	0.225±0.006
	slow	0.472±0.018	0.475±0.018	0.511±0.017	0.602±0.024	0.556±0.027	0.627±0.018	0.615±0.017
	fast	0.048±0.003	0.052±0.002	0.068±0.002	0.078±0.005	0.056±0.004	0.058±0.006	0.057±0.003
fscF	middle	0.276±0.029	0.225±0.011	0.261±0.014	0.327±0.051	0.270±0.018	0.197±0.009	0.265±0.011
	slow	0.450±0.037	0.459±0.020	0.617±0.036	0.605±0.037	0.636±0.035	0.696±0.041	0.562±0.024
	fast	0.042±0.002	0.044±0.002	0.051±0.001	0.051±0.003	0.059±0.002	0.062±0.004	0.062±0.002
car3	middle	0.220±0.026	0.249±0.009	0.243±0.007	0.209±0.008	0.205±0.017	0.235±0.014	0.202±0.003
	slow	0.328±0.012	0.404±0.015	0.501±0.013	0.488±0.022	0.404±0.009	0.621±0.041	0.601±0.014
	fast	0.071±0.005	0.070±0.004	0.049±0.003	0.065±0.004	0.072±0.006	0.067±0.005	0.048±0.002
fscA	middle	0.445±0.067	0.265±0.051	0.167±0.014	0.343±0.028	0.254±0.014	0.227±0.011	0.170±0.009
	slow	0.532±0.031	0.507±0.059	0.483±0.033	0.672±0.033	0.591±0.032	0.742±0.053	0.629±0.028

Journal of Cell Science • Supplementary information

Gene	State	Ctrl (s)	Jasplakinolide (s)	Latrunculin A (s)	Nocodazole (s)	Thiabendazole (s)	Benomyl (s)	Blebbistatin (s)
	fast	0.040±0.003	0.045±0.002	0.053±0.002	0.056±0.002	0.048±0.004	0.057±0.005	0.062±0.002
fslG	middle	0.239±0.018	0.205±0.012	0.202±0.013	0.231±0.008	0.215±0.007	0.238±0.012	0.234±0.010
	slow	0.544±0.035	0.363±0.019	0.576±0.019	0.655±0.015	0.548±0.028	0.691±0.033	0.611±0.021
	fast	0.052±0.003	0.058±0.004	0.039±0.003	0.059±0.005	0.050±0.002	0.057±0.002	0.074±0.002
fslQ	middle	0.295±0.024	0.301±0.027	0.176±0.009	0.256±0.014	0.182±0.010	0.260±0.006	0.217±0.009
	slow	0.522±0.034	0.507±0.027	0.499±0.017	0.687±0.027	0.517±0.026	0.803±0.026	0.596±0.024
	fast	0.040±0.002	0.054±0.004	0.055±0.003	0.060±0.004	0.040±0.002	0.055±0.002	0.052±0.002
fslF	middle	0.201±0.015	0.269±0.025	0.208±0.008	0.197±0.011	0.124±0.006	0.197±0.008	0.198±0.008
	slow	0.372±0.011	0.407±0.023	0.390±0.023	0.535±0.027	0.414±0.021	0.712±0.032	0.533±0.023
	fast	0.043±0.003	0.051±0.002	0.054±0.003	0.051±0.004	0.053±0.002	0.061±0.002	0.058±0.002
fslJ	middle	0.203±0.016	0.254±0.015	0.226±0.013	0.279±0.017	0.262±0.008	0.326±0.020	0.214±0.009
	slow	0.558±0.027	0.534±0.041	0.577±0.015	0.680±0.018	0.585±0.019	0.799±0.037	0.479±0.020
	fast	0.041±0.002	0.045±0.002	0.060±0.005	0.040±0.004	0.055±0.003	0.067±0.007	0.060±0.003
fslE	middle	0.303±0.025	0.276±0.017	0.271±0.028	0.161±0.009	0.216±0.009	0.233±0.011	0.211±0.013
	slow	0.517±0.047	0.513±0.042	0.703±0.041	0.497±0.026	0.613±0.029	0.576±0.055	0.839±0.042
	fast	0.060±0.003	0.051±0.002	0.055±0.003	0.059±0.004	0.054±0.003	0.059±0.003	0.052±0.003
fslN	middle	0.307±0.029	0.252±0.013	0.263±0.020	0.269±0.012	0.203±0.021	0.292±0.011	0.206±0.012
	slow	0.487±0.041	0.598±0.027	0.537±0.024	0.634±0.025	0.532±0.024	0.762±0.037	0.585±0.023
	fast	0.038±0.003	0.043±0.003	0.070±0.003	0.057±0.002	0.071±0.006	0.060±0.005	0.068±0.005
fslL	middle	0.243±0.018	0.208±0.014	0.263±0.007	0.259±0.011	0.266±0.028	0.261±0.012	0.216±0.016
	slow	0.476±0.034	0.505±0.014	0.683±0.037	0.712±0.025	0.570±0.032	0.698±0.044	0.680±0.027
	fast	0.038±0.004	0.046±0.002	0.042±0.004	0.050±0.002	0.055±0.002	0.068±0.002	0.065±0.003
fslH	middle	0.178±0.016	0.254±0.016	0.179±0.007	0.252±0.014	0.259±0.014	0.306±0.012	0.274±0.007
	slow	0.416±0.024	0.557±0.015	0.603±0.017	0.640±0.018	0.588±0.014	0.734±0.026	0.795±0.020
	fast	0.041±0.003	0.064±0.004	0.048±0.002	0.050±0.001	0.050±0.003	0.062±0.003	0.057±0.002
fslM	middle	0.238±0.018	0.224±0.014	0.241±0.012	0.241±0.008	0.239±0.013	0.237±0.013	0.231±0.010
	slow	0.407±0.015	0.453±0.030	0.469±0.018	0.684±0.020	0.547±0.023	0.753±0.025	0.668±0.038
	fast	0.039±0.002	0.036±0.003	0.046±0.002	0.043±0.004	0.041±0.010	0.078±0.008	0.038±0.003
grlB	middle	0.229±0.039	0.145±0.013	0.140±0.009	0.138±0.017	0.159±0.030	0.203±0.035	0.131±0.009
	slow	0.684±0.073	0.436±0.070	0.467±0.038	0.355±0.019	0.599±0.094	0.700±0.062	0.415±0.027
	fast	0.047±0.004	0.040±0.003	0.044±0.004	0.040±0.006	0.041±0.004	0.073±0.003	0.055±0.003
grlO	middle	0.341±0.032	0.305±0.027	0.200±0.013	0.331±0.039	0.252±0.012	0.285±0.033	0.190±0.007
	slow	0.671±0.046	0.551±0.046	0.562±0.051	0.559±0.045	0.634±0.033	0.938±0.037	0.730±0.034
	fast	0.053±0.006	0.054±0.007	0.056±0.005	0.063±0.005	0.055±0.006	0.051±0.005	0.065±0.004
slc44a2	middle	0.278±0.048	0.275±0.026	0.192±0.013	0.270±0.015	0.266±0.023	0.210±0.016	0.231±0.023
	slow	0.429±0.016	0.436±0.039	0.507±0.025	0.568±0.047	0.719±0.080	0.849±0.051	0.833±0.047
	fast	0.056±0.004	0.039±0.002	0.050±0.003	0.060±0.003	0.062±0.002	0.077±0.004	0.067±0.002
patB	middle	0.348±0.020	0.221±0.021	0.175±0.016	0.286±0.008	0.262±0.012	0.250±0.021	0.264±0.018
	slow	0.751±0.052	0.446±0.025	0.607±0.024	0.721±0.045	0.587±0.023	0.833±0.027	0.730±0.019

Using the HMM, single-molecule trajectories were separated into three states, and the lifetime of each was calculated. The estimated values are shown as means±95% confidence intervals (CI). To calculate CI, the bootstrap method was used (see Methods).

Journal of Cell Science • Supplementary information

Table S5. Characteristic values of multiple diffusion states estimated from experimental data and field model-based simulations.

		Experiment						Simulation					
**Drug**	**State**	**D_S (μm^2/s)**	**p_S (%)**	**ε_S (nm)**	**Lifetime (s)**	**D_M (μm^2/s)**	**ε_M (nm)**	**D_S (μm^2/s)**	**p_S (%)**	**ε_S (nm)**	**Lifetime (s)**	**D_M (μm^2/s)**	**ε_M (nm)**
	fast	0.062±0.004	4.7±0.0	88±1	0.046±0.000			0.043±0.005	5.6±0.1	68±3	0.046±0.001		
ctrl	middle	0.032±0.000	57.0±0.5	12±0	0.289±0.005	0.025±0.000	23±0	0.030±0.000	58.8±0.2	19±0	0.301±0.004	0.023±0.000	26±0
	slow	0.012±0.000	38.3±0.5	10±0	0.468±0.008			0.009±0.000	35.6±0.3	20±0	0.503±0.005		
	fast	0.061±0.002	6.0±0.1	92±1	0.048±0.001			0.035±0.004	5.7±0.1	75±2	0.051±0.001		
jasplakinolide	middle	0.031±0.000	57.4±0.3	15±0	0.271±0.004	0.025±0.000	23±0	0.029±0.000	58.6±0.4	20±1	0.291±0.006	0.022±0.000	28±0
	slow	0.011±0.000	36.6±0.4	13±0	0.450±0.006			0.008±0.000	35.7±0.4	21±0	0.470±0.005		
	fast	0.046±0.002	5.5±0.1	91±0	0.051±0.001			0.033±0.003	5.9±0.1	69±2	0.051±0.000		
latrunculin A	middle	0.023±0.000	49.1±0.5	24±0	0.219±0.005	0.017±0.000	29±0	0.021±0.000	51.4±0.2	28±0	0.226±0.003	0.015±0.000	31±0
	slow	0.010±0.000	45.3±0.5	13±0	0.513±0.006			0.007±0.000	42.7±0.3	20±0	0.502±0.005		
	fast	0.039±0.002	5.4±0.1	86±0	0.055±0.001			0.016±0.003	3.3±0.1	76±2	0.054±0.002		
nocodazole	middle	0.021±0.000	50.5±0.4	16±0	0.277±0.005	0.015±0.000	30±0	0.018±0.000	53.2±0.3	21±1	0.315±0.004	0.013±0.000	24±0
	slow	0.008±0.000	44.1±0.4	12±0	0.607±0.005			0.006±0.000	43.5±0.3	17±0	0.628±0.007		
	fast	0.037±0.003	6.1±0.1	87±1	0.054±0.001			0.025±0.003	7.7±0.1	65±2	0.054±0.000		
thiabendazole	middle	0.018±0.000	50.4±0.3	21±0	0.253±0.004	0.013±0.000	27±0	0.016±0.000	50.2±0.2	24±0	0.233±0.002	0.012±0.000	29±0
	slow	0.007±0.000	43.5±0.4	13±0	0.549±0.005			0.005±0.000	42.1±0.2	18±0	0.521±0.004		
	fast	0.021±0.001	6.0±0.1	83±1	0.064±0.001			0.012±0.000	7.5±0.1	65±0	0.065±0.001		
benomyl	middle	0.007±0.000	45.4±0.3	25±0	0.278±0.005	0.006±0.000	29±0	0.006±0.000	47.1±0.2	27±0	0.285±0.002	0.005±0.000	28±0
	slow	0.003±0.000	48.6±0.4	14±0	0.695±0.009			0.003±0.000	45.4±0.2	16±0	0.700±0.008		
	fast	0.036±0.004	7.4±0.1	83±2	0.065±0.001			0.019±0.001	9.6±0.1	70±1	0.067±0.000		
blebbistatin	middle	0.009±0.000	47.2±0.2	26±0	0.266±0.003	0.007±0.000	32±0	0.008±0.000	44.9±0.3	29±0	0.234±0.002	0.006±0.000	32±0
	slow	0.004±0.000	45.5±0.3	14±0	0.690±0.006			0.004±0.000	45.5±0.3	17±0	0.697±0.006		

To obtain the average diffusion coefficients and lifetimes of the three states independent of the protein species, the single-molecule trajectories of each diffusion state were collected from the 27 membrane proteins in the control and six drug-treated conditions (Fig. 3E-H). The estimated values are shown as means±95% CI. To calculate CI, the bootstrap method was used (see Methods). D_M and D_S are the diffusion coefficients obtained by the MSD and HMM using Eq. (2) and Eq. (6)-(7), respectively. ε_M and ε_S are the localization errors of diffusing fluorescent spots determined using Eq. (2) and Eq. (7), respectively.

Particle trajectory data obtained by the numerical simulation of the field model were analyzed in the same way as the experimental data to obtain the diffusion coefficient $D_{S\text{-}state}$, the lifetimes of each state, and the diffusion coefficient D_M.

Journal of Cell Science • Supplementary information

Table S6. Parameters of the HMMs estimated from experimental data and field model-based simulations.

Drug	State	Experiment					Simulation				
		D_{HMM} (μm^2/s)	$p_{HMM}0$ (%)	fast → *state* (%)	middle → *state* (%)	slow → *state* (%)	D_{HMM} (μm^2/s)	$p_{HMM}0$ (%)	fast → *state* (%)	middle → *state* (%)	slow → *state* (%)
ctrl	fast	0.209±0.001	15.5±0.2	53.4±0.2	3.2±0.0	0.1±0.0	0.189±0.001	17.1±0.3	51.2±0.4	3.8±0.1	0.0±0.0
	middle	0.036±0.000	75.2±0.2	45.2±0.6	91.1±0.1	6.6±0.1	0.036±0.000	73.8±0.4	46.9±0.7	91.4±0.1	5.8±0.1
	slow	0.016±0.000	9.3±0.2	1.4±0.6	5.6±0.1	93.4±0.1	0.016±0.000	9.0±0.3	1.9±0.6	4.8±0.1	94.2±0.1
jasplakinolide	fast	0.219±0.002	18.3±0.3	55.8±0.6	3.8±0.0	0.0±0.0	0.206±0.002	19.7±0.2	54.6±0.3	3.4±0.0	0.0±0.0
	middle	0.038±0.000	72.7±0.2	43.4±0.6	90.8±0.1	6.6±0.1	0.037±0.000	71.4±0.2	45.0±0.5	91.3±0.2	6.3±0.1
	slow	0.015±0.000	8.9±0.2	0.8±0.2	5.4±0.1	93.4±0.1	0.016±0.000	9.0±0.2	0.4±0.3	5.4±0.2	93.7±0.1
latrunculin A	fast	0.225±0.002	17.5±0.2	58.3±0.5	3.9±0.1	0.0±0.0	0.193±0.001	17.1±0.2	55.9±0.3	4.1±0.1	0.0±0.0
	middle	0.035±0.000	72.6±0.3	41.6±0.5	89.1±0.3	5.5±0.1	0.040±0.000	73.0±0.4	43.5±0.5	89.4±0.1	5.1±0.1
	slow	0.014±0.000	9.8±0.3	0.1±0.1	7.0±0.2	94.5±0.1	0.015±0.000	9.9±0.2	0.6±0.2	6.5±0.1	94.9±0.1
nocodazole	fast	0.198±0.001	19.3±0.2	60.6±0.2	3.5±0.0	0.0±0.0	0.185±0.001	19.1±0.1	57.6±1.0	1.5±0.1	0.0±0.0
	middle	0.029±0.000	69.7±0.2	39.1±0.3	91.4±0.1	4.5±0.1	0.028±0.000	70.1±0.3	42.4±1.0	93.3±0.1	4.2±0.1
	slow	0.012±0.000	11.0±0.2	0.3±0.2	5.1±0.1	95.5±0.1	0.012±0.000	10.8±0.3	0.0±0.0	5.2±0.1	95.8±0.1
thiabendazole	fast	0.208±0.002	20.1±0.1	59.8±0.4	4.1±0.0	0.0±0.0	0.173±0.001	21.9±0.3	57.9±0.2	5.4±0.1	0.0±0.0
	middle	0.029±0.000	69.4±0.3	40.1±0.5	90.1±0.1	5.2±0.0	0.030±0.000	66.4±0.4	41.8±0.4	88.4±0.1	5.4±0.1
	slow	0.011±0.000	10.5±0.2	0.1±0.1	5.7±0.1	94.8±0.0	0.012±0.000	11.7±0.3	0.3±0.2	6.3±0.0	94.6±0.1
benomyl	fast	0.190±0.003	22.7±0.2	66.2±0.4	3.8±0.0	0.0±0.0	0.155±0.001	23.7±0.4	63.5±0.2	4.9±0.0	0.0±0.0
	middle	0.024±0.000	63.5±0.2	33.8±0.4	91.3±0.1	3.7±0.1	0.026±0.000	60.3±0.4	35.3±0.3	90.5±0.0	3.8±0.1
	slow	0.008±0.000	13.8±0.2	0.0±0.0	5.0±0.1	96.3±0.1	0.009±0.000	16.0±0.2	1.2±0.2	4.6±0.1	96.2±0.1
blebbistatin	fast	0.200±0.002	27.2±0.3	67.1±0.2	4.1±0.0	0.0±0.0	0.171±0.001	29.4±0.3	64.8±0.2	6.0±0.1	0.0±0.0
	middle	0.027±0.000	61.7±0.3	32.9±0.2	91.3±0.1	3.6±0.0	0.030±0.000	57.5±0.5	33.3±0.2	89.3±0.1	3.5±0.0
	slow	0.009±0.000	11.1±0.1	0.0±0.0	4.6±0.1	96.3±0.0	0.010±0.000	13.1±0.3	1.9±0.2	4.7±0.1	96.5±0.0

The values of “fast → *state*”, “middle → *state*” and “slow → *state*” represent the state transition probabilities of the HMMs. The estimated values are shown as means±95% confidence intervals (CI). To calculate CI, the bootstrap method was used (see Methods). The corresponding figure for this table is shown in Figure S6.

Table S7. Parameters in each drug condition for the field model-based simulation

Drug	Size of a fast state squares (nm)	Size of a slow state squares (nm)	Ratio of the fast state area (%)	Ratio of the slow state area (%)	$D_{S\text{-fast}}$ (μm²/s)	$D_{S\text{-middle}}$ (μm²/s)	$D_{S\text{-slow}}$ (μm²/s)	$\varepsilon_{S\text{-fast}}$ (nm)	$\varepsilon_{S\text{-middle}}$ (nm)	$\varepsilon_{S\text{-slow}}$ (nm)
ctrl	50	250	5	20	0.062	0.032	0.012	88	12	10
jasplakinolide	75	225	6	19	0.061	0.031	0.011	92	15	13
latrunculin A	50	250	6	26	0.046	0.023	0.010	91	24	13
nocodazole	100	250	5	25	0.039	0.021	0.008	86	16	12
thiabendazole	50	200	8	23	0.037	0.018	0.007	87	21	13
benomyl	50	200	10	27	0.021	0.007	0.003	83	25	14
blebbistatin	75	225	16	24	0.036	0.009	0.004	83	26	14

Particle trajectories obtained by the simulation were analyzed in the same manner as the experimentally-obtained single-molecule trajectories (see Table S5 for parameter values of each state).

Table S8. List of notations for diffusion parameters

Analysis	Parameter	Unit	Equation No.	Meaning
MSD	D_M	μm²/s	(2)	Diffusion coefficient obtained from MSD of the entire trajectory data
	ε_M	nm		Localization error obtained from MSD of the entire trajectory data
PDF	$D_{PDF\text{-}state}$	μm²/s	(3)	Diffusion coefficient for each diffusion state estimated by MLE
	$p_{PDF\text{-}state}$	%		Ratio of each diffusion state estimated by MLE
HMM	$D_{HMM\text{-}state}$	μm²/s	(6)	Diffusion coefficient for each diffusion state estimated by HMM
	$p_{HMM}0_{state}$	%		Initial probability of each diffusion state for each trajectory estimated by HMM
	$p_{S\text{-}state}$	%		Steady state ratio for each diffusion state obtained by averaging the probability of diffusion state at each time for each trajectory estimated by HMM
MSD after state estimation by HMM	$D_{S\text{-}state}$	μm²/s	(7)	Diffusion coefficient obtained from MSD of each diffusion state estimated by HMM
	$\varepsilon_{S\text{-}state}$	nm		Localization error obtained from MSD of each diffusion state estimated by HMM

Journal of Cell Science • Supplementary information

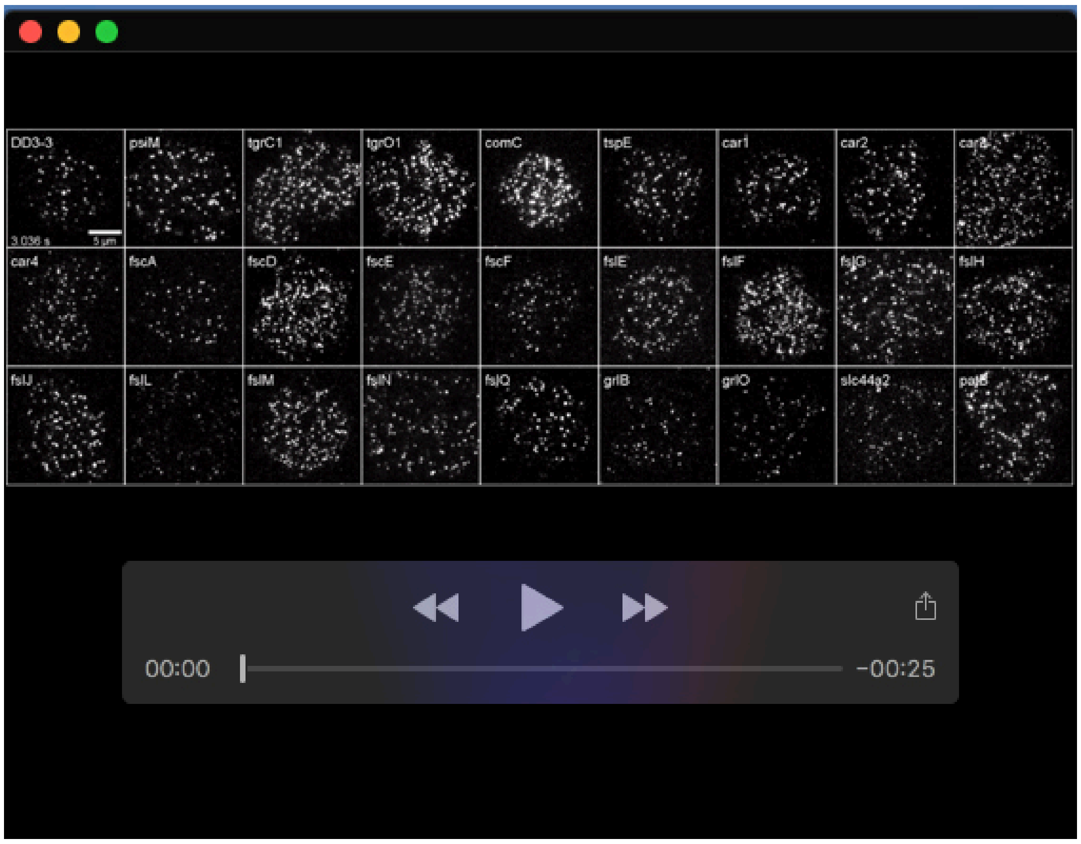

Movie 1. Single molecule imaging of the 27 transmembrane proteins in *Dictyostelium discoideum* cells by TIRFM. Scale bars, 5 μm. Time format is "s.fff".

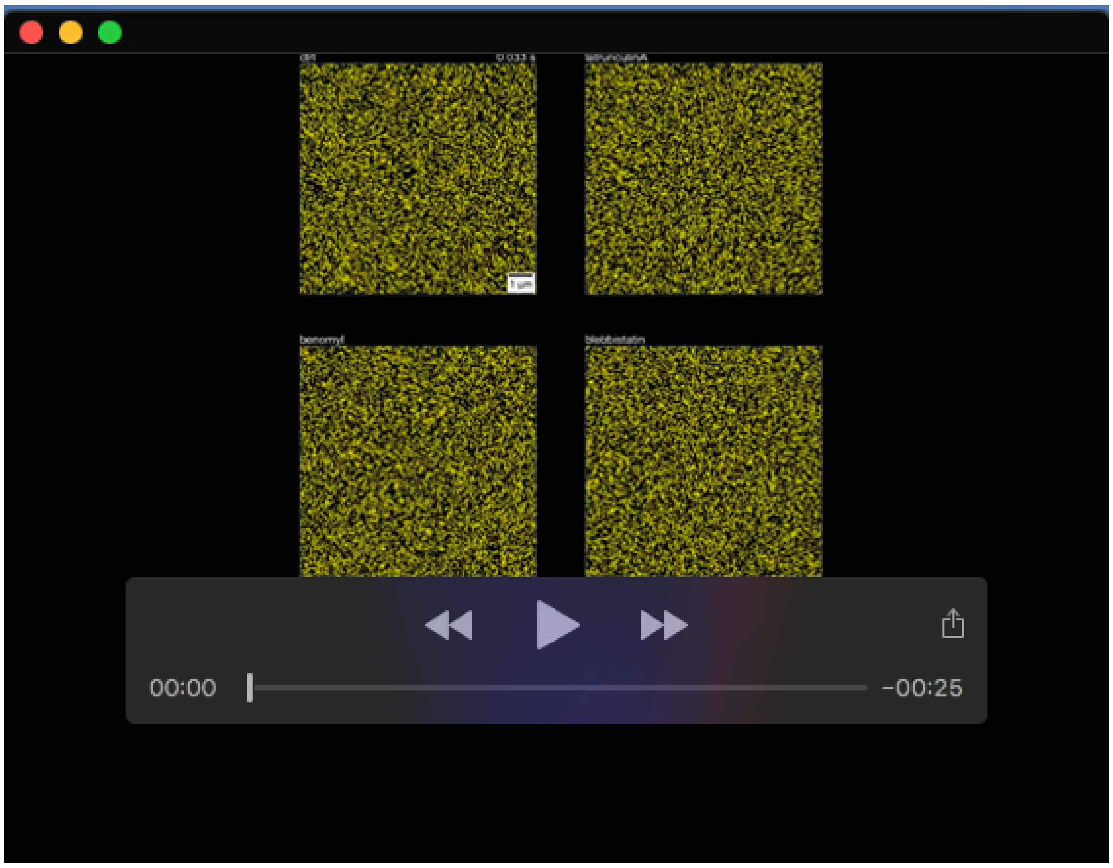

Movie 2. Simulation of particle diffusion in field models under control condition. Scale bars, 1 μm. Time format is "s.fff".

Journal of Cell Science • Supplementary information
