# Supplementary material for: Field model for multistate lateral diffusion of various transmembrane proteins observed in living Dictyostelium cells
Source: J Cell Sci. 2023 Feb 20;136(4):jcs260280. doi: 10.1242/jcs.260280 (PMC10022678; doi:10.1242/jcs.260280)
Supplement: Supplementary file 1 [file joces-136-260280_review_history.pdf]

Journal of Cell Science

Field model for multistate lateral diffusion of various transmembrane proteins observed in living *Dictyostelium* cells

Kazutoshi Takebayashi, Yoichiro Kamimura and Masahiro Ueda

DOI: 10.1242/jcs.260280

Editor: David Stephens

Review timeline

Original submission:	24 May 2022
Editorial decision:	12 July 2022
First revision received:	14 December 2022
Accepted:	11 January 2023

Original submission

First decision letter

MS ID#: JOCES/2022/260280

MS TITLE: Field model for multistate lateral diffusion of various transmembrane proteins in living Dictyostelium cells

AUTHORS: Kazutoshi Takebayashi, Yoichiro Kamimura, and Masahiro Ueda
ARTICLE TYPE: Short Report

We have now reached a decision on the above manuscript.

To see the reviewers' reports and a copy of this decision letter, please go to: https://submit-jcs.biologists.org and click on the 'Manuscripts with Decisions' queue in the Author Area. (Corresponding author only has access to reviews.)

As you will see, the reviewers raise a number of substantial criticisms that prevent me from accepting the paper at this stage. They suggest, however, that a revised version might prove acceptable, if you can address their concerns. I consider the comments quite clear and most reflecting concerns on statistical validation. If you think that you can deal satisfactorily with the criticisms on revision, I would be pleased to see a revised manuscript. We would then return it to the reviewers.

Please ensure that you clearly highlight all changes made in the revised manuscript. Please avoid using 'Tracked changes' in Word files as these are lost in PDF conversion.

I should be grateful if you would also provide a point-by-point response detailing how you have dealt with the points raised by the reviewers in the 'Response to Reviewers' box. Please attend to all of the reviewers' comments. If you do not agree with any of their criticisms or suggestions please explain clearly why this is so.

Reviewer 1

Advance summary and potential significance to field

© 2023. Published by The Company of Biologists under the terms of the Creative Commons Attribution License (https://creativecommons.org/licenses/by/4.0/).

SPT-TIRF study of membrane protein mobility on live Dictyostelium. Multistate diffusion is found to be a good model for protein mobility. Good data analysis and linkage to a possible mechanistic simulation model that provides a possible explanation of the findings.

Comments for the author

I think that this is a very good study of protein diffusion in a live cell membrane. I have only fairly minor comments, but I encourage the authors to address these points as other biophysicists are likely to ask similar questions.

1. On page 4, in the brief description of methods, it would be helpful to give the time interval for imaging (30 Hz I think) and some statistics on the track durations (mean and deviation, or even better show a histogram of the track durations measured in frames).

2. Page 5 line 11-12. It is quite surprising that the diffusion coefficients get smaller upon cytoskeletal or micro tubular disruption, especially relative to mammalian cell studies where often the opposite is true. Can the authors propose a mechanism here and explain the differences?

3. Page 5 line 21, TIRFM / TIFRM

4. Page 5 line 26 and methods Eq 3 (page 13). It is not very satisfactory to use the mixture Gaussian / AIC approach to estimate the number of diffusive states and then impose this number of states on the HMM. Two possible alternatives: (i) use AIC with HMM fits for different-sized HMMs. (ii) work directly with the HMM in a Bayesian framework that allows you to select the model along with the parameters (e.g. Falcao, Physical Biology 2020, DOI:10.1088/1478-3975/ab64b3).

5. Page 8. The proposed field model is reasonably consistent with the experimental findings and I have no issue with the model. But I wonder if a model where the tracked proteins can bind and unbind with various binding partners, with reduced mobility upon binding, in a spatially-independent way, would also fit the data. Certainly, it would be consistent with the literal HMM model that is used for data analysis. To show that the proposed spatial model is better, would require interrogation of spatial properties of the tracks. I think that the authors should comment on this issue and explain the pros and cons of their proposed spatial model vs the HMM model itself (which is spatially independent).

6. Methods, page 12 line 37, Eq. 2. What were the fit values for epsilon? These should be reported and compared to independent estimates for precision of the imaging-tracking system.

7. Methods, HMM, Page 13, and relevant results sections. The HMM does not include localization errors? Eq 2 allows for errors in the MSD analysis but there is no mention of errors in the HMM. This should be explained, at least.

8. Methods, HMM, Page 13, Table S3, and page 5. Errors in diffusion coefficients should be given. The HMM parameters have errors on an experiment-wise basis, see for example Das et al PLoS Comp Biol, 5(11):e1000556 DOI:10.1371/journal.pcbi.1000556. On page 5, line 11, relative changes in diffusivities are reported with errors but I am not sure what those are.

9. Methods, HMM, page 14. It makes no sense to segment the tracks into the different HMM states, generate histograms and fit an exponential model, in order to generate the state lifetimes. The state lifetimes can be obtained from the HMM transition matrix. It might be interesting to look for outliers by generating the histogram from data and comparing to the HMM result, but I think that should be a secondary analysis.

<u>Reviewer 2</u>

Advance summary and potential significance to field

Important study, but lack of statistical validation.

© 2023. Published by The Company of Biologists under the terms of the Creative Commons Attribution License (https://creativecommons.org/licenses/by/4.0/).

Comments for the author

The manuscript by Takebayashi describes experiments on the mobility of 27 membrane proteins in Dictyostelium cells. The authors measure the tracks of single molecules, deduce from the probability density function that diffusion has three components, slow, middle and fast. Using a hidden Markov model the authors propose that the three components are three diffusion states with transitions during the tracks. Data are interpreted as free diffusion in three membrane regions of different viscosities. With life-time estimates and diffusion rate constants the authors estimate the size of these regions. They investigate the role of the cytoskeleton using several drugs affecting actin-myosin and microtubules.

This is a very interesting study with many different membrane proteins and different drugs affecting the cytoskeleton. Below several points are listed that should be addressed. The main concern is the complete lack in the statistical analysis of the confidence, both of the alternative models and the deduced parameters. In any Science that uses large datasets for model discrimination and parameter fitting, the deduced models are only relevant if they are supported by a discussion on the uncertainties of the model and the deduced parameters. In the absence of a solid statistical analysis on these uncertainties, the deduced model and its parameter values cannot be evaluated.

1. The data analysis, model discrimination, parameter fitting, and deduced three membrane regions all have uncertainties that accumulate in the final model. These uncertainties must be addressed. Below the errors/uncertainties are listed in the sequence of the analysis in the manuscript.

   a. Determination of light source indicating the position of the protein. The light spot has a size of around 400 nm; calculating the position of the light source with point spread function has an error. For instance Latek et al (Single molecule localization microscopy, 2021, Nature Reviews) mention a precision of 10 to 20 nm. Assuming this precision leads to a SD=10 nm for spot determination, the measured step size Δr has SD=14 nm.

   b. The measured step sizes Δr are binned in a histogram (e.g. Fig 2A). The bin size is probably 3 nm (see point 6). The number of observations in a bin has errors due to a systematic error and two stochastic errors. The systematic error is due to the error of Δr mentioned above; assume that the real step size is 1 nm, then the SD=10 nm for the start and end points of that step leads to an expected average step size of about 5 nm. This systematic error influences the first few bins (up to about Δr =20 nm) and leads to a lower number of observations in the very small Δr bins and therefore to a larger fitted diffusion rate constant of the slowest component. The two sources of stochastic error in the number of observations per bin are first the allocation of a step size to a specific bin, because the error in step size may be as large as 14 nm, while bin size is only 3 nm. The second stochastic error is due to the limited number of observations in a specific bin. If the number of observations in a bin is N, then the SD in that bin scales with the square root of N. This becomes especially relevant at ΔR>0.1 μm for the identification of the fast component; based on 80,000 observations and the deduced parameters for p and D for car1, it is estimated that bins >0.1 μm contain only about 10 observations with a large uncertainty.

   c. The values for the diffusion parameter p and D are estimated using equation (3). Since the data (number of observation per bin) have an uncertainty, the parameter values that best fit the data are also uncertain. This is especially relevant for the proposed three component model where as many as five parameters have to be fitted. It should be noted that some of these five parameters are associated in the fitting: for instance increasing the Diffusion rate constant D of the middle component is compensated for in the fit by decreasing the proportion of the fast component or decreasing the diffusion rate constant of the fast component. Fitting uses the non-linear equation (3), so determining the 95% confidence interval of the parameters is not trivial. Probably the best method is by bootstrap analysis recreating many PDF data sets and fitting these datasets. Based on the low number of observations in the bins ΔR>0.1 μm describing the fast component, it is expected that the optimal value for the diffusion rate constant of the fast component is very uncertain.

© 2023. Published by The Company of Biologists under the terms of the Creative Commons Attribution License (https://creativecommons.org/licenses/by/4.0/).

d. The AIC is used for model discrimination. Since the source data for the fit has errors, also the calculated AIC value is uncertain with a 95% confidence interval. The suggested bootstrap analysis described above will show how often three components have the lowest AIC value. It should be noted that even when the model with three components has the lowest AIC value - and is thus the preferred model- this conclusion is not absolute. The strength of this conclusion is given by calculating the relative likelihood of the model relative to another model. For the model with three versus two components the relative likelihood is given by $\exp((AIC_3 - AIC_2)/2)$.

e. The conclusion that the PDF is described best by three components can be explained by two models. First the protein can be "permanently" in one of three states (for instance fast diffusing truncated protein, middle diffusion monomer, and slow diffusing aggregate), or second the protein can fluctuate between three different states as is assumed in the manuscript. No evidence is provided for rejecting the first possibility.

f. In the HMM model, sections of the tracks are attributed to the slow, middle and fast component. This attribution forms the basis for estimating the diffusion rate constant, the ratio, and the life time of the three states. However, the attribution of a step to one of these states is not absolute but is a probability; for instance, Δr steps around 0.08 μm are equally likely to be part of the slow and middle component. Therefore, the transition-time/step between two components in a cell track can not be determined precisely but has an uncertainty of several steps. This uncertainty effects both the parameters of the diffusion components (D and p), as well as the length of the track in a certain state.
Another problem is the following: Recordings are 100 images at 33 ms interval. The method section describes per condition ~3,000 single molecule tracks providing ~80,000 displacements. This suggests that a track on average has only 26 displacements (during 0.88s), probably with a wide range in length. The presence of short tracks and the variation in track length is expected to complicate the analysis of transitions and the life- times, especially of the slow component.

g. Fitting the life time in Figure 3 gives the optimal parameter with a 95% confidence interval.

h. The deduced size of membrane patches for the three component has a 95% confidence interval due to the error in life time and error in diffusion rate constant.

i. The deduced viscosity of the three membrane areas has a 95% confidence interval.

2. The manuscript heavily depends on the evidence for three components. The AIC values for one, two, three and four components must be presented as supplemental data, including uncertainties as described in point 1).

3. The connection between equation 3 and Figure 2A is confusing. Equation 3 is a probability density function with dimension 1/μm. Figure 2A is a histogram with occurrence or probability and is dimension-less. So the experimental data of Fig 2A are divided by the bin size and then fitted using equation (3). This should be explained.

4. Figure 2A needs further clarification. It shows experimental data (green) in bins; these data are observations and thus fraction or occurrence, not probability. The lines are the calculated probability.
The dashed lines in Figure 2A are not explained in the legend.
Many readers will ask how the curve for two components will look like. As drawn, the curve is probably indistinguishable from the curve for three components, but may become distinguishable when the experimental data and the curves at $\Delta R > 0.1$ μm are enlarged in an inset.

5. Indicate the pixel size in nm in line 46 on page 11.

6. Indicate the bin size in nm in line 4 on page 13 where the histogram is introduced.
7. From the information in the title and the abstract it is not immediately clear to the reader

© 2023. Published by The Company of Biologists under the terms of the Creative Commons Attribution License (https://creativecommons.org/licenses/by/4.0/).

that the manuscript has experimental data and that the model is based on these data. This can be easily overcome by mentioning somewhere “experimental data” or “observed” single molecule tracks, etc.

8. The HMM model a priori assumes three components. Given that the fast component in the PDF has low abundance (~2%), the presence of the fast component in cell tracks is very rare. This is also noticed by the very short life-time of the fast component, suggesting that a fast section in a track has only a few steps. What is the evidence that the HMM model must have three components? How does the HMM model fit the data when only two components are used?

9. The transition between fast and slow component has not been observed. This is strange, and one of the arguments to have some doubt on the presence of a fast component, together with the calculated low abundance and low confidence of the fitted parameters. The only evidence for a fast component comes from the AIC of the PDF. Are more arguments with statistical support available?

10. The field model in figure 4 and 7S is a nice attempt to summarize the data. However, it is strange to see squares for membrane patches; they are thermodynamically impossible. Can they be changed to circles?

11. On page 13 the same symbol “capital P” is used for the probability density function in equation (3) and log likelihood in equation (4). Very confusing.

12. Estimates for diffusion rate constants D and relative fraction p of the tree components were obtained twice, from fitting PDF and with the HMM. Present both datasets including 95% confidential interval in the supplemental material for all proteins.

13. The possible role of the cytoskeleton has been extensively studied and comprises a very large effort. The discussion on these data and how the cytoskeleton may affect diffusion is very limited. Is there more to say? Are among the 27 proteins membrane proteins known to interact with the cytoskeleton, e.g. connecting the plasma membrane with the F-actin cortex?

First revision

Author response to reviewers' comments

Our response to Reviewer #1:

We thank Reviewer #1 for the valuable suggestions that helped improve our paper. The reviewer’s comments are provided in ***italics***, and our responses are provided below. Our changes in the revised manuscript are marked with **GREEN-colored font**. The expression ‘page XX, lines XX-XX’ in our responses means the location of the changes in the manuscript.

We have changed the notation of the diffusion coefficient (D), ratio (p) and localization error (ε), which are summarized in Table S8 of the revised manuscript.

Advance Summary and Potential Significance to Field:

SPT-TIRF study of membrane protein mobility on live Dictyostelium. Multistate diffusion is found to be a good model for protein mobility. Good data analysis and linkage to a possible mechanistic simulation model that provides a possible explanation of the findings.

Comments for the Author:

I think that this is a very good study of protein diffusion in a live cell membrane. I have only fairly *minor comments, but I encourage the authors to address these points as other biophysicists are likely to ask similar questions.*

Our reply: We thank Reviewer #1 for the positive and supportive comments. As described in the following responses, we have taken all comments into account in the revised version.

© 2023. Published by The Company of Biologists under the terms of the Creative Commons Attribution License (https://creativecommons.org/licenses/by/4.0/).

Comment #1: *On page 4, in the brief description of methods, it would be helpful to give the time interval for imaging (30 Hz I think) and some statistics on the track durations (mean and deviation, or even better show a histogram of the track durations measured in frames).*

Our reply: Following the reviewer's suggestion, we have included the time interval for imaging (30 frames/sec) in the main text of the revised manuscript (page 4, line 16).

To show the histogram of the track durations, we have added Fig. S7 in the revised manuscript. The mean and deviation of the track durations were 23.9±28.9 frames (Fig. S7A) and durations longer than 1 second constituted more than 70% of the total data (Fig. S7B). Please see Methods "Tracking of fluorescence signals" for more details (page 11, lines 23-28).

Reviewer #2, Comment #1-f is related to this comment. As described in our reply to that comment, the diffusion parameters can be estimated even in the presence of trajectory interruptions. We have added Fig. S7C-E in the revised manuscript to show the effects of trajectory interruption on the estimation of the diffusion properties. Please also see our response to that comment.

Comment #2: *Page 5 line 11-12. It is quite surprising that the diffusion coefficients get smaller upon cytoskeletal or micro tubular disruption, especially relative to mammalian cell studies where often the opposite is true. Can the authors propose a mechanism here and explain the differences?*
Our reply: We thank Reviewer #1 for the comment. We think this is one of the characteristic features in the differences between mammalian cells and *Dictyostelium* cells with respect to membrane protein diffusion. We speculate that there are at least three possible mechanisms by which the cytoskeleton affects the plasma membrane. One is a cytoskeleton fence against the diffusion of membrane proteins, which is well known in mammalian cells. Another is vesicular transport to the plasma membrane, which would modulate the composition of lipids and other molecules in the membrane. Lastly, force generation by the cytoskeleton might affect viscosity of the plasma membrane mechanically. Currently, we have no evidence to distinguish these possibilities, but the reduced diffusion by inhibition of the cytoskeleton suggests that cytoskeletal fences do not play a major role in the diffusion properties of membrane proteins in *Dictyostelium* cells.

Due to the length limit of a Short Report, we only briefly discuss the cytoskeletal fence in the text (page 5, lines 14-15). Reviewer #2, **Comment #13** is related to this comment.

Comment #3: *Page 5 line 21, TIRFM / TIFRM*
Our reply: We thank Reviewer #1 for the comment. We have corrected the typo in the revised manuscript (page 5, line 19).

Comment #4: *Page 5 line 26 and methods Eq 3 (page 13). It is not very satisfactory to use the mixture Gaussian / AIC approach to estimate the number of diffusive states and then impose this number of states on the HMM. Two possible alternatives: (i) use AIC with HMM fits for different-sized HMMs. (ii) work directly with the HMM in a Bayesian framework that allows you to select the model along with the parameters (e.g. Falcao, Physical Biology 2020, DOI:10.1088/1478-3975/ab64b3).*
Our reply: We thank Reviewer #1 for the suggestions of the two alternative methods to estimate the number of diffusion states. We agree with Reviewer #1 that method (i) may be useful in this analysis. To obtain the value of AIC and its variation, we performed PDF and HMM analyses 10 times, each with different initial values for the measurement dataset of the membrane protein car1. The results are represented in the following table, in which AIC_{PDF} and AIC_{HMM} represents the AIC values obtained by the PDF and HMM analyses, respectively.

Number of diffusion states	AIC_{PDF}		AIC_{HMM}	
	Mean	95%CI	Mean	95%CI
1	-377761.7	4.38915E-11	-754567.7	8.7783E-11
2	-397264.2	2.08899E-06	-18437492.0	0.039745909
3	-398441.9	3.64431E-06	-255633413.9	5636494.2
4	-398394.0	8.50392E-06	-225968836.7	413920.7

© 2023. Published by The Company of Biologists under the terms of the Creative Commons Attribution License (https://creativecommons.org/licenses/by/4.0/).

As shown here, the three-state model had the lowest AIC value in both analyses. These results were the same for all other proteins analyzed. However, in the HMM analysis, especially for more than three states, the AIC values varied widely from analysis to analysis (please see 95% CI in above Table), and the calculations took a very long time before the log likelihood/AIC converged. For these reasons we decided to determine the number of diffusion states by the PDF and AIC analysis as before.

Also, we agree with Reviewer #1 that method (ii) may be useful. However, it is necessary to process the huge amount of single-molecule trajectory data that we have obtained experimentally to perform the calculations including the estimation of the number of diffusion states using the method (ii). In addition, as explained below in our rely to **Comment #7**, the localization errors of single molecules vary depending on the diffusion state, and thus the estimation of the localization errors should be included in method (ii). We believe that any analysis that estimates more parameters than the number of parameters in the HMM analysis we currently use requires more careful consideration. Furthermore, due to limitations in computational resources and machine power, the estimation using method (ii) is not feasible for our current circumstances. For these reasons, we did not apply method (ii) in this revision.

Because of these results, we decided to use the method described in the original manuscript, in which the number of diffusion states was estimated from the PDF/AIC analysis and applied to the HMM analysis to determine parameters such as the diffusion coefficients ($D_{\text{HMM-}\mathit{state}}$), initial probabilities and transition probability matrices.

To explain the PDF/AIC analysis more clearly, we have added more description in the Methods section of the revised manuscript (pages 12, lines 4-38).

Comment #5: *Page 8. The proposed field model is reasonably consistent with the experimental findings and I have no issue with the model. But I wonder if a model where the tracked proteins can bind and unbind with various binding partners, with reduced mobility upon binding, in a spatially- independent way, would also fit the data. Certainly, it would be consistent with the literal HMM model that is used for data analysis. To show that the proposed spatial model is better, would require interrogation of spatial properties of the tracks. I think that the authors should comment on this issue and explain the pros and cons of their proposed spatial model vs the HMM model itself (which is spatially independent).*

Our reply: We thank Reviewer #1 for the valuable comment. As pointed out by the reviewer, the HMM itself is spatially independent. The field model was introduced to explain why all 27 membrane proteins analyzed exhibited similar diffusion dynamics even though they are structurally different from each other. Probably, all these proteins have different binding partners from each other. Among those binding partners, lipids are most likely to affect the diffusion of the membrane proteins, in which lipids can form microdomains with different viscosities. By incorporating microdomains with different viscosities, our proposed field model can explain the experimental observations consistently.

As recommended by Reviewer #1, we have added the comments that the HMM itself is spatially independent and that further analysis of the spatial characteristics of single-molecule trajectories will provide insight into the microdomains (page 8, lines 21-24) to the revised manuscript.

Comment #6: *Methods, page 12 line 37, Eq. 2. What were the fit values for epsilon? These should be reported and compared to independent estimates for precision of the imaging-tracking system.*

Our reply: We thank Reviewer #1 for the valuable comment. We have included the values of epsilon in the Methods (page 11, line 46 – page 12, line 3), Table S2, Table S3, and Table S5 of the revised manuscript.

Reviewer #2, **Comment #1-a** is related to this comment. Please also see our response to that comment.

Comment #7: *Methods, HMM, Page 13, and relevant results sections. The HMM does not include localization errors? Eq 2 allows for errors in the MSD analysis but there is no mention of errors in the HMM. This should be explained, at least.*

Our reply: As pointed out by Reviewer #1, we did not include localization errors in the HMM analysis. In the early stages of this study, we attempted to estimate diffusion parameters and also the localization errors as unknown parameters in the HMM analysis, but were not successful. We think this may be due to the fact that the localization errors vary significantly with multiple diffusion states, as explained below.

© 2023. Published by The Company of Biologists under the terms of the Creative Commons Attribution License (https://creativecommons.org/licenses/by/4.0/).

In our current HMM analysis, the localization errors are not included in Eq. (6) of the revised manuscript, and thus the diffusion coefficients ($D_{HMM\text{-}state}$) are the apparent diffusion coefficients for each state. We use $D_{HMM\text{-}state}$ only to estimate the diffusion states along single-molecule trajectories. After assigned into three diffusion states along the trajectories by the HMM, the obtained trajectories for each state are used to calculate the MSD using Eq. (7), from which we can obtain the diffusion coefficients $D_{S\text{-}state}$ with the corresponding localization error $\varepsilon_{S\text{-}state}$ for each state. Considering that the fluorescent spots of single molecules are obtained by the accumulation of fluorescence over a period of 33 msec, it is reasonable that the error varies depending on the diffusion state. In fact, the localization errors differed depending on the different diffusion states, in which the fast diffusion state showed relatively large values of error (~90 nm), while the slow diffusion showed conversely small values of error (~10 nm), as shown in Tables S3 and S5. These error values were only obtained after the trajectories were divided for the HMM analysis and analyzed using the MSD, so they cannot be included a priori in the HMM analysis. The HMM analysis did not work well when the error values were estimated as unknown parameters.

In order to see whether the HMM can estimate the diffusion state along trajectories for which the localization error of the fluorescent spots varies depending on the diffusion state, the particle trajectories generated by the numerical simulation were analyzed using the HMM (Fig. S8A- C). We confirmed that our HMM analysis without the localization errors can estimate successfully diffusion parameters, such as $D_{S\text{-}state}$, with the corresponding localization error $\varepsilon_{S\text{-}state}$ from single molecule trajectories that were generated by the numerical simulation with localization errors taken into account (Fig. S8B).

To describe the HMM analysis more clearly and to show the results, we have added more explanation of the HMM in the Methods section (pages 13, lines 34 – page 14, line 13) and added Fig. S8.

Comment #8: *Methods, HMM, Page 13, Table S3, and page 5. Errors in diffusion coefficients should be given. The HMM parameters have errors on an experiment-wise basis, see for example Das et al PLoS Comp Biol, 5(11):e1000556 DOI:10.1371/journal.pcbi.1000556. On page 5, line 11, relative changes in diffusivities are reported with errors but I am not sure what those are.*
Our reply: We thank Reviewer #1 for the valuable comment. We have included the errors in new Table S2, Table S3, Table S4, Table S5, Table S6 and Fig. S4 of the revised manuscript.

Concerning the relative changes in diffusion on page 5 line 11 of the original manuscript, we showed the relative change in the diffusion coefficients of the 27 different proteins due to drug treatment as means and SD. Since the values may be confusing to the reader and there is a length limit for a Short Report, we deleted these data from the main text of the revised manuscript and instead added Table S2.

Comment #9: *Methods, HMM, page 14. It makes no sense to segment the tracks into the different HMM states, generate histograms and fit an exponential model, in order to generate the state lifetimes. The state lifetimes can be obtained from the HMM transition matrix. It might be interesting to look for outliers by generating the histogram from data and comparing to the HMM result, but I think that should be a secondary analysis.*
Our reply: We agree with the reviewer's comments. In fact, the lifetime values obtained from the histograms of the single-molecule trajectories for different diffusion states were in good agreement with the values obtained using the HMM transition matrix. But such agreement was not obvious to us before we analyzed the experimental trajectory data. Since the two methods for determining lifetimes may yield different results, we decided to analyze the lifetime histogram in the revised manuscript.

Our response to Reviewer #2:

We are grateful to Reviewer #2 for the critical comments and useful suggestions that have helped us improve our paper. As indicated in our responses, we have addressed all comments and suggestions into the revised version of our manuscript. We put a summary of the comments made by the reviewer in ***italics***. Our changes in the revised manuscript are marked with **GREEN-colored font**. The expression ‘page XX, lines XX-XX’ in our response means the location of the changes in the manuscript.

© 2023. Published by The Company of Biologists under the terms of the Creative Commons Attribution License (https://creativecommons.org/licenses/by/4.0/).

We have changed the notation of the diffusion coefficient (D), ratio (p) and localization error (ε), which are summarized in Table S8 of the revised manuscript.

Advance Summary and Potential Significance to Field:
The manuscript by Takebayashi describes experiments on the mobility of 27 membrane proteins in Dictyostelium cells. The authors measure the tracks of single molecules, deduce from the probability density function that diffusion has three components, slow, middle and fast. Using a hidden Markov model the authors propose that the three components are three diffusion states with transitions during the tracks. Data are interpreted as free diffusion in three membrane regions of different viscosities. With life-time estimates and diffusion rate constants the authors estimate the size of these regions. They investigate the role of the cytoskeleton using several drugs affecting actin-myosin and microtubules.

This is a very interesting study with many different membrane proteins and different drugs affecting the cytoskeleton. Below several points are listed that should be addressed. The main concern is the complete lack in the statistical analysis of the confidence, both of the alternative models and the deduced parameters. In any Science that uses large datasets for model discrimination and parameter fitting, the deduced models are only relevant if they are supported by a discussion on the uncertainties of the model and the deduced parameters. In the absence of a solid statistical analysis on these uncertainties, the deduced model and its parameter values cannot be evaluated.

Our reply: We thank the reviewer for the positive and supportive comments. As described in the following responses, we have taken all comments into account in the revised version.

Comment #1: *The data analysis, model discrimination, parameter fitting, and deduced three membrane regions all have uncertainties that accumulate in the final model. These uncertainties must be addressed. Below the errors/uncertainties are listed in the sequence of the analysis in the manuscript.*

Comment #1-a: *Determination of light source indicating the position of the protein. The light spot has a size of around 400 nm; calculating the position of the light source with point spread function has an error. For instance, Latek et al (Single molecule localization microscopy, 2021, Nature Reviews) mention a precision of 10 to 20 nm. Assuming this precision leads to a SD=10 nm for spot determination, the measured step size Δr has SD=14 nm.*

Our reply: Reviewer #2 asks we show the localization errors or accuracy of the single molecule detection. For the immobilized fluorescent dyes, the localization accuracy is about 10 to 20 nm, as indicated in the reviewer's citation. In our study, for the diffusion analysis, the localization errors became larger than those for the immobilized fluorescent spots because of the movements. To estimate the localization errors for diffusing molecules, we determined ε using Eq. (2),

$$\mathrm{MSD} = 4\mathrm{D_M}\Delta\mathrm{t} + 4\varepsilon_\mathrm{M}^2 \qquad \mathrm{Eq.}\,(2)$$

, where D_M and ε_M represent the diffusion coefficient and the localization error of the molecule, respectively. Epsilon can be obtained from value of the intercept. In the case of our TIRFM setup, epsilon was 12-28 nm when not dividing the data into 3 diffusion states (see Table S2 of the revised manuscript).

During this revision process, it became clear that localization errors of single molecules vary depending on the diffusion state. After assigned into three diffusion states along the single-molecule trajectories by the HMM, the obtained trajectories for each state were used to calculate the MSD, from which we obtained the diffusion coefficients and the corresponding localization errors for each state. The fast diffusion state showed relatively larger errors, while slow diffusion showed smaller values. Typically, the fast, middle and slow diffusion states were 88±5, 11±5, and 10±2 nm for the epsilon values of membrane protein car1, respectively (Table S3). Because the fluorescent spots of a single molecule are obtained by the accumulation of fluorescence over a period of 33 msec, it is reasonable that the localization errors varied depending on the diffusion state.

We have included the values of epsilon in the Methods (page 11, line 46 and page 13, line 40-43) and Table S2, Table S3, and Table S5 of the revised manuscript.

Comment #1-b: *The measured step sizes Δr are binned in a histogram (e.g. Fig 2A). The bin size is probably 3 nm (see point 6). The number of observations in a bin has errors due to a systematic*

© 2023. Published by The Company of Biologists under the terms of the Creative Commons Attribution License (https://creativecommons.org/licenses/by/4.0/).

error and two stochastic errors. The systematic error is due to the error of Δr mentioned above; assume that the real step size is 1 nm, then the SD=10 nm for the start and end points of that step leads to an expected average step size of about 5 nm. This systematic error influences the first few bins (up to about Δr =20 nm) and leads to a lower number of observations in the very small Δr bins and therefore to a larger fitted diffusion rate constant of the slowest component. The two sources of stochastic error in the number of observations per bin are first the allocation of a step size to a specific bin, because the error in step size may be as large as 14 nm, while bin size is only 3 nm. The second stochastic error is due to the limited number of observations in a specific bin. If the number of observations in a bin is N, then the SD in that bin scales with the square root of N. This becomes especially relevant at ΔR>0.1 μm for the identification of the fast component; based on 80,000 observations and the deduced parameters for p and D for car1, it is estimated that bins >0.1 μm contain only about 10 observations with a large uncertainty.

Our reply: We thank the Reviewer #2 for the valuable comment. As the reviewer points out, there are various concerns when determining the parameters of diffusion using histograms and fitting, since the shape of the histogram varies depending on the bin size setting. Therefore, we determined the parameters without using histograms. As described in the original paper, we used the maximum likelihood estimation (MLE) to determine the diffusion coefficient D_{PDF} and the ratio p_{PDF}, where the parameters were determined using all Δr data without setting the bin size.

We have added more detail about the PDF analysis with the MLE in the Methods (page 12, line 4-38).

In reflection to this comment, we think Fig. 2A in the original manuscript and the corresponding explanation was confusing to the reader. Fig. 2A was not a fitting of the histogram of displacements (Δr) by the PDF, but the histogram overlaid with the PDF (Eq. (3)) based on the parameters estimated by the MLE. To avoid possible confusion in our original description, we have added Fig. S2 in the revised manuscript.

We thank Reviewer #2 once again for this comment, which has improved our manuscript.

Comment #1-c: *The values for the diffusion parameter p and D are estimated using equation (3). Since the data (number of observation per bin) have an uncertainty, the parameter values that best fit the data are also uncertain. This is especially relevant for the proposed three component model where as many as five parameters have to be fitted. It should be noted that some of these five parameters are associated in the fitting: for instance increasing the Diffusion rate constant D of the middle component is compensated for in the fit by decreasing the proportion of the fast component or decreasing the diffusion rate constant of the fast component. Fitting uses the non-linear equation (3), so determining the 95% confidence interval of the parameters is not trivial. Probably the best method is by bootstrap analysis recreating many PDF data sets and fitting these datasets. Based on the low number of observations in the bins ΔR>0.1 μm describing the fast component, it is expected that the optimal value for the diffusion rate constant of the fast component is very uncertain.*

Our reply: We thank the Reviewer #2 for the valuable comment. Following the reviewer's suggestion, we performed the bootstrap analysis to investigate the certainty of the estimated parameters. The bootstrap method was used to generate 10 displacement Δr datasets to determine the diffusion parameters by the MLE. For example, the estimated D_{PDF} and p_{PDF} for the 3-state model of car1 under drug-untreated conditions were (mean±95% CI):

Diffusion states	D_{PDF} (μm^2/s)	p_{PDF} (%)
slow	0.017±0.000	58.2±2.2
middle	0.045±0.001	38.9±2.2
fast	0.294±0.009	2.3±0.1

We have added the explanation of the bootstrap analysis to the Methods (page 14, lines 21-36) of the revised manuscript and the results in Fig. S2A, B, C for the one-, two- and three-component models, respectively.

© 2023. Published by The Company of Biologists under the terms of the Creative Commons Attribution License (https://creativecommons.org/licenses/by/4.0/).

Comment #1-d: *The AIC is used for model discrimination. Since the source data for the fit has errors, also the calculated AIC value is uncertain with a 95% confidence interval. The suggested bootstrap analysis described above will show how often three components have the lowest AIC value. It should be noted that even when the model with three components has the lowest AIC value -and is thus the preferred model- this conclusion is not absolute. The strength of this conclusion is given by calculating the relative likelihood of the model relative to another model. For the model with three versus two components the relative likelihood is given by exp((AIC3 – AIC2)/2).*
Our reply: We thank Reviewer #2 for the valuable comment. Following the reviewer's suggestion, we performed a bootstrap analysis to obtain the AIC values as described in our reply to **Comment #1-c**. We have added the results in Fig. S2D of the revised manuscript. We confirmed that the three- component model is the most likely for the experimental data.

Comment #1-e: *The conclusion that the PDF is described best by three components can be explained by two models. First the protein can be "permanently" in one of three states (for instance fast diffusing truncated protein, middle diffusion monomer, and slow diffusing aggregate), or second the protein can fluctuate between three different states as is assumed in the manuscript. No evidence is provided for rejecting the first possibility.*
Our reply: As the reviewer points out, the PDF/AIC analysis itself can reveal that there are three states but cannot reveal whether there are state transitions or not. Therefore, we used a hidden Markov model (HMM) to analyze the diffusion. As shown in Fig. S8D, E of the revised manuscript, the HMM analysis can detect and estimate the state transitions successfully for the single-molecule trajectory generated by the numerical simulation. When there are no state transitions, the estimated values of the transition probability matrix of the HMM became zero in most cases. On the other hand, when the simulations assume state transitions, the transition probabilities were correctly estimated. Thus, the results of the HMM analysis indicates state transitions exist.

We have added these results of the HMM analysis to the Methods (page 14, lines 14-19) and Fig. S8D, E.

Comment #1-f: *In the HMM model, sections of the tracks are attributed to the slow, middle and fast component. This attribution forms the basis for estimating the diffusion rate constant, the ratio, and the lifetime of the three states. However, the attribution of a step to one of these states is not absolute but is a probability; for instance, Δr steps around 0.08 μm are equally likely to be part of the slow and middle component. Therefore, the transition-time/step between two components in a cell track cannot be determined precisely but has an uncertainty of several steps. This uncertainty effects both the parameters of the diffusion components (D and p), as well as the length of the track in a certain state.*

Another problem is the following: Recordings are 100 images at 33 ms interval. The method section describes per condition ~3,000 single molecule tracks providing ~80,000 displacements. This suggests that a track on average has only 26 displacements (during 0.88s), probably with a wide range in length. The presence of short tracks and the variation in track length is expected to complicate the analysis of transitions and the life-times, especially of the slow component.
Our reply: To show the certainty of the diffusion parameters estimated by the HMM analysis, we performed a bootstrap analysis. We obtained 10 single-molecule trajectory datasets for each experimental condition. After assigning three mobility states along the trajectories by the HMM analysis, the obtained trajectories for each state were used to calculate the MSD and then the diffusion coefficients ($D_{S\text{-fast}}$, $D_{S\text{-middle}}$ and $D_{S\text{-slow}}$) with the localization errors ($\varepsilon_{S\text{-fast}}$, $\varepsilon_{S\text{-middle}}$ and $\varepsilon_{S\text{-slow}}$). The ratio ($p_{S\text{-fast}}$, $p_{S\text{-middle}}$ and $p_{S\text{-slow}}$) was obtained by the HMM analysis. From the 10 datasets, we determined the mean and 95% CI. For example, $D_{S\text{-}state}$ and $p_{S\text{-state}}$ for car1 under drug-untreated conditions were:

Diffusion states	$D_{S\text{-}state}$ (μm^2/s)	$\varepsilon_{S\text{-state}}$ (nm)	$p_{S\text{-state}}$ (%)
slow	0.013±0.001	10±2	42.3±2.7
middle	0.034±0.001	11±5	52.8±2.5
fast	0.071±0.013	88±5	4.0±0.3

© 2023. Published by The Company of Biologists under the terms of the Creative Commons Attribution License (https://creativecommons.org/licenses/by/4.0/).

The estimated values of the diffusion coefficients by the PDF analysis ($D_{PDF\text{-}state}$, Fig. S2C) were larger than those by the HMM analysis ($D_{S\text{-}state}$, Table S3). This is due to the fact that the PDF analysis did not include localization errors. Thus, the $D_{PDF\text{-}state}$ obtained from the PDF analysis is the apparent diffusion coefficient, which was used only to estimate the number of states.

The estimated diffusion parameters and 95% CI for all protein species under the control and drug-treated conditions are shown in new Fig. 2E,F,G and Table S3.

As for other concerns, the reviewer points out the uncertainty in the estimation derived from the shortness of the trajectory. As shown in new Fig. S7 of the revised manuscript, the single-molecule trajectories have a wide range in length (Fig. S7A), but most data points are derived from 100-frame long trajectories (Fig. S7B). Thus, single-molecule trajectories longer than 1 second occupy more than 70% of the total data (inset of Fig. S7B).

To investigate the effect of the short trajectories on the estimation of the diffusion parameters, we prepared single-molecule trajectories that were generated by the numerical simulation and cut according to the rules described in **Reproduction of tracking interruptions** of the Methods section (page 16), and applied the HMM analysis to the resulting trajectories. As shown in Fig. S7C-F, the estimated diffusion coefficients, ratios, and state transition probabilities were in good agreement with the simulation settings irrespective of whether there was trajectory interruption or not. Based on these results, we conclude that the diffusion parameters can be estimated even in the presence of a trajectory interruption. The probability to estimate the three diffusion states correctly by the HMM was about 80% (Fig. S7F).

Accordingly, we have added more explanation to the Method section (pages 11, line 23-28) and Fig. S7C-F in the revised manuscript.

Comment #1-g: *Fitting the lifetime in Figure 3 gives the optimal parameter with a 95% confidence interval.*
Our reply: We thank Reviewer #2 for the comment. Using the bootstrap analysis as described above, we determined the optimal parameters with 95% CI for the lifetime. We have added the results in Fig. 3B,D,F, Table S4 and Table S5 of the revised manuscript.

In addition, the mean and SD of the lifetime obtained from each of the 27 proteins are shown in new Fig. 3I,K of the revised manuscript.

Comment #1-h: *The deduced size of membrane patches for the three component has a 95% confidence interval due to the error in lifetime and error in diffusion rate constant.*
Our reply: As the reviewer points out, the size of the patches depends on the diffusion parameters, such as lifetime and diffusion coefficient, and thus the size would have some variation due to the error in the parameters. In the construction of the field model, the patch size is not estimated directly from the data, and thus we cannot calculate the 95% CI. Instead, we show the effects of the patch size on lifetimes by performing a numerical simulation of the particle diffusion in the field model. As shown in new Fig. 4G of the revised manuscript, the lifetime exhibited almost linear dependence over a certain range of size for fast- and slow-mobile states with the corresponding diffusion coefficients (0.062 and 0.012 $\mu m^2/s$, respectively) under no drug treatment. The lifetimes of the fast- and slow-mobile states were 0.046±0.000 and 0.468±0.008 sec, respectively (Table S5), for the patch sizes in Fig. 4G. This means that the sizes were around 50 and 250 nm for fast- and slow- mobile states, respectively.

To show the effects of the patch size on the lifetimes, in addition to new Fig. 4G in the revised manuscript, we have modified the main text (page 7, line 31 – page 8, line 1) and Methods (page 16, line 11-20).

Comment #1-i: *The deduced viscosity of the three membrane areas has a 95% confidence interval.*
Our reply: We thank Reviewer #2 for the comment. The estimated viscosities and 95% CI are described in the main test of the revised manuscript (page 6, line 28-30).

Comment #2: *The manuscript heavily depends on the evidence for three components. The AIC values for one, two, three and four components must be presented as supplemental data, including uncertainties as described in point 1).*
Our reply: We thank Reviewer #2 for the comment. Following the comments, we have added new Fig. S2D to show the AIC values with 95% CI for all protein species analyzed.

© 2023. Published by The Company of Biologists under the terms of the Creative Commons Attribution License (https://creativecommons.org/licenses/by/4.0/).

Comment #3: *The connection between equation 3 and Figure 2A is confusing. Equation 3 is a probability density function with dimension 1/μm. Figure 2A is a histogram with occurrence or probability and is dimension-less. So the experimental data of Fig 2A are divided by the bin size and then fitted using equation (3). This should be explained.*
Our reply: We apologize for the confusing explanation in the original manuscript. As described in our reply to **Comments #1-b and 1-c**, we do not use the histogram for the estimation of the diffusion parameters in the PDF analysis. We use the maximum likelihood estimation (MLE) to determine the diffusion coefficient $D_{PDF\text{-}state}$ and ratio $p_{PDF\text{-}state}$, where the parameters were determined using all Δr data without setting the bin size, as described previously (Matsuoka et al. (2009)).

The histogram of displacements (Δr) in Fig. 2A was overlaid with the product of the PDF (Eq. (3)) and bin size, which is dimensionless. Overlaid figures are shown also in Fig. S2A, B, C for the one-, two- and three-state models, respectively.

See also our reply to **Comments #1-b and 1-c**.

Comment #4: *Figure 2A needs further clarification. It shows experimental data (green) in bins; these data are observations and thus fraction or occurrence, not probability. The lines are the calculated probability.*
The dashed lines in Figure 2A are not explained in the legend.
Many readers will ask how the curve for two components will look like. As drawn, the curve is probably indistinguishable from the curve for three components, but may become distinguishable when the experimental data and the curves at ΔR>0.1 μm are enlarged in an inset.
Our reply: We thank Reviewer #2 for the helpful comment to improve our manuscript. We have added the explanation of the colored lines in the legend of Fig. 2 and Fig. S2 of the revised manuscript. Also, we have added the curve for one-, two- and three-component models and enlarged the figures in Fig. 2A of the revised manuscript. In addition, we have added Fig. S2A, B, C for the one-, two- and three-component models of all 27 protein species, respectively. In the two-state model, the fast and slow components are detected, and the fast component does not disappear for all 27 protein species (Fig. S2B).

Comment #5: *Indicate the pixel size in nm in line 46 on page 11.*
Our reply: The pixel size is 72 nm. We indicate the size in the Methods of the revised manuscript (page 10, line 48).

Comment #6: *Indicate the bin size in nm in line 4 on page 13 where the histogram is introduced.*
Our reply: The bin size is 3 nm. We indicate the size in the legend of Fig. 2A, Fig. S2 of the revised manuscript.

Comment #7: *From the information in the title and the abstract it is not immediately clear to the reader that the manuscript has experimental data and that the model is based on these data. This can be easily overcome by mentioning somewhere "experimental data" or "observed" single molecule tracks, etc.*
Our reply: We thank Reviewer #2 for the helpful comment. Following the advice, we have changed the title of the revised manuscript to, "**Field model for multistate lateral diffusion of various transmembrane proteins observed in living *Dictyostelium* cells**". Also, we have rewritten the sentence in the Abstract of the revised manuscript (page 2, line 12).

Comment #8: *The HMM model a priori assumes three components. Given that the fast component in the PDF has low abundance (~2%), the presence of the fast component in cell tracks is very rare. This is also noticed by the very short life-time of the fast component, suggesting that a fast section in a track has only a few steps. What is the evidence that the HMM model must have three components? How does the HMM model fit the data when only two components are used?*
Our reply: When we performed the bootstrap analysis for the datasets of displacements Δr, the fast-mobile component was detected in a reproducible manner (Fig. 2A). The same tendency was observed for all 27 proteins (Fig. S2B).

When we performed the HMM analysis with the two-component model, the estimated diffusion parameters for car1 provided a different set of diffusion coefficients, ratio and lifetimes from those estimated in the three-state model (please see the table below). However, the faster-

© 2023. Published by The Company of Biologists under the terms of the Creative Commons Attribution License (https://creativecommons.org/licenses/by/4.0/).

mobile state detected in the two-state model was a minor component with a short lifetime and large

Number of diffusion states	State	D_S (μm²/s)	P_S (%)	ε_S (nm)	Lifetime (s)
2	fast	0.041±0.006	11.2±0.5	87±5	0.068±0.002
	slow	0.022±0.000	88.8±0.5	7±2	0.573±0.010
3	fast	0.071±0.013	4.0±0.3	88±5	0.041±0.002
	middle	0.034±0.001	52.8±2.5	11±5	0.261±0.022
	slow	0.013±0.001	43.3±2.7	10±2	0.442±0.023

localization errors, which was similar with the fast-mobile state detected in the three-state model. In addition to the HMM analysis, the PDF analysis also detected the fast component for all 27 protein species (Fig. S2B), as described in our reply to Reviewer #2's **Comment #4**. Together with the results of the AIC (Fig. S2D), we concluded the three-state model is best.

Reviewer #1, **Comment #4** is related to this comment. Please also see our response to that comment.

Comment #9: *The transition between fast and slow component has not been observed. This is strange, and one of the arguments to have some doubt on the presence of a fast component, together with the calculated low abundance and low confidence of the fitted parameters. The only evidence for a fast component comes from the AIC of the PDF. Are more arguments with statistical support available?*
Our reply: We thank Reviewer #2 for the valuable comment. As described in our reply to Comment #1-e, the HMM analysis can detect the state transition successfully for the single molecule trajectory generated by the simulation. When there are no state transitions, the estimated values of the transition probability matrix of the HMM analysis were zero in most cases, as shown in Fig. S8D, E of the revised manuscript. When state transitions are assumed in the simulations, the transition probabilities are correctly estimated. Thus, the results of the HMM analysis provide evidence that there are state transitions.

We have added the results of the HMM analysis to the Methods (page 14, lines 14-19) and Fig. S8D, E.

Comment #10: *The field model in figure 4 and 7S is a nice attempt to summarize the data. However, it is strange to see squares for membrane patches; they are thermodynamically impossible. Can they be changed to circles?*
Our reply: As Reviewer #2 suggests, we also think that a square patch is unlikely from a thermodynamic point of view. We investigated the effect of changing the shape of the patches from squares to circles on the calculation results of the field model. As a result, we confirmed that the calculation results are almost the same when the area is kept the same (Fig. S6). Therefore, it is possible to change the figures of the field model shown as squares to circles. However, it would be easier for someone else to run the same simulation using our proposed model starting with a square. Thus, we decided to retain the square shape of the patches in this field model for simplicity and ease of repeatability of the model calculations.

To show the effect of changing the shape of the patches on the calculation results of the field model, we have added a description in main text (page 8, line 6-8), Methods (page 16, line 11-20) and new Fig. S6 in the revised manuscript.

Comment #11: *On page 13 the same symbol "capital P" is used for the probability density function in equation (3) and log likelihood in equation (4). Very confusing.*
Our reply: We apologize for confusing Reviewer #2. We have changed the symbol for log likelihood. The log likelihood, which is a function of the parameter θ, was defined in the revised manuscript as follows,

$$\mathrm{L}_n(\theta) = \sum_{m=1}^{M} \log \mathrm{P}_n(\Delta r_m|\theta) \qquad \text{Eq. (4)}$$

and added in the Methods (page 12, line 22).

© 2023. Published by The Company of Biologists under the terms of the Creative Commons Attribution License (https://creativecommons.org/licenses/by/4.0/).

Comment #12: *Estimates for diffusion rate constants D and relative fraction p of the tree components were obtained twice, from fitting PDF and with the HMM. Present both datasets including 95% confidential interval in the supplemental material for all proteins.*
Our reply: We thank Reviewer #2 for the helpful comment to improve our manuscript. Following the advice, we have added the datasets in Table S2 for the MSD analysis, in Fig. S2 for the PDF analysis, and Fig. S4 and Table S3-S5 for the HMM analysis in the revised manuscript.

In addition, we have changed the notation of the diffusion coefficient (D), ratio (p) and localization error (ε), which are summarized in Table S8 of the revised manuscript.

Comment #13: *The possible role of the cytoskeleton has been extensively studied and comprises a very large effort. The discussion on these data and how the cytoskeleton may affect diffusion is very limited. Is there more to say? Are among the 27 proteins membrane proteins known to interact with the cytoskeleton, e.g. connecting the plasma membrane with the F-actin cortex?*
Our reply: We thank Reviewer #2 for the comment. Of the 27 membrane proteins, no molecules are reported to interact directly with cytoskeleton molecules such as microtubules or F-actin. As for the possible roles of the cytoskeleton in the diffusion of membrane proteins, one might speculate three possibilities. One is a fence against the diffusion of membrane protein, which is well known in mammalian cells. Another is vesicular transport to the membrane, which would modulate the composition of lipids and other molecules in the membrane. Lastly, force generation by the cytoskeleton might affect the plasma membrane mechanically. Currently, we have no evidence to distinguish these possibilities, but the reduced diffusion by inhibition of the cytoskeleton suggests that cytoskeletal fences do not play a major role in the diffusion properties of membrane proteins in Dictyostelium cells, which is in sharp contrast to mammalian cells.

Due to the limitation in the number of words, we cannot add much discussion but still make note of these points in the main text (page 5, lines 14-15 and page 9, lines 12-15). Reviewer #1, **Comment #2** is related to this comment.

Second decision letter

MS ID#: JOCES/2022/260280

MS TITLE: Field model for multistate lateral diffusion of various transmembrane proteins observed in living Dictyostelium cells

AUTHORS: Kazutoshi Takebayashi, Yoichiro Kamimura, and Masahiro Ueda
ARTICLE TYPE: Short Report

I am happy to tell you that your manuscript has been accepted for publication in Journal of Cell Science, pending standard ethics checks. The referee comments on this version are appended below. Thank you for sending us your very interesting work.

Reviewer 1

Advance summary and potential significance to field

No further comments.
Comments for the author

No further comments.

Reviewer 2

Advance summary and potential significance to field

Important analysis of diffusion of membrane proteins

© 2023. Published by The Company of Biologists under the terms of the Creative Commons Attribution License (https://creativecommons.org/licenses/by/4.0/).

Comments for the author

The authors have addressed the questions, remarks and comments well, and the revised manuscript by Takebayashi is now acceptable for publication.

© 2023. Published by The Company of Biologists under the terms of the Creative Commons Attribution License (https://creativecommons.org/licenses/by/4.0/).
